# Plant Morphological, Physiological and Anatomical Adaption to Flooding Stress and the Underlying Molecular Mechanisms

**DOI:** 10.3390/ijms22031088

**Published:** 2021-01-22

**Authors:** Weitao Jia, Maohua Ma, Jilong Chen, Shengjun Wu

**Affiliations:** Chongqing Institute of Green and Intelligent Technology, Chinese Academy of Sciences, Chongqing 400714, China; jiawt@cigit.ac.cn (W.J.); mamaohua@cigit.ac.cn (M.M.); chenjilong@cigit.ac.cn (J.C.)

**Keywords:** flooding, morphology, physiology, anatomy, molecular mechanisms, phytohormone

## Abstract

Globally, flooding is a major threat causing substantial yield decline of cereal crops, and is expected to be even more serious in many parts of the world due to climatic anomaly in the future. Understanding the mechanisms of plants coping with unanticipated flooding will be crucial for developing new flooding-tolerance crop varieties. Here we describe survival strategies of plants adaptation to flooding stress at the morphological, physiological and anatomical scale systemically, such as the formation of adventitious roots (ARs), aerenchyma and radial O_2_ loss (ROL) barriers. Then molecular mechanisms underlying the adaptive strategies are summarized, and more than thirty identified functional genes or proteins associated with flooding-tolerance are searched out and expounded. Moreover, we elaborated the regulatory roles of phytohormones in plant against flooding stress, especially ethylene and its relevant transcription factors from the group VII Ethylene Response Factor (ERF-VII) family. ERF-VIIs of main crops and several reported ERF-VIIs involving plant tolerance to flooding stress were collected and analyzed according to sequence similarity, which can provide references for screening flooding-tolerant genes more precisely. Finally, the potential research directions in the future were summarized and discussed. Through this review, we aim to provide references for the studies of plant acclimation to flooding stress and breeding new flooding-resistant crops in the future.

## 1. Introduction

Along with climate changes, flooding shows a heavier tendency triggering severe crop reduction in both yield and quality around the world [1]. In the United States, for instance, losses of crop yield induced by flooding followed by drought from 2000 to 2011 [2]. In 2016, a total of 217 million dollars was paid for damages or controls associated with flooding, which cost 3.4 times higher than that for droughts (www.rma.usda.gov/data/cause). In some developing countries, because of their poor drainage systems, the impact of frequent flooding on crop systems is more serious, which usually aggravates poverty and food insecurity [1]. To reduce the losses, it is essential to unravel the mechanisms of plants against flooding stress for developing new flooding-tolerance crops.

**Figure 1:**
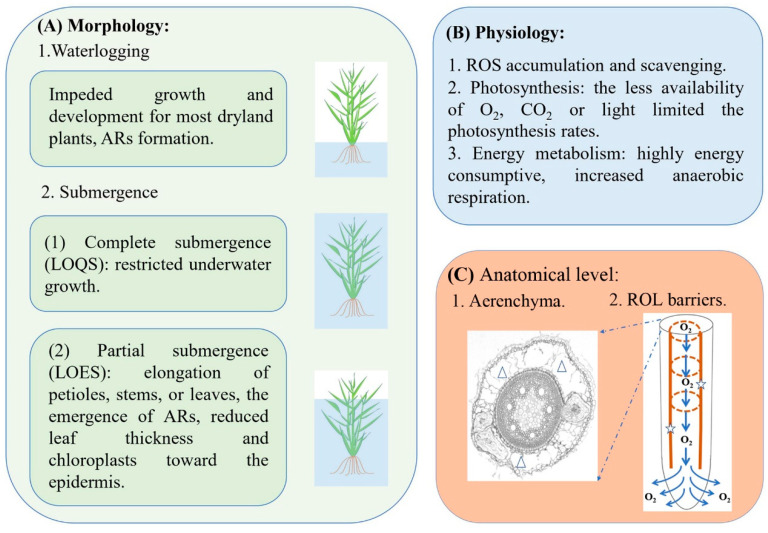
Response and the adaptive mechanisms of plants under flooding (hypoxic) stress. (**A**) Plants morphology under waterlogging and submergence stress. (**B**) The physiological changes of plants in response to flooding stress. (**C**) The main anatomical characteristics of plants adaptation to flooding stress. Triangles and pentagrams point to the location of aerenchyma and radial O_2_ loss (ROL) barriers respectively.

Generally, flooding can be classified into two forms depending on water depth: waterlogging and submergence [3,4]. Waterlogging is the condition that water exists on the soil surface and only plant roots are surrounded by water, while submergence is the state that the whole plant partially or completely immerses in water [3,5,6]. One of the immediate impacts caused by flooding is the deficiency of oxygen [7,8]. Previous studies showed that oxygen diffused in water approximately 10,000 times slower than that in air, and oxygen permeating into water-immersed soil is about 320,000 times less than that into soil full of gas [3,9,10]. The deficiency of oxygen in soil restricts plant growth resulting in the reduction of crop yield [3,11]. Under waterlogging stress, the growth of the plant was impeded due to enhanced anaerobic respiration [3,11]. Meanwhile, adventitious roots (ARs), aerenchyma and radial O_2_ loss (ROL) barriers emerged in roots for the exchange of gas [3,11]. When exposed to submergence stress, plants have evolved two main strategies to resist the adversity: low oxygen quiescence syndrome (LOQS) for complete submergence and low oxygen escape syndrome (LOES) for partial submergence [8,12]. Plants with LOQS strategy demonstrate restricted growth through keeping the minimum energy and carbon consumptions for prolonging underwater survival [13] (Figure 1A). After the flooding recedes, these plants can be recovered rapidly [11]. Escaping from water (LOES) is another strategy that plants maintain the upper leaves in the aerial environment for getting adequate oxygen, light and carbon dioxide [8,11]. Accordingly, several morphology and anatomical traits of plants change with the water depth, such as deepwater rice and *Rumex palustris* with internode or petiole elongation as well as aerenchyma formation [14,15,16] (Figure 1A).

Recently, much progress has been made in the mechanisms of plant acclimation to flooding stress [17,18,19]. For example, Nagai et al. reported two genes controlled internode elongation antagonistically in rice, the ‘accelerator’ *ACCELERATOR OF INTERNODE ELONGATION 1* (*ACE1*) and the ‘decelerator’ *DECELERATOR OF INTERNODE ELONGATION 1* (*DEC1*) [18]. Tang et al. showed that two transcription factors of the WRKY family (WRKY33 and WRKY12) interacted with each other to up-regulate *RAP2.2* for Arabidopsis adaptation to submergence stress [19]. Bui et al. reported that transcription factor ANAC017 mediated differential submergence tolerance of Arabidopsis between juvenile and adult stages [20]. These studies contribute to our understanding of the mechanisms of plant adaptation to flooding stress, however, the detailed update including these latest research progresses has not been conducted subsequently. In this review, plant survival strategies under flooding stress in morphology, physiology and anatomy levels were investigated and exhibited initially, then the potential crucial genes or proteins and the regulatory role of phytohormones were summarized by highlighting the most recent findings of plant adapting to flooding stress. Moreover, several key open questions worthy of further study were proposed and discussed.

## 2. Plant Responses to Flooding Stress

Flooding is a natural occurrence with adverse effects on plant growth and development, multiple mechanisms of plants adaption to flooding stress have been discovered at morphology, physiological and anatomical scales.

### 2.1. The Morphology of Plants under Flooding Stress

Generally, flooding stress triggers adaptive changes in plant roots and shoots at a morphological scale, such as the formation of ARs, the growth of the shoots inhibited or accelerated (Figure 1A). Previously, some important crops including wheat, maize, rice and soybean have been studied in their responses to flooding stress. For instance, wheat, as a dryland crop, was sensitive to flooding stress [21]. When exposed to waterlogging stress, dry mass of wheat shoot and root, and the ratio of root/shoot significantly declined compared to controls, indicating that root growth was inhibited more seriously than shoot [22,23]. Maize was also intolerant against waterlogging stress and the trefoil stage was the most sensitive period for maize [24,25]. Waterlogging inhibited maize growth resulting in decline in plant height, ear height, dry weight, leaf area index and grain characteristics (such as grain number per ear and 1000-grain weight) [26]. Meanwhile, contents of chlorophyll, soluble sugar and starch in leaves, stems and roots decreased under waterlogging stress [27]. For rice, many lowland cultivars are susceptible to submergence stress and thus hardly survive as the deluge lasts for a longer period of time [3,28,29]. However, several rice cultivars can experience flash-flood (complete submergence) for around two weeks by restricting shoot elongation as well as carbohydrate consumption, and will be recovered once the flash-flood recedes [5,11]. On the other hand, deepwater rice, which adapts to the submergence where it grows, is able to maintain the top leaves in the aerial environment for catching sufficient oxygen by elongating its internodes rapidly [5,30,31]. The growth and grain yield of soybean is also affected by flooding [32]. At the seedling stage, root growth of soybean was suppressed severely after submergence prolonging for 10 days [33]. Different soybean genotypes might utilize distinct mechanisms to resist waterlogging stress and proteins associated with energy metabolism were thought to function in soybeans tolerance to flooding stress [34,35]. Generally, waterlogging stress enhances anaerobic respiration of roots of dryland plants along with energy consumption and restricts their growth eventually. Likewise, complete submergence stress inhibits the growth of plants and decreases the rate of survival with the extension of the submergence time in most cases. By contrast, partial submergence promotes the elongation of internode, petiole or other organs in some plant species for getting enough oxygen, which contributes to their rapid growth in a short time.

Adventitious roots, as a typical trait of plants acclimating to waterlogging stress, can facilitate the uptake of water and nutrient as well as the transport of gas more effectively [36] (Table 1). ARs can be developed from some non-root tissues of the plants during normal development, or under the stimulation of external stresses, including flooding, nutrient deficiency, heavy metals and wounding [37]. On the basis of physical characteristics and generation conditions, ARs can be divided into various types, such as hypocotyl roots, crown roots, brace roots, nodal roots, stem roots, junction roots and prop roots, among which the front five types can be triggered by flooding [37]. When exposed to waterlogging stress, maize formed more crown roots than controls and the waterlogging-tolerant line possessed more crown roots than the waterlogging-sensitive line [38]. In deep-water rice, the elongation of ARs was initiated after 8 to 10 h under partial submergence stress, and ethylene was found to facilitate the formation of ARs through triggering the death of epidermal cells covering the root tip [39,40]. In tomato, ARs gradually formed at the hypocotyl after three days of partial submergence, and further extended to the upper surface of the water along caulicles [41]. Furthermore, inhibiting ethylene or auxin production impaired ARs formation of tomato, which indicated that ethylene and auxin might interact with each other during the development of ARs under flooding stress [42]. Likewise, a recent study reported that waterlogging stress-induced cucumber generating a large amount of ARs, and both auxin and ethylene can stimulate the formation of ARs, however, auxin-induced ARs relied on ethylene while ethylene-induced ARs was unaffected by auxin [43]. Contrastively, the opposite result was found in *Rumex palustris* that auxin-induced ARs were not affected by inhibiting ethylene biosynthesis while the inhibition of auxin transport suppressed ethylene-induced ARs formation [44]. In summary, the formation of plant ARs is mainly regulated by ethylene and auxin under flooding stress, however, their roles may be diverse in different plants.

### 2.2. The Physiological Phenotype of Plants Response to Flooding Stress

Flooding stress leads to accumulation of the harmful substances in plants, such as reactive oxygen species (ROS) and malondialdehyde (MDA) [67]. In turn, plants activate the internal protection mechanisms to scavenge these components, such as the production of proline, glutathione and the activation of antioxidant enzymes [57,68,69] (Figure 1B, Table 2). ROS are toxic byproducts of oxidative metabolisms, such as O_2_^.−^, H_2_O_2_, OH˙ and ^1^O_2_ [70,71]. Whereas, ROS also functions as signal molecules that regulate plant development under environmental stresses [56,71]. Submergence exhibited a huge impact on ROS homeostasis. Ye et al. reported that submergence led to a decrease of H_2_O_2_ content in bermudagrass (*Cynodon dactylon*) leaves, though O_2_^.−^ content had insignificant change [68]. Contrastively, another study showed that O_2_^.−^ and H_2_O_2_ concentration in the elongation and mature zone of barley roots increased under waterlogging stress [72]. Additionally, MDA content in bermudagrass leaves increased after submergence for three weeks and catalase (CAT), glutathione reductase (GR) and peroxidase (POD) activities presented an upward trend with the extension of the time [68]. Wang et al. showed the activities of antioxidative enzymes POD and superoxide dismutase (SOD) in roots of *Triarrhena sacchariflora* increased first and then decreased under flooding stress as well as contents of proline and MDA compared with control plants [69].

On the other hand, flooding is accompanied by the deprivation of oxygen, which leads to aerobic respiration in a low-level and accelerates more carbohydrates consumed by glycolysis [85]. Moreover, under complete submergence stress, the insufficiency of carbon dioxide and light decreased plant photosynthesis rates [13,86] (Figure 1B). For instance, the accumulation of soluble sugar and sucrose in bermudagrass leaves declined obviously under submergence stress [68]. Loreti et al. reported that starch content in rosette leaves of Arabidopsis degraded with the extension of submerged time during the night, and glucose content was observed decline at the end of the night as well as sucrose [87]. For spring maize, the negative impact induced by waterlogging stress was different because of the distinction of duration and growth stage, and the seedling stage was the sensitive period followed by the jointing and tasseling stages [88]. Additionally, the photosynthetic rate (Pn) decreased with the waterlogging time prolonging and resulted in the reduction of total dry weight and grain yield ultimately [88].

### 2.3. Anatomical Adaptations to Flooding Stress

Flooding often leads to oxygen deprivation in soils and plant roots, which significantly restricts metabolic activities for plant survival and development. Therefore, amounts of aerenchyma and ROL barriers in plant roots are formed to transport adequate oxygen from shoots to roots (Figure 1C).

#### 2.3.1. The Formation of Aerenchyma

Aerenchyma not only exists in many plants constitutively, but also is formed inductively by environmental stress, such as flooding stress [89] (Table 1). It forms in roots and shoots and consists of thin-walled cells filled with gas spaces to facilitate the diffusion of oxygen [8]. For instance, Aerenchyma formed in the mid-cortex cells of wheat roots after waterlogging for 24 h and its formation was mediated by ROS [55,56]. When applied with an ethylene precursor beforehand, wheat seedlings formed lysigenous aerenchyma in roots and these aerenchyma were further developed under hypoxic stress [90]. *Zea nicaraguensis* is a wild relative of maize and grew in frequently flooded areas [49]. Soil waterlogging increased aerenchyma proportion in the cross-sectional area of maize root from less than 1% in drained soil to 15% as well as from 22% to 29% in *Z. nicaraguensis* roots [49]. Another study reported that ARs of waterlogging-tolerant barley (*Hordeum vulgare* L.) genotypes exhibited remarkably higher porosity and formed aerenchyma more quickly compared to sensitive ones [57]. Aerenchyma increases plant tolerance to flooding stress by improving the oxygen-transport efficiency from shoot to root tip, and its formation mainly relies on ethylene and ROS signaling.

#### 2.3.2. A Barrier to Radial Oxygen Loss

Except for aerenchyma, the oxygen-transport efficiency from shoot to root tip depends on another crucial factor, the formation of ROL barriers. ROL barriers usually form in the roots of waterlogging-tolerant plants, and can reduce oxygen leakage during its transport from shoot-to-root tip and impede soil phytotoxin entry simultaneously [49,51]. The ROL barriers can be formed constitutively or induced by stagnant conditions, such as flooding stress [14,91] (Figure 1C, Table 1). Many wetland species, such as *Oryza sativa*, *Juncus effusus*, *Phragmites australis* and Glyceria *maxima*, have been reported forming ROL barriers in roots [14,15,92,93]. For example, growing in stagnant nutrient solution with oxygen removal enhanced the development of ROL barriers in ARs of rice [15,45]. Long ARs of rice initiated the formation of ROL barriers quickly and developed well within 24 h under hypoxic conditions, however, short roots generated the barrier for more than 48 h [93]. The dryland species have the ability to form ROL barriers under flooding stress. For instance, seminal roots of wheat induced ROL barriers when exposed to waterlogging to enhance the internal supply of oxygen [56]. Notably, some *Echinochloa* species even developed a ROL barrier constitutively under aerated conditions [66].

The formation of ROL barriers can also be triggered by the organic acids [94,95]. In waterlogged soils, some monocarboxylic acids are produced by anaerobic microorganisms, such as acetic, butyric, hexanoic and propionic acids, which inhibit plant growth as accumulated to some extent and can invoke enhanced suberization or lignification in cell walls of roots [96,97]. For example, the ROL barrier in *Hordeum marinum* roots was induced by butyric and hexanoic acids individually or a mixture with acetic and propionic acids [94], and these four organic acids also triggered the ROL barrier in roots of rice [95].

## 3. Molecular Mechanism of Plant Response to Flooding Stress

On the basis of the morphology, physiology and anatomy characteristics of plant response to flooding stress, many associated-genes functioning in plant adaption to flooding stress have been reported and studied (Table 3).

### 3.1. The Formation of Adventitious Roots

**Figure 2:**
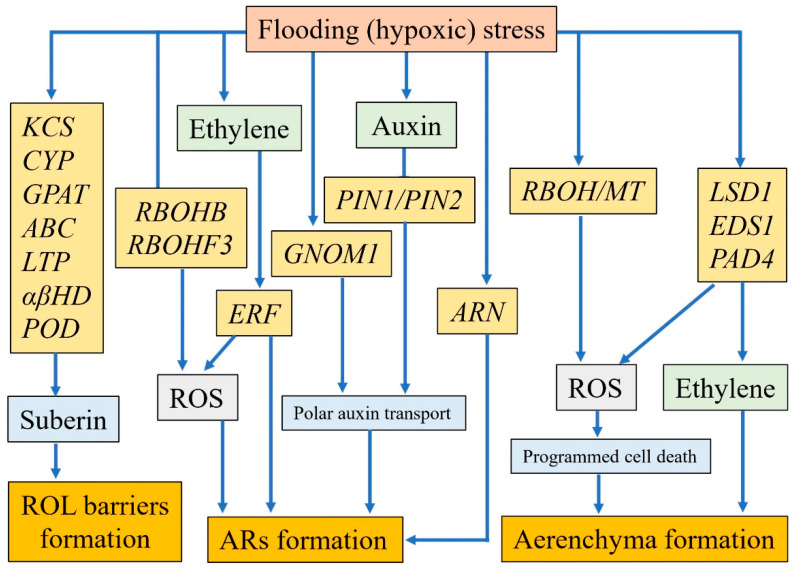
The molecular mechanisms of ROL barriers, ARs and aerenchyma formation. Researches about the formation of flooding-induced ROL barriers at the molecular level are relatively deficient, however, suberin has been considered to contribute to the formation of ROL barriers and genes (such as *KCS*, *CYP*, *GPAT*, *ABC*, *LTP*, *αβHD*, *POD*) involved in suberin biosynthesis may function in this process [48]. For ARs formation, flooding (hypoxic) stress can induce expressions of *RBOHB*, *RBOHF3*, some *Ethylene Response Factor* (*ERF*) genes and *ARN*, then affects ARs formation directly or through ROS regulation [25,43,117]. Plants also can influence the development of ARs by mediating auxin polar transport through the activation of some auxin associated genes, such as *GNOM1*, *PIN1* and *PIN2* [120,125,126]. Aerenchyma formation depends on the accumulation of ROS. *RBOH* for the generation of ROS and *MT* for ROS scavenging play important roles in this process [53]. In addition, genes *LSD1*, *EDS1* and *PAD4*, which control the generation of ethylene and ROS in the upstream, are also involved in the formation of aerenchyma [62].

The emergence of ARs is one of the adaptive strategies for plants coping with flooding stress, and several genes have been identified to mediate ARs formation (Figure 2). *GNOM* encodes a guanine-nucleotide exchange factor for ADP-ribosylation factor (ARF) [120] and can influence polar auxin transport in plants [121,122]. Studies have reported that *OsGNOM1* in rice affected the development of ARs through regulating polar auxin transport, and loss function of this gene resulted in the defect of ARs formation [120]. *CsARN6.1* in cucumber encoded an AAA ATPase domain-containing protein and affected waterlogging tolerance through the regulation of ARs formation, besides, the transformation of *CsARN6.1* from waterlogging-tolerant cultivar into waterlogging-sensitive variety increased numbers of ARs under waterlogging conditions [117]. Ethylene can induce ARs formation and this process requires ROS as a signal triggering programmed cell death in cortex cells [123,124]. For instance, waterlogging led to the accumulation of ethylene and ROS in cucumber, ethylene-induced AR formation was inhibited by the reduction of H_2_O_2_ [43]. The above results suggest that auxin, ethylene and ROS are required for the formation of ARs under flooding stress, but the mechanisms of interaction among them need to be revealed.

### 3.2. The Homeostasis of Reactive Oxygen Species

Flooding stress leads to oxidative stress for plants, which triggers plant antioxidant systems rapidly [68,77]. *Respiratory burst oxidase homolog* (*RBOH*) encodes a plasma membrane-associated NADPH oxidase in plants and can generate hydrogen peroxide (H_2_O_2_) by converting O_2_ to O_2_^·−^ [127,128,129]. Qi et al. reported that cucumber accumulated higher levels of ROS under waterlogged stress, further investigation showed that two members *CsRBOHB* and *CsRBOHF3* were strongly upregulated and might result in ROS accumulation [43]. Hypoxia Responsive Universal Stress Protein 1 (HRU1) can be induced by submergence stress in Arabidopsis, and was regulated by an VII Ethylene Response Factor (ERF-VII) protein RAP2.12 (Related to Apetala 2.12) simultaneously; loss of HRU1 function affected H_2_O_2_ production and became sensitive to submergence and anoxia, further research exhibited that HRU1 mediated ROS production in Arabidopsis through an interaction with the GTPase ROP2 and the NADPH oxidase RbohD [130]. The transcriptome of two *Brachypodium distachyon* ecotypes with contrasting submergence tolerance exhibited that lots of genes associated with redox reaction significantly changed tolerant ecotype, such as genes coding for ascorbate peroxidases (ASP), ascorbate oxidases (ASO) and peroxidases (PER), which might be involved in ROS management under submergence stress [131]. ROS accumulation is harmful for plants, on the other hand, it mediates ARs and aerenchyma formation as signaling molecules. Under flooding stress, how plants keep the balance between oxidative stress and signal transduction needs to be further explored.

### 3.3. The Mechanism of Aerenchyma Formation

Generally, aerenchyma can be classified into two types: schizogenous aerenchyma and lysigenous aerenchyma [132]. Schizogenous aerenchyma that forms gas spaces owing to cell separation and expansion without cell death, by contrast, lysigenous aerenchyma is formed due to the death and subsequent lysis of some cells [53]. Under flooding stress, plants usually form lysigenous aerenchyma in roots to improve oxygen diffusion (e.g., Arabidopsis, maize and wheat) and several genes involved in aerenchyma formation have been investigated [53,62,90] (Figure 2). In Arabidopsis, lysigenous aerenchyma emerged in hypocotyls under hypoxic stress and this process relied on three genes *LSD1*, *EDS1* and *PAD4*, which regulated the upstream ethylene signal and the generation of ROS [62]. With laser microdissection technology, Rajhi et al. investigated the gene expression profile of cortical cells in maize root when the lysigenous aerenchyma formed, and showed that the expression of *RBOH* gene increased sharply under waterlogging stress while *MT* (*metallothionein*) gene for scavenging ROS was down-regulated, which may lead to the generation of excess ROS and triggered programmed cell death in cortical cells for aerenchyma formation [53]. Additionally, ethylene-induced the formation of aerenchyma under waterlogged conditions [53]. In wheat roots, an ethylene precursor pre-treatment enhanced the expression of three *RBOH* genes for ROS generation and activated the formation of lysigenous aerenchyma under stagnant conditions, which might be one of the strategies for wheat seedlings acclimation to hypoxia stress [90]. According to the above researches, it can be concluded that both ethylene and ROS mediate the formation of aerenchyma. Ethylene may induce the generation of ROS and results in the programmed cell death of cortical cells for lysigenous aerenchyma formation eventually.

### 3.4. The Forming Basis of ROL Barriers

Apart from aerenchyma, plants develop ROL barriers in roots to impede oxygen leakage and several genes have been reported to mediate its formation [36] (Figure 2). Previous studies exhibited that suberin might be the major component of ROL barriers compared to lignin. For example, De Simone et al. showed that suberization but not lignification of exodermal cell walls suppressed the radial oxygen loss effectively in roots of four Amazon tree species, indicating it was suberin forming the ROL barriers under flooding stress [133]. Shiono et al. reported that the expression of most genes associated with suberin biosynthesis increased sharply in the outer part of rice roots during ROL barrier formation, including *GLYCEROL-3-PHOSPHATE ACYLTRANSFERASE* (*GPAT*) and *PEROXIDASE* (*POD*), which have also been identified to be upregulated during the formation of ROL barriers with Si supply [48,134], by contrast, few genes related to lignin biosynthesis were found in this process [88]. Additionally, some transcription factors containing WRKY, AP2, MYB, and NAC domains might participate in the regulation of suberin biosynthesis during ROL barrier formation [48,135,136,137] (Figure 2). Although suberin is considered to be more crucial in forming the ROL barrier of plant roots, the initiation and regulation mechanisms of suberin deposition remain unclear.

## 4. Regulatory Mechanisms of Phytohormones in Plants Response to Flooding Stress

**Figure 3:**
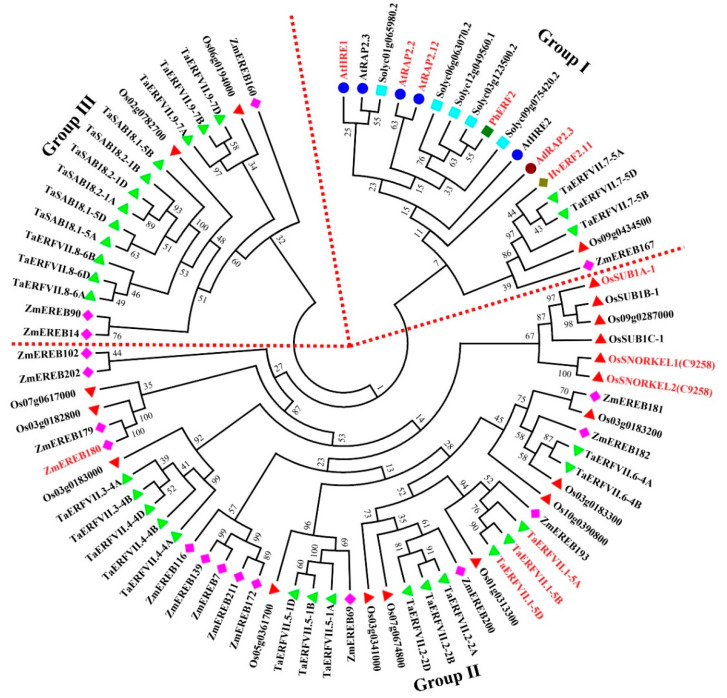
Phylogenetic tree of ERF-VIIs from different plant species. These plant species include model plant Arabidopsis, main crops (rice, maize, wheat, tomato) and other species with ERF-VII members that have been reported. The coding sequences of these *ERF-VII* genes are listed in Supplemental file 1. The tree was constructed using a neighbor-joining method with 1000 bootstraps in Mega 7 [142]. *ERF-VII* genes that have been identified to function in flooding-tolerance are marked in red font. Picture shapes with various colors represent ERF-VIIs from different species. 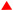, rice; 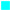, tomato; 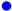, Arabidopsis; 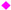, maize; 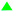, wheat; others picture shapes, ERF-VIIs that have been studied.

Phytohormones play critical roles in regulating plant growth and can integrate multiple signal transduction pathways under environmental stresses [138,139]. Some phytohormones have been identified to be involved in plants adapting to flooding stress, such as ethylene, gibberellin and auxin [13] (Table 3, Figure 2 and Figure 3).

### 4.1. Ethylene

Ethylene is a phytohormone in the form of gas, and hardly escape from plants under flooding conditions, which led to its rapid accumulation inside the plant [13]. The increased ethylene promotes the formation of AR and aerenchyma in plants when exposed to flooding stress [43,90] (Figure 2). ERF-VIIs are ethylene-responsive transcription factors with a conserved APETALA2 (AP2) domain and have been identified to function in plants tolerance to waterlogging and submergence stresses [17,25,31,104,140] (Table 3). Two ERF-VII members SK1 and SK2 from deepwater rice were transcriptionally induced by submergence stress and contributed to the elongation of internodes so as to keep the top leaves from submerging [11,31]. Another ERF-type transcription factor *Sub1A*, was inducible expression under submergence stress, and overexpression of *Sub1A* with a tolerance-specific allele in submergence-intolerant variety enhanced its submergence tolerance [104]. ERF-VII member *TaERFVII.1* from waterlogging-tolerant wheat (*Triticum aestivum*) was triggered expression obviously by waterlogging but not in susceptible cultivar, overexpression of *TaERFVII.1* increased the waterlogging-tolerance ability of wheat with no adverse effect on its growth and grain production [17], which can be considered as a candidate gene for breeding waterlogging-resistant crops. Yu et al. reported that an ERF-VII member from maize, ZmEREB180, was concerned with waterlogging tolerance and its expression level was affected by the changes of 5ʹ-untranslated region (5′-UTR); transgenic maize of *ZmEREB180* increased the survival rate through promoting ARs formation and improving antioxidant ability, which was further supported by transcriptomic analysis [25]. As transcription factors, ERF-VIIs usually function by affecting the expression of other genes, however, they are regulated by other transcription factors simultaneously. Recently, Tang et al. reported two transcription factors of WRKY33 and WRKY12 were involved in Arabidopsis adaptation to submergence stress [19]. WRKY33 interacted with WRKY12 and both of them participated in hypoxia response partially by activating the expression of ERF-VII member *RAP2.2* [19]. In addition, overexpression of *WRKY33* or *WRKY12* in Arabidopsis enhanced resistance to hypoxia [19].

Considering the importance of ERF-VIIs for plant adaption to flooding stress, their members in the model plant Arabidopsis, main crops (rice, maize, wheat and tomato) and other reported ERF-VIIs were collected and constructed a phylogenetic tree. These members can be divided into three groups according to sequence similarity. ERF-VIIs that had been reported to be involved in plant tolerance to flooding stress belongs to Group Ⅰ and Ⅱ, no genes associated with flooding-tolerance have yet been found in Group Ⅲ (Figure 3), which can provide references for screening flooding-tolerant genes more precisely. In brief, ethylene as a major regulator mediated the formation of ARs, aerenchyma, shoot hyponasty and elongation [141]. However, the downstream genes of ethylene-signaling pathway associated with flooding-tolerance are largely unknown. For example, ERF-VIIs as transcription factors, usually function by regulating the expression of their target genes, but so far, lots of these upstream genes have not been found and studied.

### 4.2. Auxin

Auxin, which acts as a general coordinator of plant growth and development, has been reported to participate in several plant species adapting to waterlogging stress (Figure 2), such as box elder (*Acer negundo*) [143], tomato [42], sunflower [144] and cucumber [43]. Auxin functions in the initiation of root apical meristems and increased auxin content may result in the formation of AR primordia [43,145,146]. *OsPIN1* is a polar auxin transporter from rice and plays an important role in auxin-dependent ARs formation; the loss function of *OsPIN1* suppressed the development of ARs apparently and application of auxin partially rescued the missed phenotypes [125]. Another polar auxin transporter PIN2 of *Solanum dulcamara* could be induced by flooding stress and ethylene application, the silence of *PIN2* or impediment of auxin transport with chemical methods inhibited AR primordium initiation [126]. Gutierrez et al. reported that three genes of AUXIN RESPONSE FACTOR (ARF) family regulated ARs initiation cooperatively in Arabidopsis [147]. ARF6 and ARF8 were positive regulators while ARF17 regulated ARs formation negatively. Meanwhile, three auxin-induced *Gretchen Hagen3* (*GH3*) genes (*GH3.3*, *GH3.5*, and *GH3.6*) for the biosynthesis of acyl-acid-amido were necessary for ARs initiation in Arabidopsis hypocotyl [148]. All of these studies indicated that auxin signal participates in plant acclimation to flooding stress mainly through regulating the formation of ARs.

### 4.3. Gibberellin

**Figure 4:**
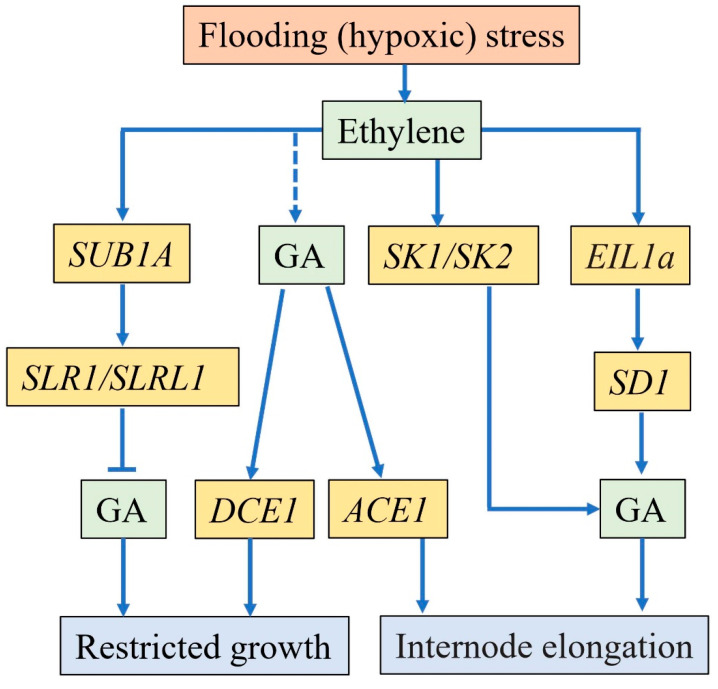
The molecular mechanisms of plants with the ‘quiescent’ (LOQS) and ‘escape’ (LOES) strategies as exposed to submergence stress. Generally, flooding stress induces the accumulation of ethylene and then leads to different responses according to flooding depth. Under complete submergence, the ethylene response factor SUB1A is induced and represses the gibberellic acid (GA) signal to prevent shoot elongation through SLR1 and SLRL1 [104,153]. Ethylene accumulation induced by partial-submergence contributes to the expression of the SK1 and SK2, which are involved in the process of internode elongation via GA signal [31]. Ethylene-responsive transcription factor EIL1a can activate the expression of *SD1* (a gibberellin biosynthesis gene), then increased the synthesis of gibberellins to promote internode elongation [110]. Additionally, an ‘accelerator’ ACE1 and a ‘decelerator’ DEC1 antagonistically regulate internode elongation together with gibberellic acid, expression of *ACE1* or downregulation of *DEC1* contributes to the internode elongation [18]. The dotted arrow represents that GA may be induced by flooding stress directly without being regulated by ethylene.

Gibberellin was also identified to function in plant adaptation to flooding stress (Figure 4). As suffered from flash floods, submergence-tolerant plants usually restrict their growth by activation of GA signal [149]. Slender Rice-1 (SLR1) and SLR1 Like-1 (SLRL1) are GA signaling repressors in rice [150,151,152]. Previous research exhibited that *Sub1A* promoted the accumulation of SLR1 and SLRL1 to suppress GA signal transduction in submergence-tolerant rice under submergence stress, and affected the expression of *PDC* and *ADH* genes positively, which led to the inhibited growth of submergence-tolerant rice for energy conservation and regrowth after flooding [104,149,153]. GID1 (GIBBERELLIN INSENSITIVE DWARF1) is a soluble GA receptor and has been reported previously [154]. Under submergence conditions, loss function of GID1 in rice inhibited the degradation of chlorophyll and promoted the metabolism of carbohydrates, further analyses suggested that GID1 regulated GA signals to influence submergence tolerance through controlling carbohydrate consumption [155].

GA also plays an important role in the stem elongation of plants [156]. In lowland rice, an increase of active GA (GA1) resulted in the elongation of leaf sheath under submergence stress [157]. For deepwater rice, submergence induced the expression of gibberellin biosynthesis gene *SD1* (*SEMIDWARF1*) that was regulated by an ethylene-responsive transcription factor OsEIL1a, and internode elongation was promoted by increasing gibberellins [110]. Recently, two genes, an ‘accelerator’ *ACE1* and a ‘decelerator’ *DEC1*, were reported to regulate internode elongation antagonistically together with GA; *ACE1*, as an unknown function protein, contributed internode elongation in concert with GA while *DEC1*, as a zinc-finger transcription factor, had the opposite function that restricted internode elongation; these two genes were involved in deepwater rice acclimation to submergence stress collectively [18]. According to the above results, GA mainly functions in plant acclimation to flooding stress through the modulation of carbohydrate metabolism and the regulation of internode (or other organs) elongation. Actually, GA mediates flooding-tolerance of plants together with ethylene in most cases.

### 4.4. Other Phytohormones

Moreover, cytokinin and abscisic acid (ABA) have been identified to function in plants against flooding stress [114,158,159]. Waterlogging-induced ARs formation in wheat is relevant to four phytohormones (IAA, GA, cytokinin and ABA) and several genes associated with their biosynthesis or metabolism were inducible expression under waterlogging stress [160]. Salicylic acid (SA) also participates in the waterlogging-tolerance of plants. SA content in waterlogging-tolerant soybean lines increased significantly after waterlogging for 5 or 10 days compared to non-waterlogging conditions while SA content in sensitive lines exhibited no significant change, implying that SA mediates waterlogging-tolerance of soybean through regulating the formation of aerenchyma or ARs [161]. Another phytohormone brassinosteroid (BR) has been reported that it may affect GA signaling by interacting with Sub1A, and regulated the elongation of shoot for surviving under submergence condition [162].

In fact, different phytohormones function cooperatively in response to flooding stress. For instance, the ethylene response factor Sub1A in rice repressed the GA signal to prevent shoot elongation and carbohydrate consumption under complete submergence [104,149]. However, when exposed to partial submergence, ethylene accumulation contributed to the expression of *SK1* and *SK2*, which facilitated internode elongation through regulating GA biosynthesis or GA signal transduction [31]. Notably, the expression of *SK1* and *SK2* may be regulated by one transcription factor EIN3 (ethylene-insensitive-3-like protein) through binding to their promoter regions [31]. To summarize, plant response to flooding stress is a complicated process depending on the co-regulation of multiple phytohormones and the cooperation mechanisms among different hormones need to be further revealed. Meanwhile, the studies about SA and BR regulating flooding-tolerance of plants should be strengthened in the future.

## 5. Conclusions and Further Perspectives

Recently, the mechanisms of plant adaption to flooding stress have been progressively studied. However, there are still several key open questions to be further clarified.

Firstly, since former studies of plants adaption to flooding stress excessively focus on model or wetland plants including Arabidopsis, rice and *Rumex*, the terrestrial plants should be paid more attention in the future, such as wheat, maize, barely, tomato and soybean. More importantly, it is essential to collect germplasm resources of these terrestrial plants widely owing to phenotypic variation among natural populations, and then the key loci associated with flooding-tolerance can be screened through map-based cloning, genome-wide association study (GWAS) and precision genome editing with the aid of CRISPR-Cas9 system for breeding flooding-resistant varieties.

Secondly, the uptake of multiple mineral nutrients usually becomes disorganized under flooding stress. For example, the concentrations of N, P, K and Zn in leaves of *Lepidium latifolium* L. exhibited a decreasing trend as the flooding-time prolongs, and were lower than those in control plants, however, Fe and Mn contents were higher in roots of flooded plants than that in unflooded ones [163]. Mineral elements are crucial for plant growth and the deficiency of mineral nutrients under flooding conditions hastens adverse effects on plants. How plants maintain mineral nutrients homeostasis under waterlogging or submergence stress is largely unknown. Additionally, the formation of ROL barriers is one of the typical strategies for plant acclimation to flooding stress, however, its prevention of oxygen loss is nonspecific, and the uptake of water or nutrients is influenced simultaneously [164,165]. Thus, the mechanism of plants balancing the impediment of oxygen loss and the uptake of water or nutrients also needs to be further investigated.

Thirdly, flooding stress usually leads to changes in metabolic activities in plants, especially outbreaks of some harmful substances, such as ROS, MDA and acetaldehyde. For instance, flooding-induced oxygen deprivation facilitates the activation of anaerobic respiration and results in the generation of ethanol and acetaldehyde. Ethanol is usually considered to be innocuous for its rapid diffusion from cells while acetaldehyde can poison plant cells [7]. Under the prolonged flooding stress, how does the plant avoid the toxic of acetaldehyde? Whether the flooding-tolerance plants have enhanced capacity or other pathways to scavenge acetaldehyde? These questions remain to be further answered.

Finally, the mechanisms about plants with the ‘quiescent strategy’ adaption to submergence stress remain to be further investigated, especially the tolerant species that can endure complete submergence stress for a long time. For instance, more than 90% of bermudagrass can survive after complete submergence for 5 months with water depth up to 25 m [166]. Under deep submergence, bermudagrass experiences multiple stresses simultaneously, including oxygen deprivation, low temperature, high water pressure and low light or even no light. How does bermudagrass adapt to the stresses over the long term? Exploring the above scientific questions will facilitate understanding the mechanisms of plant acclimation to flooding stress and provide new insights into the breeding of flooding-resistant crops in the future.

## Figures and Tables

**Table 1:** The formation of adventitious roots (ARs), aerenchyma and ROL barriers in plants responding to flooding stress.

Flooding Stress (Real or Simulated)	Species	Adventitious Root	Aerenchyma	ROL Barrier	References
Under stagnant deoxygenated conditions	Rice	Y *	Y	Y	[45]
Partial submergence	Rice	Y			[39,40]
Under stagnant deoxygenated conditions	Rice		Y	Y	[15]
Under stagnant deoxygenated conditions	Rice			Y	[46]
Partial submergence	Rice		Y		[47]
In aerated, stagnant deoxygenated, or N_2_-flushed conditions	Rice			Y	[48]
Under stagnant deoxygenated conditions	Teosinte	Y	Y	Y	[49]
Waterlogging	Teosinte		Y		[50]
Under stagnant deoxygenated conditions	Teosinte	Y		Y	[51]
Waterlogging	Maize and teosinte	Y	Y		[52]
Waterlogging	Maize		Y		[53]
Waterlogging	Maize	Y			[38]
Waterlogging	Maize	Y			[25]
Waterlogging	Wheat		Y		[54]
Waterlogging	Wheat		Y		[55]
Waterlogging	Wheat		Y	Y	[56]
Under stagnant conditions	*Hordeum marinum*, *Triticum aestivum*, *Hordeum marinum* × *Triticum aestivum*	Y	Y	Y	[23]
Waterlogging	Barley	Y	Y		[57]
Flooding (Waterlogging)	Rumex		Y		[58]
Waterlogging	Rumex	Y			[44,59]
Waterlogging	Sunflower	Y			[60]
Waterlogging or hypoxic culture	Willow	Y	Y		[61]
Waterlogging	Arabidopsis		Y		[62]
Flooding (Waterlogging)	Tomato	Y			[42]
Partial submergence	Bittersweet	Y			[63]
Half-flooding (Partial submergence)	Taxodium	Y	Y		[64]
Waterlogging	Cucumber	Y			[43]
Waterlogging	Seashore paspalum, Bermudagrass		Y		[65]
Waterlogging	Some *Echinochloa* species			Y	[66]

* ‘Y’ represents ‘Yes’ and means plants have the corresponding trait.

**Table 2:** Changes of ROS, antioxidant enzymes and other physiologic indexes in plant tissues as exposed to flooding stress.

Plant Species	Samples	ROS	Others	Antioxidant Enzymes	Reference
Barley	Leaf			CAT ↑ *, ascorbate peroxidase (APX) ↑ and POD ↑; SOD ↓ **	[73]
Barley	Root	H_2_O_2_ ↑, O_2_^.−^ ↑			[72]
Rice	Root and shoot			SOD ↑ and CAT ↑, APX ↓	[74]
Wheat	Leaf, root and shoot		Leaf water potential ↓, stomatal conductance ↓, photosynthesis ↓, chlorophyll content ↓, shoot nitrogen content ↓, shoot and root growth ↓, the diameter of metaxylem and protoxylem vessels of the nodal roots ↓		[54]
Wheat	Flag leaf			POD ↑ in tolerant genotypes, SOD ↓ and CAT ↓	[75]
Maize	Leaf			SOD ↑, CAT ↑ and APX ↑, POD ↓	[76]
Citrus	Leaf and root		Leaf MDA (early stage) - ***, Leaf MDA (15–27 days after waterlogging) ↑; Root MDA ↑ or -; Leaf proline ↑ or -.	SOD ↑	[77]
Citrus	Leaf			SOD ↑, CAT ↑	[78]
Tobacco	Leaf			SOD↑	[79]
Lotus	Shoot			SOD ↓, dehydroascorbate reductase (DHAR) ↓, GR ↓, APX ↑, monodehydroascorbate reductase (MDAR) -	[80]
Creeping bentgrasss	Root	H_2_O_2_ -	MDA -	SOD ↑, AR had no consistent trend with different genotypes. GR -, POD -	[81]
Pigeon pea	Root	Superoxide radical, H_2_O_2_	Visible yellowing and senescence of leaves; leaf area, dry matter, relative water content and chlorophyll content in leaves, and membrane stability index in roots and leaves decreased.	SOD ↑, CAT ↑, GR ↑ and APX ↑	[82,83]
Bermudagrass	Leaf	H_2_O_2_ ↓, O_2_^.−^ -	Proline -	POD ↑, GR ↑, CAT ↑	[68]
Perennial ryegrass	Shoot and root		Leaf chlorophyll and total carotenoid ↓, water-soluble carbohydrate (shoots and roots) ↓, MDA ↑	CAT and POD (shoot) ↑, SOD, CAT, POD and APX (root) ↓	[84]
Soybean	Leaf	H_2_O_2_ content increased at 7 days of waterlogging.	Activities of lactate dehydrogenase (LDH), alanine aminotransferase (AlaAT), alcohol dehydrogenase (ADH) and pyruvate decarboxylase (PDC) changed inconsistently in different genotypes under waterlogging stress.	SOD activity increased after 7 days of waterlogging, APX and CAT activities changed with no consistent trend among different genotypes or conditions.	[35]

* ‘↑’ represents ‘increase’; ** ‘↓’ represents ‘decrease’; *** ‘-’ represents ‘no change’.

**Table 3:** General descriptions of recently reported genes and the relative phytohormones involved in plant response to flooding stress.

Flooding Type	Species	Genes Name	Function	Phytohormone	References
Waterlogging	Arabidopsis	*LSD1*, *EDS1* and *PAD4*	These three genes *LESION SIMULATING DISEASE1* (*LSD1*), *ENHANCED DISEASE SUSCEPTIBILITY1* (*EDS1*) and *PHYTOALEXIN DEFICIENT4* (*PAD4*) controlled the formation of lysigenous aerenchyma by regulating the generation of ethylene and ROS, and functioned in plants acclimation to waterlogging (hypoxia) stress.	ethylene	[62]
Under hypoxic stress	Arabidopsis	*AtLDH1*	*AtLDH1* encodes a lactate dehydrogenase, overexpression of *AtLDH1* increased the survival of roots and knockout *AtLDH1* impaired the growth of roots and shoots under hypoxic stress.		[98]
Under hypoxia stress	Arabidopsis	*AtRAP2.2*	*AtRAP2.2* encodes an ERF-VII type transcription factor, overexpression of *AtRAP2.2* increased the survival rate under hypoxia stress.	ethylene	[99]
Under anoxia condition	Arabidopsis	*AtHRE1*	Overexpression of *HRE1* in Arabidopsis enhanced its tolerance to hypoxic stress through regulating the expression of anaerobic genes and ethanol metabolism.	ethylene	[100]
Submergence	Arabidopsis	*AtRAP2.12*	*AtRAP2.12* encodes an ERF-VII type transcription factor and overexpressing *AtRAP2.12* in Arabidopsis showed increased survival rate under anoxia stress.	ethylene	[101]
Under hypoxia condition	Arabidopsis	*AtPCO1* and *AtPCO2*	Plant cysteine oxidase (PCO) makes the penultimate cysteine of ERF-VIIs oxidized and in turn, the expression of *PCO* is regulated by ERF-VIIs; overexpressing *AtPCO1* and *AtPCO2* in Arabidopsis became sensitive to submergence and the survival rate decreased significantly compared to wild type.	ethylene	[102]
Submergence	Arabidopsis	*AtRBOH I*	The expression of *AtRBOH I* (respiratory burst oxidase homolog I from Arabidopsis) is induced under hypoxic stress and loss function of *AtRBOH I* decreased the survival rate compared to wild type under submergence condition.	ethylene, auxin	[103]
Submergence	Rice	*Sub1A (SUB1A)*	*Sub1A* encodes an ERF-type transcription factor and can enhance submergence-tolerance of rice.	ethylene	[104]
Partial submergence	Deepwater rice	*SK1* and *SK2*	*SNORKEL1 (SK1)* and *SNORKE2* (*SK2*) are ethylene response factors and regulate the elongation of internodes through gibberellin under submergence stress.	gibberellin, ethylene	[31]
Waterlogging and submergence	Rice	*CIPK15* and *SnRK1A*	*CIPK15* encodes a calcineurin B-like (CBL)-interacting protein kinase that positively regulates the expression of *SnRK1A* (Snf1-related protein kinase 1), and functions in rice acclimation to flooding stress by affecting sugar and energy production.		[105,106]
Under anaerobic condition	Rice	*Amy3* subfamily genes	*Amy3* subfamily genes encode α-amylases and their expression can be induced in rice embryos by anaerobic conditions, which contributes rice to survive under submerged conditions.		[106]
Under anaerobic condition	Rice	*OsTPP7*	*OsTPP7* encodes a trehalose-6-phosphate (TP6) phosphatase and facilitates rice germination under anaerobic conditions by regulating local T6P/sucrose ratios.		[107]
Submergence	Rice	*OsEREBP1*	*OsEREBP1* encodes an ERF transcription factor and may enhance the submergence tolerance through regulating jasmonate and abscisic acid signals.	jasmonate, abscisic acid	[108]
Submergence	Rice	*LGF1*	LGF1 controlled the formation of leaf gas films and affected the synthesis of C30 primary alcohol, which ultimately improved submergence-tolerance of rice with increased underwater photosynthesis.		[109]
Submergence	Deepwater rice	*SD1*	*SD1* (*SEMIDWARF1*) encodes a gibberellin biosynthesis gene, it can promote internodes elongation by increasing synthesis of gibberellins and is regulated by a transcription factor OsEIL1a.	gibberellin, ethylene	[110]
Submergence	Rice	*OsARD1*	*OsARD1* encodes an acireductone dioxygenase (ARD) and mediates the biosynthesis of an initial substrate methionine in the process of ethylene synthesis. Overexpression of *OsARD1* increased the production of internal ethylene and submergence-tolerance of transgenic lines was improved simultaneously.	ethylene	[111]
Waterlogging and submergence	Deepwater rice	*ACE1* and *DEC1*	*ACE1* confers the intercalary meristematic cells with the division ability and therefore regulated the elongation of internodes together with GA; *DEC1* restricts internodes elongation.	gibberellin	[18]
Submergence	Maize	*Subtol6*	*Subtol6* is a major QTL that can explain 22% of the phenotypic differences in submergence tolerance within the recombinant inbred lines.		[112]
Waterlogging	Maize	*ZmEREB180*	*ZmEREB180* encodes an ERFV-II transcription factor from maize; overexpression of *ZmEREB180* increased the survival rate under waterlogging stress through promoting ARs formation and regulating antioxidant activities.	ethylene	[25]
Waterlogging	Wheat	*TaERFVII.1*	TaERFVII.1 belongs to ERF-VII family and functions in waterlogging-tolerance of wheat, overexpression of *TaERFVII.1* increased the survival rate under waterlogging stress.	ethylene	[17]
Waterlogging	Barley	*HvERF2.11*	The expression of *HvERF2.11* can be induced by waterlogging and mediated waterlogging-tolerance of plants through improving some antioxidant and ADH enzymes activities.	ethylene	[113]
Waterlogging	*Actinidia deliciosa*	*AdPDC1*	*AdPDC1* encodes a pyruvate decarboxylase which catalyzes the first step in ethanolic fermentation pathway and it may function in kiwifruit acclimation to waterlogging stress.	abscisic acid	[114]
Waterlogging	*Actinidia deliciosa*	*AdRAP2.3*	AdRAP2.3 is an ERF-VII transcription factor and may mediate waterlogging-resistance by regulating *PDC* and *ADH* genes.	ethylene	[115]
Waterlogging	*Chrysanthe-mum morifolium*	*CmSOS1*	*SOS1* encodes a Na^+^/H^+^ antiporter and may interact with CmRCD1 to mediate plant tolerance to waterlogging stress potentially.		[116]
Waterlogging	Cucumber	*CsARN6.1*	*CsARN6.1* encodes an AAA ATPase, transgenic lines of *CsARN6.1* increased the number of ARs via enhanced ATPase activity and further affected waterlogging tolerance.		[117]
Waterlogging	*Mentha arvensis*	*MaRAP2-4*	*MaRAP2-4* from *Mentha arvensis* encodes an ERF-I type transcription factor, overexpression of *MaRAP2-4* in Arabidopsis enhanced its tolerance to waterlogging and oxidative stress.	ethylene, jasmonic acid	[118]
Waterlogging	Petunia	*PhERF2*	PhERF2 may regulate the process of programmed cell death and alcoholic fermentation, on the base of which enhances waterlogging-tolerance of petunia.	ethylene	[119]
Submergence	Arabidopsis	*WRKY33*, *WRKY12*	WRKY33 regulates the expression of *RAP2.2* together with WRKY12, in turn, RAP2.2 exhibits a feedback regulation to *WRKY33*. These three genes mediate submergence-tolerance of Arabidopsis collectively.	ethylene	[19]
Submergence	Arabidopsis	*ANAC017*	*ANAC017* encodes a NAC transcription factor and mediates differential submergence stress of Arabidopsis in juvenile and adult stages.		[20]

## Supplementary Materials

Supplementary file 1 can be found at https://www.mdpi.com/1422-0067/22/3/1088/s1.
