# Supplementary material for: Plant Morphological, Physiological and Anatomical Adaption to Flooding Stress and the Underlying Molecular Mechanisms
Source: Int J Mol Sci. 2021 Jan 22;22(3):1088. doi: 10.3390/ijms22031088 (PMC7865476; doi:10.3390/ijms22031088)
Supplement: Supplementary file 1 [file ijms-22-01088-s001.pdf]

Supplemental file 1. The coding sequences of ERF-VII family genes in phylogenetic tree of Figure 3

AtHRE1
ATGTGCGGAGGAGCTGTAATTTCCGATTACATAGCGCCGGAGAAGATTGCG
AGATCATCTGGAAAGTCTTCCTGGAGAAGTAATGGCGTCTTTGACTGCTCA
ATCTACGATTTCGATGGAAATTTCGATGAATTAGAGTCCGATGAGCCATTTG
TCTTCTCCTCTACTCACAAACATCATGCTTCAGGCTCAGCATCAGATGGGAA
GAAGAAACAGAGCAGTCGGTACAAAGGAATCAGAAGAAGGCCTTGGGGA
AGATGGGCGGCTGAGATACGTGATCCAATCAAAGGAGTTCGAGTTTGGCTC
GGGACTTTCAACACAGCTGAAGAAGCTGCAAGAGCTTATGATCTTGAAGC
TAAGAGAATCCGTGGAGCCAAAGCTAAGCTCAATTTCCCTAACGAATCCTC
TGGAAAGAGGAAAGCCAAGGCTAAGACTGTGCAACAGGTAGAGGAGAAT
CATGAGGCTGATCTTGATGTGGCGGTGGTAAGCTCAGCGCCTAGTAGTAGC
TGTCTTGATTTCTTGTGGGAGGAGAATAATCCGGACACGCTTCTGATTGATA
CACAATGGCTCGAAGATATCATCATGGGCGATGCGAATAAGAAACATGAAC
CTAATGATAGTGAAGAAGCCAACAACGTTGATGCTTCTCTGCTTTCTGAAG
AGCTTCTTGCTTTTGAGAACCAGACCGAATATTTCTCGCAGATGCCTTTTAC
GGAGGGAAACTGTGATTCCTCAACGTCTCTGAGTAGTCTCTTTGATGGAGG
CAATGACATGGGTCTATGGTCCTGA
AtHRE2
ATGTGTGGGGGAGCTATCATTTCTGATTTCATCTGGTCGAAATCTGAGTCAG
AACCGAGTCAACTCGGCTCTGTTAGCAGCAGGAAGAAGCGTAAACCCGTC
TCAGTGAGTGAAGAAAGAGATGGGAAACGAGAGAGGAAGAATCTGTACA
GAGGGATAAGGCAGAGGCCATGGGGCAAATGGGCAGCGGAGATTCGTGA
CCCGAGCAAAGGTGTACGTGTCTGGCTTGGCACATTCAAAACCGCCGACG
AAGCTGCTCGAGCCTACGACGTTGCTGCCATCAAAATCCGTGGCCGGAAA
GCCAAACTGAATTTCCCAAACACTCAAGTAGAAGAAGAAGCCGATACTAA
ACCAGGGGGGAATCAAAATGAGCTGATTTCGGAAAACCAAGTAGAGAGCT
TATCGGAGGACCTGATGGCATTGGAGGATTACATGAGATTCTATCAGATTCC
GGTTGCCGACGACCAATCGGCGACCGATATTGGAAATTTATGGAGCTATCA
AGACTCCAATTAA
AtRAP2.2
ATGTGTGGAGGAGCTATAATCTCCGATTTCATACCTCCGCCGAGGTCCCTCC
GCGTCACTAACGAGTTTATCTGGCCGGATCTGAAAAACAAAGTGAAAGCTT
CAAAGAAGAGATCGAATAAGCGATCCGATTTCTTCGATCTTGACGATGATTT
CGAAGCTGATTTCCAAGGGTTTAAGGATGACTCGGCTTTTGACTGCGAAGA
CGATGATGATGTCTTCGTCAATGTTAAGCCTTTCGTCTTCACCGCAACTACT
AAGCCCGTAGCTTCCGCTTTCGTCTCCACTGGTATATATTTGGTAGGTTCAG
CATATGCCAAGAAAACTGTAGAGTCCGCTGAGCAAGCTGAGAAATCTTCTA
AGAGGAAGAGGAAGAATCAATACCGAGGGATTAGGCAGCGTCCTTGGGGA
AAATGGGCTGCGGAGATCCGTGATCCGAGAAAAGGCTCCCGAGAATGGCT

TGGAACATTCGACACTGCTGAGGAAGCAGCAAGAGCTTATGATGCTGCAG
CACGCAGAATCCGTGGCACGAAAGCTAAGGTGAATTTTCCCGAGGAGAAG
AACCCTAGCGTCGTATCCCAGAAACGTCCTAGTGCTAAGACTAATAATCTTC
AGAAATCAGTGGCTAAACCAAACAAAAGCGTAACTTTGGTTCAGCAGCCA
ACACATCTGAGTCAGCAGTACTGCAACAACTCCTTTGACAACTCTTTTGGT
GATATGAGTTTCATGGAAGAGAAGCCTCAGATGTACAACAATCAGTTTGGG
TTAACAAACTCGTTCGATGCTGGAGGTAACAATGGATACCAGTATTTCAGTT
CCGATCAGGGCAGTAACTCCTTCGACTGTTCTGAGTTCGGGTGGAGTGATC
ACGGCCCTAAAACACCCGAGATCTCTTCAATGCTTGTCAATAACAACGAAG
CATCATTTGTTGAAGAAACCAATGCAGCCAAGAAGCTCAAACCAAACTCT
GATGAGTCAGACGATCTGATGGCATACCTTGACAACGCCTTGTGGGACACC
CCACTAGAAGTGGAAGCCATGCTTGGCGCAGATGCTGGTGCTGTGACTCA
GGAAGAGGAAAACCCAGTGGAGCTATGGAGCTTAGATGAGATCAATTTCAT
GCTGGAAGGAGACTTTTGA

AtRAP2.3

ATGTGTGGCGGTGCTATTATTTCCGATTATGCCCCTCTCGTCACCAAGGCCA
AGGGCCGTAAACTCACGGCTGAGGAACTCTGGTCAGAGCTCGATGCTTCC
GCCGCCGACGACTTCTGGGGTTTCTATTCCACCTCCAAACTCCATCCCACC
AACCAAGTTAACGTGAAAGAGGAGGCAGTGAAGAAGGAGCAGGCAACAG
AGCCGGGGAAACGGAGGAAGAGGAAGAATGTTTATAGAGGGATACGTAAG
CGTCCATGGGGAAAATGGGCGGCTGAGATTCGAGATCCACGAAAAGGTGT
TAGAGTTTGGCTTGGTACGTTCAACACGGCGGAGGAAGCTGCCATGGCTTA
TGATGTTGCGGCCAAGCAGATCCGTGGTGATAAAGCCAAGCTCAACTTCCC
AGATCTGCACCATCCTCCTCCTCCTAATTATACTCCTCCGCCGTCATCGCCAC
GATCAACCGATCAGCCTCCGGCGAAGAAGGTCTGCGTTGTCTCTCAGAGT
GAGAGCGAGTTAAGTCAGCCGAGTTTCCCGGTGGAGTGTATAGGATTTGGA
AATGGGGACGAGTTTCAGAACCTGAGTTACGGATTTGAGCCGGATTATGAT
CTGAAACAGCAGATATCGAGCTTGGAATCGTTCCTTGAGCTGGACGGTAAC
ACGGCGGAGCAACCGAGTCAGCTTGATGAGTCCGTTTCCGAGGTGGATATG
TGGATGCTTGATGATGTCATTGCGTCGTATGAGTAA

AtRAP2.12

ATGTGTGGAGGAGCTATAATATCCGATTTCATTCCACCGCCGAGGTCTCGCC
GTGTTACTAGCGAGTTTATTTGGCCGGATCTGAAGAAGAATTTGAAAGGAT
CGAAGAAAAGCTCGAAGAATCGTTCGAATTTCTTCGATTTTGACGCTGAGT
TCGAAGCTGATTTCCAAGGTTTCAAAGATGATTCGTCTATCGATTGCGATGA
TGATTTCGACGTCGGTGATGTTTTCGCCGATGTGAAACCATTCGTTTTCACT
TCGACTCCAAAACCCGCCGTCTCCGCCGCTGCGGAAGGTTCAGTTTTTGGT
AAGAAAGTTACTGGCTTGGATGGGGACGCTGAGAAATCTGCAAATAGGAA
GAGGAAGAATCAGTACCGAGGGATTAGGCAACGTCCTTGGGGAAAATGGG
CTGCTGAGATACGTGATCCAAGGGAAGGTGCTAGAATCTGGCTTGGAACGT
TCAAGACAGCTGAGGAAGCTGCTAGAGCTTACGATGCTGCAGCGCGGAGA
ATCCGTGGATCTAAAGCTAAGGTGAATTTCCCTGAAGAAAACATGAAGGCT
AATTCTCAGAAACGCTCTGTGAAGGCTAATCTTCAGAAACCAGTGGCTAAA
CCTAACCCTAACCCAAGTCCAGCTTTGGTTCAGAACTCGAACATCTCCTTT

GAAAATATGTGTTTCATGGAGGAGAAACACCAAGTGAGCAACAACAACAA
CAACCAGTTTGGGATGACAAACTCCGTTGATGCTGGATGTAATGGGTATCA
GTATTTCAGCTCTGACCAGGGTAGTAATTCTTTCGATTGTTCGGAGTTTGGT
TGGAGCGATCAAGCTCCGATAACTCCCGACATCTCTTCTGCGGTTATCAAC
AACAACAACTCAGCTCTGTTCTTTGAGGAAGCCAATCCAGCTAAGAAGCT
CAAGTCTATGGATTTCGAGACACCTTACAACAACACTGAATGGGACGCTTC
ACTGGATTTCCTCAACGAAGATGCTGTAACGACTCAGGACAATGGTGCAAA
CCCTATGGACCTATGGAGTATTGATGAAATTCATTCCATGATTGGAGGAGTC
TTCTGA

Solyc06g063070.2

ATGTGTGGTGGTGCAATTATCTCCGATTTGGTACCTCCTAGCCGGATTTCTC
GCCGGTTAACCGCTGATTTTCTATGGGGTACATCCGATCTGAACAAGAAGA
AGAAGAACCCTAGTAATTACCACTCAAAGCCCTTGAGGTCTAAGTTTATTG
ACCTTGAAGATGAATTTGAAGCTGACTTTCAGCACTTCAAGGATAATTCTG
ATGATGATGATGATGTGAAGGCATTTGGCCCCAAATCCGTGAGATCTGGTG
ATTCAAACTGCGAAGCTGACAGATCCTCCAAGAGAAAGAGGAAGAATCAG
TACCGGGGGATCAGACAGCGTCCTTGGGGTAAGTGGGCAGCTGAAATACG
TGATCCAAGGAAAGGTATTCGAGTCTGGCTTGGTACTTTCAATTCAGCCGA
AGAGGCAGCCAGAGCTTATGATGCTGAGGCGCGAAGGATCAGAGGCAAGA
AAGCTAAGGTGAACTTTCCTGATGAAGCTCCAGTGTCTGTTTCAAGACGTG
CTATTAAGCAAAATCCCCAAAAGGCACTTCGTGAGGAAACCCTGAACACA
GTTCAGCCCAACATGACTTATATTAGTAACTTGGATGGTGGATCTGATGATT
CGTTCAGTTTTTTCGAAGAGAAACCAGCAACCAAGCAGTACGGCTTCGAG
AATGTGTCTTTTACTGCTGTAGATATGGGACTGGGCTCAGTTTCCCCTTCAG
CTGGTACAAATGTTTACTTCAGCTCTGATGAAGCAAGTAACACTTTTGACT
GCTCTGATTTCGGTTGGGCTGAACCGTGTGCAAGGACTCCAGAGATCTCAT
CTGTTCTGTCGGAAGTTCTGGAAACCAATGAGACTCATTTTGATGATGATTC
CAGACCAGAGAAAAAACTGAAGTCCTGTTCCAGCACTTCATTGACAGTTG
ACGGTAACACTGTGAACACGCTATCTGAAGAGCTATCGGCTTTTGAATCCC
AGATGAAGTTCTTGCAGATCCCATATCTCGAGGGAAATTGGGATGCATCGG
TTGATGCCTTCCTCAATACAAGTGCAATTCAGGATGGTGGAAACGCCATGG
ACCTTTGGTCCTTCGATGATGTACCTTCTTTAATGGGAGGTGCCTACTAA

Solyc12g049560.1

ATGTGTGGTGGTGCCATAATCTCCGATTGGATACCGCCGTCTCGATCTTCGA
GCCGACTCACCGCCGACCAGTTATGGGGTTGCGCCGATCTGCAAAACAAG
AAGAGGAACAAGAAGAAGAGGAATCCTTCCAATTATCACTCTAAGCGCTT
GAGATCTGAGAATGTAGACTTTGAAGCTGATTTTCAGGATTTTAAGGATTTT
TCTGATGATGAAGAAGCTTATAGTTTGGATATCAAACCATTTGCTTTCTCTG
CTTCTGAACTCTCTGGAACCTCTGCTGGATCCGAATCACTGATATCTGTTGA
TGCAAACAAGGAAGTTGAGAAATCTGCCAAGAGACAGAGGAAGAATCAG
TATAGGGGGATCAGAAAGCGTCCTTGGGGTAAGTGGGCAGCTGAAATACGT
GATCCACAGAAGGGGGTCCGAGTTTGGATTGGAACTTTTAATACTGCAGAA
GAAGCTGCCAGAGCTTATGATGCTGAAGCTCGGAGGATCAGAGGCAATAA
AGCTAAAGTAAACTTTCCAGATGAAGCTTCAGTGCCTGCCTCGAGGCAAG

CTGGTAAGGTGAATCCTCGGAAGGTTCTTTCTGATGAGAGCTCTAACCCAG
TTCCACCCAACACCATGCTTATGAACAACTTGAATAGTGGATATTGTGACAA
TGTGGGCCTGCTCGAAGAGAAAACAAAGACTCTGAATGGCTACGAAGCTT
TGTGTGTGACTCCTGTAGATACAGGACCTAACCCATATCCCCATCCAGCTGC
TGCTGGTGTTTACTTCAATTCTGACCAAGGAAGTAACTCTTTTGGCCCCTCA
GACTTTTGGGGAGAAACATGTTCAAGGACTCCAGACATATCATCTGTTCTG
TCAGCTGCTATAGAATGCGATGAAGCTCAATTTATTGAAGGTGTCGACCTGG
AGGAGAAACCAAAATCTTGTACCAACAATTTGGTGCCTAATAATGTGAACA
CTGAACACAAGCCACCTGAAGTATTTTCAACTTTTGAATCCCAGTTGAAGT
TCTATCAGACACCATACTCAGAAGGAAATATGGATGTACCAGTCGATGCCTT
CCTCGATGCTGATGCTACTCAGGGTGTTGAAAATGCTATGGACCTTTGGTCC
TTTGATGAGCTTTCTTCTTTAATGGGAGGCATCTGA

Solyc09g075420.2

ATGTGTGGTGGTGCAATTCTTGCTGATATCATTCCTCCTCGTGACCGCCGTT
TGTCATCCACCGACCTATGGCCGACTGATTTCTGGCCAATTTCCACCCAAAA
TGTTCCTCTCAACCCCAAACGAGCTCGACCCTCTACAGGTGGTGAGCAGAT
GAAGAAGAGGCAAAGGAAGAATCTTTACAGAGGGATAAGACAACGTCCAT
GGGGTAAATGGGCTGCTGAAATTCGTGACCCGAGAAAAGGGGTTAGGGTT
TGGTTAGGTACTTTCAACACTGCTGAAGAAGCTGCAAGAGCTTATGATAGA
GAAGCTCGTAAAATCAGGGGTAAGAAAGCTAAAGTTAATTTCCCCAATGAA
GATGACGACCATTACTGCTACAGTCATCCAGAGCCCCCTCCCTTGAACATT
GCTTGTGATACTACTGTTACTTACAATCAAGAATCAAATAACTGTTACCCCT
TTTACTCAATCGAGAACGTTGAACCTGTTATGGAATTTGCAAGTTATAATGG
AATTGAAGATGGAGGAGAGGAGATGGTGAAAAATTTGAATAACAGGGTTG
TAGAGGAAGAGGAGAAAACAGAGGATGAAGTGCAGATACTTTCTGATGAG
CTGATGGCTTATGAGTCATTGATGAAGTTCTATGAAATACCGTATGTTGACG
GGCAATCAGTGGCGGCGACGGTGAATCCAGCGGCGGAGACCGCCGTGGGC
GGTGGCTCGATGGAGCTTTGGAGTTTTGATGATGTTAGTCGTCTACAACCA
AGTTATAATGTAGTTTAA

Solyc01g065980.2

ATGTGTGGAGGTGCCATAATCTCCGATTATGATCCCGCCGGAAGCTTCTACC
GGAAACTTTCTGCTCGTGACCTCTGGGCTGAGCTGGACCCTATCTCCGACT
ACTGGTCCTCTTCTTCCTCATCCTCAACCGTCGGAAAACCTGATTCCGCTCT
GTCGCCGGTGACTCACTCCGTCGATAAGCCTAATAAATCAGATTCCGGCAA
AAAAGGTAATAAGACTGTGAAGGTTGAGAAGGAGAAGAGTAGTGGACCA
AGGCCAAGGAAGAACAAGTACAGAGGAATAAGACAGAGGCCATGGGGAA
AATGGGCTGCTGAGATTCGCGATCCACAGAAGGGTGTACGCGTTTGGCTTG
GTACATTCAACACAGCAGAAGATGCTGCTAGAGCCTATGATGAGGCTGCTA
AGCGCATTCGTGGTGATAAGGCTAAACTCAACTTTCCAGCCCCATCACCAC
CAGCTAAGCGACAGTGCACTAGCACTGTCGCTGCTGCTGATACACCACCAG
CACTACTCCTTGAGAGTTCTGACAACTCTCCTTTGATGAACTTTGGATATGA
TGTCCAGTATCAGAGCCAAACTCCCTACTACCCCATGGAAATGCCCATAGTT
AGTGAAGATTATGAACTGAAGGAACAGATTTCCAATTTGGAATCGTTCCTG
GAATTGGAGCCATCTGATCAATTTTCAGGGATCGTCGATTCTGATCCTCTTA

ATGTTTTTCTGATGGAGGACTTTGCTTCAACTCATCATCAGTTCTACTGA

Solyc03g123500.2

ATGTGTGGTGGTTCTATAATCTCCGATTACATAGACCCTAGCCGGACTTCTC
GCCGGCTCACCGCCGAGTTTCTATGGGGTCGTTTCGATCTCGGTAAGAAGC
AAAAAAATCCCAACAATTATCACTCTAAAGCTAAGCATTTGCGATCTGAAG
TTGTTGACGACTTTGAAGCCGATTTTCAGGACTTCAAAGAGTTATCCGATG
ATGAGGATGTTCAAGTCGATGTCAAGCCATTTGCCTTCTCTGCTTCCAAAC
ACTCTACTGGTTCCAAATCTTTGAAAACTGTTGATTCAGACAAGGATGCTG
CTGCTGATAAATCCTCTAAGAGAAAGAGGAAGAATCAATATAGAGGGATCA
GACAGAGACCTTGGGGTAAGTGGGCAGCTGAAATACGTGACCCAAGGAAA
GGGGTTCGGGTCTGGCTGGGAACCTTCAATACTGCAGAAGAAGCTGCCAA
AGCTTATGATATTGAGGCGAGGAGGATCAGAGGCAAGAAGGCTAAGGTAA
ACTTTCCTGATGAAGCTCCCGCCCCTGCATCAAGACACACTGTTAAGGTGA
ATCCTCAGAAGGTCCTTCCTGAGGAGAGCCTGTATTCACTTCAGTCCGACT
CAGCAATCATGAACAGCGTGGAGGATGACCATTATGATTCTTTTGGATTTTT
TGAAGAGAAACCCATGACAAAACAGTATGGATATGAGAATGGGAGCAGTG
CTTCTGCAGATACGGGATTTGGTTCGTTCGTCCCTTCAGCTGGCGGTGATAT
CTACTTCAACTCTGATGTAGGAAGCAACTCTTTTGAATGCTCTGATTTTGGT
TGGGGAGAGCCATGCTCCAGGACTCCAGAGATATCATCTGTTCTGTCAGCT
GCTATTGAATGTAATGAAGCTCAATTTGTTGAAGATGCCAATTCTCAGAAAA
AGTTGAAATCATGCACCAACAACCCCGTAGCTGATGATGGAAACACCGTTA
CTATGGTACCTGAAGAGCTTCCAGCTTTTGAACCTCAGATGAATTTCTTTCA
TCTCCCATATATGGAGGGAAATTGGGATGCATCAGGTGGTAACTTCCTCAAC
ACAAGTGCAACTCAAAATGGTGGTGAAAATGCTATGGACCTGTGGTCCTTT
GATGATGTTCCTTCTTTAATGGGAGGTATCTTTTAA

OsSUB1A-1

ATGTGTGGAGGAGAAGTGATCCCCGCCGACATGCCGGCGGCGCCGTTCAC
GCCACGCCACGGCGACGGCGAGACATGGGTTGACAGAAAGAGGAGGAAC
AAGAAGAAGAGGAAGCGCGGCGCCGACGAAGAATGGGAGGCCGCCTTCC
AGGAGTTCATGGCTGCTGACGACGACGACGACGGCGGCGGACTCGTGTTA
AGTAGTAAATCTTTGGTGTTGAGGTCACCAGGTGAAAATGATGCAGGCCGG
GGCGCCGCCGCCACCATGTCCATGCCGCTGGACCCCGTGACCGAGGAGGC
CGAGCCGGCGGTGGCTGAGAAGCCTCGCCGGCGCCGGCCGAGGCGGAGC
TACGAGTACCACGGCATCCGGCAGCGGCCGTGGGGGCGGTGGTCGTCGGA
GATCCGCGACCCCGTCAAGGGCGTCCGCCTCTGGCTCGGCACCTTCGACA
CCGCCGTCGAAGCCGCGCTCGCCTACGACGCCGAGGCCCGCCGCATCCAC
GGCTGGAAAGCCCGGACAAACTTCCCACCCGCCGATCTTTCTTCGCCGCCG
CCGCCGTCGCAGCCGCTCTGCTTCTTGCTCAACGACAACGGCCTCATCACA
ATCGGAGAAGCGCCGACCGACGACGCCGCGTCGACGTCGACGTCGACGAC
GGAGGCGTCCGGCGACGCGCGCATACAACTGGAGTGCTGCTCGGACGACG
TGATGGACAGCCTCCTCGCCGGCTACGACGTGGCCAGCGGCGACGACATAT
GGACATGGACATCTGGAGCCTCCTCCACCTCTGTTAACCAAGAGATCAAGA
CCCCATCGATCCACCAAAACATATCATATGCAGGGGAAGCCTGA

OsSUB1B-1

ATGTGTGGAGGAGCACTGATCCCGAACGACTACGGCGACAAGCCGCCGCC
GCCGCCGTCGGAGTCGTCGGAGTGGGACGCCACAACGAAGATGAAGAAG
AAGAAGAAGCGTGGCGGCGGCGGCGACGACGACTGGGAGGCCGCCTTCC
GGGAGTTCATCGCTGGCGACGTCGACGACGACGACGACGGCGTTTCCATG
TTCCCTTCTGGTGCAGGGACGATGGAGACGACGACAGAGGTGGCGGTGGT
GGAGAGGCCGCGGCGGCGGCGAAGGGTGAGGCGGAGCTACCCGTACCGC
GGCGTCCGGCAGCGGCCGTGGGGGCGGTGGGCGTCGGAGATCCGCGACCC
CGTCAAGGGCGCCCGCGTCTGGCTCGGCACCTTCGACACCGCCGCCGAGG
CCGCGCGCGCCTACGACGCCGAGGCGCGCCGCATCCACGGCCACAAGGCA
AGGACCAACTTCCCGCCCGACGAGCCTCCGCGGCCGGCGCCATCGCAGGC
GCCGTTCTGCTTCCTGCTCGACGACGACGACGACGGCGTGGCCCGTGGAA
ACAGCCCGGCGTCGTCGTCGGCGCCGGACAGCACCTCCGCTTGCACGACG
TCGTCGACGGTGGCGTCCGGCGAGCGAGGCGATGAGCTCATACTGCTGGA
GTGCTGCTCCGACGACGTGATGGACAGCCTCCTCGCCGGCTTCGACGTGTC
CAGCGAATCACGCAGTATTTTGGGAATGGTTAATTAG

OsSUB1C-1

ATGCGCCGCCGCGTCTCCTCCTCCCCCTCCTCCTCCTCCTCGTCGCCGG
CGAGGCATCACAAGGCGCGGCGCAGCAGGAGGAAGCTCGTCGCCGACGA
GGACTGGGAGGCCGCCTTCCGCGAGTTCCTCTCCCGCGACGACGACGACG
ACGACGACGACGACGACGGGCACCACGTCGTTGTTGCGCCGCTGATCCGT
AGTAGTAACAAGTGCGTCCACGGCCACGAGGTGGTGGCGTCGACGGTCGG
CGGTGGCGCAAGCGGCGGACGACGACGAGCCGACGACGACGACGGCGAG
CGGCGGCGGCGGCGGCGGAGGGAGAGGCGGAGCTACCCGTACCGCGGCA
TCCGGCAGCGGCCGTGGGGGAGGTGGGCGTCGGAGATCCGCGACCCCGTC
AAGGGCATCCGCGTCTGGCTCGGCACCTTCGACACCGCCGAGGGCGCCGC
GCGCGCCTACGACGACGAGGTTCGCCGCATCTACGGCGGCAACGCCAAGA
CCAACTTCCCCCCATCGCCGCCGCCGCCGGAGCAGCCAGCGGCCCCGGTA
GCGGCGGAGAGGAGCCCCTCGACGACGACGACGACGACGCCGTCGGCGG
AGGACTCCGGCGACTCGCGCATACTCATCGAGTGCTGCTCCGACGACCTGA
TGGACAGCCTCCTCGCCGCCTTCGACATGACCACCGGCGACATGCGCTTCT
GGAGCTAA

AdRAP2.3

ATGTGCGGCGGTTCAATCCTCGACGAATTCATCCCTCGCAACGGTAACCAC
CGCGTCTCCGCCTCCCAACTCTGGCCCAACTCCCCCTTCGTCACCAAATTC
AAACCCCCACAAGATCAAAACGACGGTGATGAGCGTGTTGAAAAGAAGG
CCAAGAGACAGCGCAAGAACCTGTACAGGGGAATTAGGCAGCGTCCGTGG
GGAAAATGGGCAGCGGAGATCCGAGATCCGAGAAAAGGTGTGAGGGTTTG
GATCGGTACCTTCAACACGGCCGAAGAGGCCGCCAGAGCCTACGACAGGG
AAGCTCGCAAGATCCGAGGCAACAAAGCCAAGGTTAATTTTCCCAACGAA
GACGACCATTCCATTCAATTTACTCCACAAACCCATCATTTACCCACCGCTA
TGAGTCATCCCAACGGAGGGTTTAGTGGCAATCTGAACCAGTTTGGGGCAT
ACAGCTCTAATGGGTTCAATAGCGTCCCCTGTTCCGACCCTGTTTCGGTTCT
TCACTTTGAAGAAATTTCTGGGTCTGGTTTAGAAAGTTCTTACTCTTCGATT
GATTTCAAATTAGAGGTGAAGGAAGAGAGAGAGAAGCAAGAGGAGAGAG

GAAACAGGAAGGAAGCGGCGGTGATGGAAGTGGAGGAAGCAGCAGGGG
AAGAGAGCGAAGTGGAGAAGCTGTCGGAGGAGTTGATGGCCTACGAGTCC
GTCATGAAATTCTATCAGATTCCATATCTCGACGGCCAATCGACGAATGCTC
CGCCAGCGGAGAACGACGTCATCGGCTGTGGTGCTGTGGAACTATGGAGC
TTCGACGATCTTACCCCTACGGTGGCCTGA

PhERF2

ATGTGTGGTGGTGCTATAATTTCCGATTACATACCTCCGAACCGGACTTCTC
GCCGGTTAACCGCTGAGTTACTATGGGGTCGTTCCGATCTGAGTAAAAAT
CAAAAAATCCAAGTAATTATCATTCAAAGCCTTTGAGATCTCAAATAGTTGA
CCTTGACGATGACTTCGAGGCTGATTTTCAAGAGTTTAAAGATTTTTCAGAT
GATGAAGATGTTAAACCATTTGCTTTTTCTGCTCCCAAACAGTCCACTGGCT
CCAAATCTGTAAAATCTGCTGATTCAGAGAAGGATGCTGATAGTTCCTCTAA
GAGAAAGAGGAAGAATCAGTATAGGGGGATCAGACAGCGACCTTGGGGTA
AGTGGGCAGCTGAAATACGTGACCCAAGTAAAGGTGTTCGAGTCTGGCTT
GGAACCTTCAATACTGCAGAAGAAGCTGCCAGAGCTTATGATGTTGAGGCT
AGAAGGATCAGAGGCAATAAAGCTAAGGTGAATTTCCCTGATGAAGCGCC
AGTGCCATCTTCAAGACGCGCTGTTAAGGTGAATCCTCAGAAGGTCCTTCC
TAGCCTGGACTCTGTTCAGCCAGACACTACTGTGATGAACAACCTGAGGA
ATGGCTATTATGATTCTTTGGGATTTCTTGAAGAGAAACCTGTGGCAAAGCA
GTATGGATATGAGGATGGGGGCAGTACTTCTGTAGATATAGGATTCGACTCA
TTTGCGCCTTCAGCTGGTGCTGATATTTACTTCAACTCCGATGTGGGAAGCA
ACTCTTTCGACTGCTCTGACTTTGGTTGGGGAGAGCCGTGCACCAGGACTC
CAGAGATATCATCTGTTCTTTCAGCTGCTATAGAAAGTAATGAAGCTCAATT
TGTTGAAGATGCCCGTCCAGAGAAAAAACTGAAATCAGACCCCAACAATC
CAGTAGCTGATGATGGAAACACTGTGAACGAGCTATCTGAAGAGCTTTCAG
CATTTGAATCTGAGATGAAGTTTCTTCAGATACCTTATCTGGAGGGGAATTG
GGATGCATCAGCTGATACCTTCCTCAACACAAGTGCAACTCAGGATGGTGA
AAATGCTATGGACCTCTGGTCATTTGATGATGTTCCTTCTTTGATGGGAGGT
GTCTACTAA

ZmEREB180

ATGTGCGGGGGAGCGATCCTTGCCGAACTCATCCCAGCACGGGTGCACCG
GCCGCTGACCGCCGCCACGCTCTGGGCGGCGGCCCTGAGTGGGACCACTA
CCGTCGGCAAGTGGAAGGCCGATGCAGCCGCCGCTACCGACGACGACGAC
GACGAAGAGTTCGAGGCCGAGTTCCAGCTTTTCGACGACGATGACGAGTG
CGAGGCCGAGTTCCAGCTTCTCGACGACCACCAACCATCTCCCGCAGCTTC
GCCTGAGGCCAGCGGCTGCAAGCGGAAGCCTGCCCTTGCTCCTCCTGCTG
GTGCCCCCGCTTCCACGGATCCGGCGACCCCGTGCTCCAGGAAGTACAGG
GGCGTCCGGTACCGCCGGTCAGGCAGGTGGGCCGCGGAGATCAGGGACCC
GCGGCAGGGGCGCCGCGCCTGGCTCGGCACGTACCGCACCGCCGAGGAG
GCCGCCCTGGCGTACGACCGCGAGGCCCGCCGGATCCGCGGGAAGAGCGC
GCGGCTCAACTTCCCGCTGCTCATCCCGCACGAGGGTCCCGGCCGTCACGC
GCGCACGCCGGTGGCCATCGACCTCAACTTGCCGCCCGTCTCCGACGGTCT
CGGCGTCCCGGCTCCGGCTGGCGTCGGCGACGACGGCACGGCGAATGCCG
ACGGAGATGCAGATATGGCCAGTACTAAGACTCAGAGCACGCTAGCGCGG

GTCAAGGAACTGATCGCGCAGGGACCTCACGACGAGCGGCTGGCGGCGA
GGATCGTGCCTGAGCTGATGATGCACGGGAGCAGGGACGAGGCTGCGGCG
TTGATCGCCGAGTTCAGCCGTCAGATGGAGGAGATCGCTGCGTTGCGGAG
GGACCTTGAGACACGCGAGAGGCAGCTTGTTCAGCTGGTTTCTCTAGTACT
TCGTTGA

HvERF2.11

ATGTGCGGCGGAGCCATCCTCGCGGGATTCATCCCGCCGTCGGCGGCCGCG
GCGGCGGCCAAGGCAGCGGCGACGGCCAAGAAGAAGCAGCAGCAGCGCA
GCGTGACGGCAGACTCGCTCTGGACGGGCCTGCGGAAAAAGGCGGACGA
GGAGGACTTCGAGGCCGACTTCCGCGACTTCGAGCGGGACTCCAGCGAGG
AGGAGGACGACGAGGTCGAGGAGGTCCCCCCTCCGCCGGCGCCGGCGAC
GGCCGGGTTCGCCTTCGCCGCCGCGGCCGAGGTCGCGCTCAGGGCCCCTG
CCCGCCGAGATGCTGCTGTTCAACATGATGGACCTGCTGCCAAACAAGTAA
AGCGCGTTCGGAAGAATCAGTACAGAGGGATCCGCCAGCGTCCCTGGGGG
AAATGGGCAGCTGAAATCCGTGACCCTAGCAAGGGTGTCCGGGTTTGGCT
CGGGACATACGACACTGCTGAGGAGGCAGCCAGGGCATATGATGCTGAGG
CCCGCAAGATCCGTGGCAAGAAAGCCAAGGTCAATTTTCCTGAGGATGCT
CCGACTGTTCAGAAGTCTACCCTGAAGCCAACTGCTGCTAAATCAGCAAA
GCTAGCTCCACCTCCGAAGGCCTGCGAGGATCAGCCTTTCAATCATCTGAG
CAGAGGAGACAATGATTTGTTCGCGATGTTTGCCTTCAGTGACAAGAAGGT
TCCTGCAAAGCCAACTGACAGTGTGGATTCCCTTCTTCCAGTGAAACACCT
TGCCCCCACCGAGGCATTCGGAATGAACATGCTCTCTGACCAGAGCAGCA
ATTCATTTGGCTCCACTGACTTTGGGTGGGACGACGAGGCCATGACCCCGG
ACTACACGTCCGTCTTCGTACCGAGTGCTGCTGCCATGCCGGCGTACGGCG
AGCCCGCTTACCTGCAAGGCGGAGCTCCAAAGAGAATGAGGAACAACTTT
GGCGTAGCTGTGCTGCCTCAGGGAAATGGTGCACAAGACATCCCTGCTTTT
GACAATGAGGTGAAGTACTCGTTGCCCTACGTTGAGAGCAGCTCGGACGG
ATCTATGGACAACCTTTTGCTGAATGGTGCGATGCAGGATGGGGCAAGCAG
TGGGGATCTCTGGAGCCTCGATGAGCTGTTCATGGCAGCTGGTGGTTATTG
A

ZmEREB179

ATGTGCGGGGGAGCGATCCTTGCCGAACTCATCCCAGCACGGGTGCACCG
GCCGCTGACCGCCGCCACGCTCTGGGCGGCGGCCCTGAGTGGGACCACTA
CCGTCGGCAAGTGGAAGGCCGATGCAGCCGCCGCTACCGACGACGACGAC
GACGAAGAGTTCGAGGCCGAGTTCCAGCTTTTCGACGACGATGACGAGTG
CGAGGCCGAGTTCCAGCTTCTCGACGACCACCAACCATCTCCCGCAGCTTC
GCCTGAGGCCAGCGGCTGCAAGCGGAAGCCTGCCCTTGCTCCTCCTGCTG
GTGCCCCCGCTTCCACGGATCCGGCGACCCCGTGCTCCAGGAAGTACAGG
GGCGTCCGGTACCGCCGGTCAGGCAGGTGGGCCGCGGAGATCAGGGACCC
GCGGCAGGGGCGCCGCGCCTGGCTCGGCACGTACCGCACCGCCGAGGAG
GCCGCCCTGGCGTACGACCGCGAGGCCCGCCGGATCCGCGGGAAGAGCGC
GCGGCTCAACTTCCCGCTGCTCATCCCGCACGAGGGTCCCGGCCGTCACGC
GCGCACGCCGGTGGCCATCGACCTCAACTTGCCGCCCGTCTCCGACGGTCT
CGGCGTCCCGGCTCCGGCTGGCGTCGGCGACGACGGCACGGCGAATGCCG

ACGGAGATGCAGATATGGCCAGTACTAAGACTCAGAGCACGCTAGCGCGG
GTCAAGGAACTGATCGCGCAGGGACCTCACGACGAGCGGCTGGCGGCGA
GGATCGTGCCTGAGCTGATGATGCACGGGAGCAGGGACGAGGCTGCGGCG
TTGATCGCCGAGTTCAGCCGTCAGATGGAGGAGATCGCTGCGTTGCGGAG
GGACCTTGAGACACGCGAGAGGCAGCTTGTTCAGCTGGTTTCTCTAGTACT
TCGTTGA

ZmEREB181

ATGTGCGGCGGTGCGATCCTCGCCGAGCTCATACCCAGCGCGCCGGCGCGG
CGCGGCGTCACGCCAGGCCACGGTAGCGGCGGCAAGGGTCGGACTGCGG
CCAACGACGACGACGACTTCGAGGCCGCGTTCCGTGACTTCGACGAGGAC
TCCGAGGAAGAGCCGGTGGGCGCGATTACGAAGAAGGCGTTCGGGTTCGC
CGGGACCCGGCGCAGGCGGCCCAGCAGCCCGTACTACGGCGTGCGGCGCC
GGCCGTGGGGCAAGTGGGCGGCCGAGGTGCGCGACCCCGTCCGGGGCGT
CCGCGTCTGGCTCGGCACCTTCGCCACCGCCGAGGCCGCGGCGCGCGCCT
ACGACCACGCCGCGCGCGACCTCCGCGGCGCCACCGCCAGGCTCAACTTC
CCGTCCAACTCCAAGCGCCGCTCCGCCGTTGCCAAGGCGGCGCCGCGCGT
GGACGAGGACGAGGACGAGGACGAGAACGGAGGAGGCGGCGCCCTGCCC
GACAGGTCGTGCCAGGGCATGTCCGCGGTCGGCTTGTGCGTCGAACTCGG
CGGCGCCTCGAAGCGCGCCCGGACAGAGCCCCAGGAAGAAGCCCAGGGA
AGGGAGAAGAAGGTGGCGCCTGCGCTGGCGCCGTCCGACGATTCCGCCGA
TACGCTGCTGATGGACGCTTTCATGTTCGGCGACCCGTTCAGCTTCTTCGAC
GGCGGCTTGTGCTACGAGCCTGCCGTGACGATGGACTGCCAGTTGTTGGGC
GGCGACGCCGTGTTTTGCGACGACAGCGTGGGACTCTGGAGCTTCGACGA
CAGTGTTTGCTTCTAG

ZmEREB182

ATGTGTGGTGGTGCGATCCTCGCCGAGCTCATCCCGACCACGCCGGCGCGC
CGCGGCGGCGCGGCGTGGCCCGCCGGCAAGTGCAAGCGGCGGAAGGCGA
ACGACTTCGAGGCCGCGTTCCGGGAGTTCGACGAGGACTCCGAGGAGGA
GGAGGAGGACGAGGGGGTGGTGGAGAGCAAGCCCGCCTTCGTCGTCCGC
GCCTCGTCCGCGGCGACGCAGCCCAGGGCGCGCCAGGAGCAGCGGAGCC
GGCGCCGGGCGCCCGACCAGCAGCAGTACAGGGGCGTCCGGCGCCGCCC
GTGGGGCAAGTGGGCCGCCGAGATCCGCGACCCCGTCAAGGGCGTCCGCG
TCTGGCTCGGCACCTTCCCCTCCGCCGAGGCCGCCGCGCGCGCCTACGACC
ACGCCGCGCGCGGCATCCGCGGGCCCAGGGCCAAGCTCAACTTCCCCTCC
TCCTCCTCCTCCGCCGCCGGGGCCCCCGCCAGCCGCAAGCGCGCCCGCAC
CGCCGACGCCGACGCCGTCATCGACCTCGAGGAACAAGAGCAGCGCCCGG
CCGCGGCGGCCCTGAAGCACGAGGCCGCCGAGTGCTCCGGATCCGACGCG
CTCCCGGACTTCTCGTGGCAGGGCGTGTCGGCGGCCTACGACGAGCCCCC
CGCGCCGCGCCCGGCCTTCGACTTCGAGGCGCGGCCGGAGAGCGCCGCGG
ACGAGGCGTCGCCCCGGGCCTCCGACTCCGAGTCGGACGCGCTGTTCGAC
TCGCTCCTCTTCGGCGACCAGTTCGCCTTCTTCGACGCCGGCGCGTACGAG
TCGCTGGACAGCCTGTTCAGCGCCGACGCCGTGCAGAGCAACGCCGCCGC
CGCGGCGGACCAGGACATGCCGCTGTGGAGCTTCGACGACGGCTGCCTCG
TCGAGGACAACCTCTCGTTTTAA

ZmEREB172

ATGACCAACCTCTCCGCCATGGCAGCGTACCAATCCTACGTGATCCGATTCG
ACGGCCACTTCGACGAGCCGTCGCCGAGCTCCGCGGGCGCCGAGCCGCCG
GGCCCGCAGCCGCCGTTCGCAGGGAGGATGATCTCGCTCGAGCGGGAGCA
CCAAGTCATTGTCGCCGCCCTGCTGCACGTCGTCTCCGGGTACGCCACGCC
GCCGCCGCAGGTCTTCACTCCGCCGCCGGCAGCGGCGCGCTGCCAGGTGT
GCGGGATGGAGCGGTGCCTCGGCTGCGAGTTCTTCGCCGCCGGGGAGGGC
GCATTGGGCGGCGGCGCGGAGAAGGCGGCCGCGACAGGAGGCGCGGCGG
CGGGGCAGAGGAGGCGGAGGAAGAAGAACAAGTACCGCGGCGTGCG
GCAGCGGCCGTGGGGGAAGTGGGCGGCGGAGATCCGCGACCCGCGCCGC
GCGGTGCGCAAGTGGCTGGGCACGTTCGACACCGCCGAGGAGGCCGCCA
GGGCCTACGACCAGGCCGCCATCGAGTTCCGTGGCCCGCGCGCCAAGCTC
AACTTCCCGTTCCCGGAGCAGCCGACGGGGCACGACGAGCCCATCAGCAG
CAACGGCGAAGCGAGCGCGGCCGCCAGGTCGTCGGACACCACGCCGTCG
CCGTCTCTCTGCAGCGCGGACGCCGAGGAGCGCGGGCAGCCAGAGTGGCC
GAGCGCCGGGCAGGAGGCAGGGGAGCAACTGCTCTGGGAAGGCCTGCAG
GACCTGATGAGGCTGGACGAAGGCGAGCCCTGGCTCCATCCAGCCTCAAG
CGCTTGGAATTGA

ZmEREB167

ATGCCATCACCACCCACAAAAAGACATAAGCCAGTTGTAGGGAAGGGCCA
AGGGAAACTTCAAGATAGTATGGCGAAGAACTATCGAGAGCGCCGCTTAA
AGAGGGTGATGAAGATAGATGAGGGAGAGGACGACTTTGAAGCTGATTAC
CAAGCATTCTCTAAGAAGTACGATGAGAAGGATGAGTGCCACTATAAGTTC
ATGGCTTTATTACCATTCAAGAATGGTCTTCCTAATGTCTCACCGCCAACAA
AAGAGAAAGTTGGACTTCATTTTGCCATGAAAGATGTTGTGATTAGTCAAA
AACCAACCACCACCAATGAAGGTATCATTGTAAGGCCGAAGTGCAAGCGA
AAGACCCCATATAGGGGCATCCGCCGACGCCCTTGGGGAAAATGGGCTGC
AGAAATCCGCGATCCTACAAAAGGTGTTCGTGTTTGGTTAGGCACGTATAA
AACACCAGAAGATGCAGCTAGAGCATATGACGCTGAGGCCCGCAAGATTA
GGGGCAATAAAGCAAAGGTGAATTTCCCTAATGAACCACCAAACATGGTG
AGCAATTCACCAAAACTAATTGTTACTGCAATGACTACAATGGCTATCCCAG
CAGACAAGTTGAATGCCAATGAATTGATGTGCCATAAAAACTACTCAAATG
AGGATTTTTTCTCAATGGTGAACTTTAGTGGAAACAATGGTAACTTTATTTC
TACTGCGGCCTTTGGCTTACAATGTATCAAAAAGCCTCGTGTTCCTTATGGC
ATCCCAAGGATTGGTGAGTGTTCAAATCAAAATAGAATGACATATGGTTTAA
GTAATGGATTGGGCAATGAAGCTAGTAGGAACCTGAATGGATGCTCTTTCTT
ACCACAAGCTGGCATGCCAATTTTCATTCAACCTACTTTTGATGGCCCTTCA
AGGATGATAGGAAGAAATGATGGCGTGATTGTTCCTACTTTGACTAATGCA
ACACCCATGGCTGGTGTTGATGCTGCGAAAAAGATGAATCAAGAGCCTATT
TTCCAAGAGATGGAAAGTGAAGATATTCCTTCTATTTTGCAAGGTGATGTCA
GTGAGGACGTGGCAGCTGAGATTAGTATGTGGGAGTTTTATGACAACCTAT
TAGATAATAAGGAAAATTGA

ZmEREB211

ATGACCAAGAAGCTCATCTCCATCTCCACCATGGCCGGGAAGCAAGGTTGC

AAGGAGCAGTCCAATGATCGGAGGATCCAGGCTTCGATCCAAGGAGACGC
CAAGAGCGTGGTGGTGGGGTTCGGCGGCAGGCTGGTCACCCGTGAGCAG
GAGCAGGAGGACGCCATCATCGTCGCGGCGCTGCGGCACGTGGTGTCCGG
GTACAGCACGCCGCCGCCGGAGGTCGTCGTCACGGTGGCGGGCGGGGAG
GCGTGCGGGACCTGCGGCATCGACGGGTGCCTCGGCTGCGACTTCTTCGG
GGCGCCGGAGCTGGCGCAGGAGGCAGTGTCCTGCGGCACAGGGCAGGTG
GCGACGACGGCGTCGGTCGGGGGCGGGGCAGCGGCGGCGGCGCTGAGGC
CCCGGCGGCGTAGGAACAGGAACATGTACCGCGGCGTGCGGCAGCGGCCG
TGGGGCAAGTGGGCGGCGGAGATCCGCGACCCGCGGCGCGCGGCGCGCG
TGTGGCTGGGCACGTTCGACACCGCGGAGGAGGCTGCCAGGGCCTACGAC
TGCGCCGCGGTCGAGTTCCGCGGCCCCCGCGCGAAGCTCAACTTCCCGGG
CCACGAGGCGCTGTTCCAGGGCCACGGCGACACCGCGGCGGCGAACACC
GAGACACAGACGCCGTCGCCGTCGCCGTCGCCGGGAGAGTGGCAGCTGG
GCGGCGGAGCAGGAGACGAGCTGTGGGAAGGTCTACAGGACCTGATGAA
GCAGGACGAGGTGGACCGCTGGCTCGCGCCGGTTTTCGGCGCCGCGTCTA
GTTTTTGA

ZmEREB202

ATGTGCGGCGGCGCCATCCTCTCGGGTTTCATCCCGCCGTCCGGGGTGCCG
GCGGCGGCAGCGGCGGCCAAGAAGAAACAGCAGCGGCGTGCGACGGCGG
ACCTGCTGTGGCCGGGGCCCGGCAAGAAGGGAGCGCCCCGGGAGGAGGA
CTTCGAGGCCGACTTCCGCGAGTTCGAGCGCGGCCTCGGAGAGGATGACG
ACGTGGACGGGGCCGGCGACGAGGTCCAGGAGCTTCCTCTGCCGGAGCCG
GCGAGGCTCGCCTTCGCCGCCGCGGTTGGGGCGCCGCGCACTGCAGTTGA
CGGCGTGATGACTCCAAAGGATGGTGAAGGAGACATGCCTACTACCGCTAC
CAACTCAGCAGCAAATAAGCGCAGGCGGAAGAACCAGTACAGGGGAATCC
GGCGGCGGCCCTGGGGCAAGTGGGCGGCCGAGATCAGAGACCCCAGCAA
GGGCGTGCGCGTCTGGCTCGGCACATACAGCACCGCCGAGGAGGCGGCCA
GGGCGTACGACGCCGAGGCTCGCAGGATACGCGGGAAGAAAGCCAAGGT
CAATTTCCGGGACGAAGCGGCGGCAGCAGCAGGTGCTCAGAAGGCACCA
GCCGCAACTACTCCAACTGCTTGTGGCAACGCAGCGAGGCTAGGTCCTCC
ACCTCCACCGCCGAAGTTCCGTGCTGACGAGGTTTTCGGGAACATGAACG
GTGGCACTGGCAGCAACGATCTGTTCGCGATTATGTTCGCGTTCAGTGACA
GTAGTAGTAAGGTCGTTCGTGTGGAGCCAGGTGAGGGCGCCGCCGGTTTC
CTCCCCGCAGATCTGTTGCCCGGCAGTAAGAGGTCTGCGGCTAACATGCTG
CTGCTCTCCGACCAGAGCAGCGACTCGTATGGCTCTTGTGACTTGGGGTGG
GAGTGGGACTGGGACGATGACACCATGACCTCAGACTACGCCTCAGTCTTT
GCTCCAGCTGCTCCCAGTAACGTTGTGCCAGCATGGTACACGCAAGGTGG
ACCGGTGTCGAAGAGAACGAGGAGCAGCTACGGCTACGGTGCGGCCATGC
CGGGCGGCTTTGACCCTGAGACGAACTACCAGTACCAGCCGTTGCCTTATG
TCGTCGAGAGCAGCCCGTCAGACGGCGCGTCGACGGACGACATGGACTGC
CTTCAAATGATGCGGGCTGGTGATGTTCCACAGGATGGGGCGAGCAGTGGT
GGTGGTGGCGGCGACGGCGGCGATATCTGGAGCCTTGACGAGCTGCTCAT
GGCCGCTGGCGCTTACTGA

ZmEREB193

ATGTGCGGCGGTGCAATCATCTTCGACTACATCCCGGCGCGGCGCCGGGTG
TCCGCCGCTGACTTCTGGCCTGACTCCGAAGCCGACGCCGAGGACTCCGA
CTCGCACGCCCCTGACCCTGAGAGAGCTCCGCTGCCGACTGTCCCGTCCC
GTGCAGTGCCGCGCCCGCGCGGGAAGGCGAAGCGTGGGCGCAAGAACCA
GTACCGCGGCATCCGGCAGCGGCCGTGGGGCAAGTGGGCGGCAGAGATCC
GCGACCCCGTGAAGGGCGTGCGCGTCTGGCTCGGCACCTACCCCACCGCC
GAGGCCGCCGCGCGCGCGTACGACCGCGCTGCCAGGCGCATCAGGGGCGC
CAAAGCCAAGGTCAACTTCCCCAACGACGCCTCCTCCTCCAGCGTCACCG
CGCCGACGGCGGCATCTGGGGTGGCGGCACAGGCCCCGGCCGTTTTTCTC
CCGGCGCCCAAGGTAGAGGCGCGCGCACAGGTGTCCGACGAGGTCAAGG
AGCTGTCCGAGGAGCTAATGGCCTACGAGAACTACATGAACTTCCTCGGCG
TCCCGTACATGGAGGGCGGGAATGCAGCTGTAAGTGCCACCACCGCTGCC
GCTGCCGCTGCCGCCGTCCCCGAAGAAGCGCAGGTGCCGGCGCCAGCGCC
AGCCGGGCTGTGGAGCTTCGAGGACTACTACTACCCGCCGTCACTGTCGCT
CTTCACTGAATAA

ZmEREB14

ATGTGCGGCGGCGCGATCCTTGCCGAGCTCCGCGAGCCGGCGCCGCGCCG
GCTCACGGAGCGGGACATCTGGCAGCAGAAGAAGAAGCCCAAGAGGGGC
GGCGCCGGCGGGAGGCGCTCGTTCGCGGCGGAAGACGATGAGGACTTCG
AGGCCGACTTCGAGGACTTTGAAGCCGACTCCGGTGATTCGGATTTGGAG
CTCGGGGAAGGGGCTGACGACGACGTCATCGAGATCAAGCCCTTCGCCGC
CAAGAGTACTTTCTCCAGAGATGGCTTAAGCACCATGACTACTGCTGGTTAT
GATGCCCCTGCAGCAAGGTTGGCCAAAAGGAAGAGGAAGAATCAATACAG
GGGTATCCGCCAGCGCCCTTGGGGTAAGTGGGCTGCTGAGATCAGAGATCC
CCAGAAGGGCGTTCGTGTTTGGCTTGGTACTTTCAATAGTCCCGAGGAAGC
TGCAAGAGCTTATGATGCTGAAGCGCGCAGGATTCGTGGCAAGAAGGCCA
AGGTTAACTTTCCTGATGCACCAGCAGTTGGTCAGAAGTGCCGTTCTAGTT
CAGCTTCTGCTAAAGCACTCAAGTCATGTGTTGAACAGAAGCCAATTGTCA
AAACAGATATGAACATCCTTGCCAACACAAATGCACCCTTCTACCAATCTG
TTAACTACGCATCCAACAATCCATTTGTTCCAGCAATGAACTCTACTGTTTC
TTTTGAGGATCCTATCATGAATCTGCACTCTGACCAGGGAAGTAACTCCCTT
GGCTGCTCAGACTTGGGCTGGGAGAATGATACTAAGACACCAGACATCAC
ATCCATTGCTCCCATTCCCACTATTGCTGAAGGCGATGAGTCTGTATTTGTC
AACTCCAATTCAAACAGCTCGATGGTGCCTCCTGTCCTGGAGAACAATGCT
GTTGATCTCACTGATGGGCTGACAGATTTGGAATCCTATATGAGGTTTCTTAT
GGATGGCGGTGCAAGTGATTCAATTGATAGCCTTCTGAACCTTGATGGATC
ACAGGATCTTGGTAGCAATATGGACCTCTGGACCTTCGATGACATGCCCATC
GCTGGCGATTTCTTCTGA

ZmEREB7

ATGACGTCGAGGCTGGAGAGCGGCGGGTTCCAGCTCCCGAACACCGAGCA
GGAGAACGCACTCTTGCTCCGCGCGCTCATCTCCGTCGTGTCCGGTGACAC
CGCCGCCGCGTCGTTGGTCCCGGAGGCGGCCGCCGCAGAGGCCCCTGCCG
CCGCGGCGTCAGCGTGCGGGTGCCCCGGCGGCTGCGACCTCGCCGCCGCG
TCGAGCAGCGACAGCGATGGCGCGGAGTGCTCCGCGAGCGGCGGAGGCG

CGGGCAAGCGGAGGCGGAGGCGGAGCAGGGCGAGCAGTTACATGGGCGT
GCGGCGGCGGCCGTGGGGCAAGTGGGCGGCGGAGATCCGCGACCCGCGC
CGCGCCGCGCGCAAGTGGCTCGGCACGTTCGACACCGCCGAGGACGCCGC
CCGCGCCTACGACGCCGCCGCGGTCGAGCTCCGGGGCCGCCGCGCCAAGC
TCAACTTCCCGGACGCCGCCGCCGCCGCGGCGGCGCGGGATGTGCAGCCG
CGCCGCCCGTTGCCCGGTCAGAGCCTCCGCGAGAACTGCGGGTCCAACGC
CGCGTCGCCGCTGCACGTGGCGGTGGCGCGGGCGCCGACCACGCTGCAGG
GAACAGGGCCGGCGCCGCCCAAGGACCAGGACATCTGGGACGGCTTGAA
CGAGATCATGACGATGGATGACGGCAGCTTCTGGTCCATGCCGTGA

ZmEREB90

ATGTGCGGCGGCGCGATCCTTGCCAACCTTCGCGAGCCGGCGCCGCGCCG
GCTCACAGAGCGGGACATCTGGCAGCAGAAGAAGAAGCTCAAGAGGGGC
GGCGGCGGCGGGAGGCGCTCGTTCGCGGCGGAGGACGATGAGGACTTCG
AGGCCGACTTTGAGGTCTTCGAGGCAGACTCCAGTGATTCAGATTTGGAGC
TCAGGGAGGGGACTGACGACGACGTCGTCGAGATCAAGCCCTTCACTGCC
AAGAGGACTTTCTCCAGCGATGGCTTAAGCACCATGACTAGTGCTGTAGCA
AGGTCAGCCAAGAGGAAGAGAAAGAATCTATACAGGGGTATCCGCCAGAG
GCCTTGGGGCAAGTGGGCTGCTGAGATCAGAGATCCTCAGAAGGGTGTCC
GTGTTTGGCTTGGTACTTTCAATAGTCCTGAGGAAGCTGCAAGAGCTTATG
ATGCTGAAGCGCGCAGGATTCGTGGCAAGAAGGCCAAGGTTAACTTTCCT
GATGCACCAGAAGTTGGTCAGAAGCGCCGTTCTGGCTCAGCTTCCGCTAA
AGCATCCAAGTCAAGTTTTGGACAGAAGCCTATTGTCAAAGTAACTATGAA
CAGCCTTGCCAACACAAATGCATCCTTCTTCCAATCTGTTAGCTACCCCTCC
AATTCATTTGTTCAGCATGGCAATATACCATTTGTTCCAGCAATGAACTCTAC
TGGTTCTGTTGAGGATCCTATCATGAATCTGCACTCTGACCAGGGAAGTAA
CTCCTTTGGCTGCTCAGACTTGGGCTGGGAGAATGATACCAAGACACCAGA
CATCACTTCCATTGCTCCCATTTCCACTATCGCGGAAGGAGACGAGTCTGC
ATTTGTTGACAGCAATTCAAACAACTCATTTGTGCTTCCTGCCCTGGAGAA
CAGTGCTGTTGATCTCACTGATGGGCTGACAGATTTAGAATCCTATATGAGG
TTTGTTCTGGATGGTGGTCCAAGTGATTCAGTTGATAGCCTTCTGAACCTTG
ATGGATCGCAGGATGTTGGTAGCAACATGGACCTCTGGAGCTTCGACGACA
TGCCCATCGCCGGTGATTTCTTTTGA

ZmEREB139

ATGGCGGCGCCGAGGCTGGAGCGCGGCGGGTTCCAGCTCCCGAACACCGA
GCAGGAGAACTCCCTCTTCCTCCGCGCGCTCATCTCCGTCGTGTCCGGGGA
CACCGCCGCGTTGCTCCCGGAAGCGGCGGCCGTAGAGGCTCCCGCGGCCC
CCGTGGCGTGCGCCAGGTGCGGCGCGGACGGGTGCGCGGCGGCCGGCTG
CTGCGAGCTCGTCGCCGGGTCCAGCAGCGACAGCGACGACGCAGGGTGCT
CCGGGACCCGCGCCAACGGCGGCGGCCTAGGCCTAGGCCTAGGCGCGAGC
AAGCGGAGAGGGAGGGGGAGGAAGGTGATCCAGTACAGGGGCGTGCGGC
GGCGGCCGTGGGGCAAGTGGGCGGCGGAGATCCGCGACCCGCGCCGCGC
CGCGCGCAAGTGGCTCGGCACGTTCGACACCGCCGAGGACGCCGCGCGCG
CCTACGACGTCGCCGCGGTCGAGCTCCGGGGCCAGCGAGCCAAGCTCAAC
TTCCCGGCGCGCGCTGCCGCCGCGGCGCCGGCGGCAGTGCAGCCTCCGCA

CCACCGTCCATTGCCCGATCGGAGCCTCCGCGAGAACTGCGGATCCAACGC
CGCGTCGCCGGTGCACGTGGCGCTGGCGCCGACCACGCAACAGAATATCT
GGGAGGGCTTGAACGAGATCATGATGATGGAGGAGGAACGCAGCTTCTGG
TCTATGCGATGA

ZmEREB69

ATGTGCGGCGGAGCCATCATCGCCGACTTCGTCCCCGCCGGCGCCCGGCGC
CCGGCAACAGACGACACCATGTCCGCCTCCATCCTCTCCGGCGAGGACCAT
CCGGAGCTGCCGCTGCCGCTGCCGGCGCCGGCGCCGGGGCGCAAGACGG
CGTACCGCGGGATCCGGCGCCGCCCGTGGGGCCGGTGGGCGGCGGAGATC
CGCGACCCGCGGAAGGGCGCCCGCGTGTGGCTCGGCACCTACGCCACCCC
GGAGGACGCGGCGCGCGCCTACGACGTGGCGGCGCGCGAGATCCGCGGG
CCCAAGGCCAAGCTCAACTTCCCGCCCGCCGTCGGCGGCGGCGCGCGCGC
GCCGGCGCCGGCGGCGGCCAAGAGGCGCCGCAAGTCCGCGGCGACCGAG
GAGAGCTCGGCCTCGTCTCCTCCTCCTCCGGCGGCGGCGGCGGCCGTCGT
CGTGGCGGGCGGCGGGGAGGAGGCGCTGCGCGACTGCATGTCTGGGCTCG
AGGCGTTCCTGGGGCTGCAAAACGCCGACGAGGGTGGCGGCGTCGAGGC
GTGGGACGCCGTGGACCTCATGTTGGCGTAG

ZmEREB200

ATGTGCGGCGGCGCGATCATCTCCGAGTTCATCCCGCATCGCGGCGCCAAG
CGGGGTCTCTGCGCCGAGGACATCTGGCCGCACGCGGCCGCCGACTTCGA
CGACCTCCTCCACGCGCACGCGCACGCGCACGGCCACGACGACGACGACT
TCGCCGCCGCCGCCTCCTTCCATCCCGACCAAGAGCCGCCGGCCCGCAAG
CGGGAGCGCAAGACCATGTACCGCGGCATCCGGCGCCGCCCCTGGGGCAA
GTGGGCGGCGGAGATCCGGGACCCGGCCAAGGGCGCGCGCGTCTGGCTCG
GCACCTTCGCCACCGCCGAGGCCGCCGCGCGCGCCTACGACCGCGCCGCG
CGCCACATCCGCGGGGCCAAGGCCAAGGTCAACTTCCCCAACGAGGACCC
GCCGCCCGAGTACGACGACGACGACGACGGCCACGCCCACGCCCACGCCG
CCGCGGCGCAGGGGATGCTCGCCATGTCCGCCGGCGGAGGCAGGGACGAC
CACCTAGTCGACTACGACGTCGTCGACGTCATGGGCATGGGCGGCTTCTTC
CAGCACCACGCGTACGTGCCCGACGCCGTGGCGCAGCAGGAGCAGGTGCC
CACGGTCGCGTACGTACACCACCAGCCGCCGCCGCAGCAACCGCAGGGCG
CTGCCGGGATGGACATGTGGACGTTCGACGCCATCAACACGCCCGTGCCC
ATGTGA

ZmEREB116

ATGCATGGGCAGAGAGGCGTCGTCCCTCCTCGGCGCGGAGAGGGGCCAAA
GGTGGAAGCAGCAGCAGGGAACGGCTTTCTCGGCCACAGTTACTCCGCGG
CCCGCGCCGATTACGACGTCGCGGTCATGGCCGCGGCGCTCACGCACGTCG
TCTGCGCCACTGAGCCACCACCGCCGCGCGGGGGCGAGGCGGCGGCGCTT
CCGCCGGGGCCGCGGCAAGGAGGAGGGACGACGGAGCTGCAGGCGGCGG
CGCGGGCACATCAGTACAGGGGCGTGAGGCGGCGGCCGTGGGGCAGGTG
GGCGGCGGAGATACGGGACCCGGAGAAGGCGGCGCGCGTGTGGCTCGGC
ACGTTCGCCACGCCCGAGGAGGCAGCCCGCGCCTACGACGACGCCGCTCG
CAGGTTCAAGGGCGCCAAGGCCAAGCTCAACTTTCCGACGACTACTGCTA
CTGCTACGACGACGACGACATCGCTGTCGTCGCCCCACCAGCTGCTGCGGT

CAGCCGCGGAGTCGGAGACGGCGGGGGAGGAGTTCCCCGACCTCGGGCA
GTACATGCACATACTCCAGAGCAGCAGCGACGCCGACGTTCGGGCCGTCG
CGGCCGGGTTGCCGCTGATGAACCGCCTGCCGCCAGTGGACGGTCGCCAA
GACCATGGCAGCAGCAGCGCGAGCAGCGGGCGCTAA

ZmEREB102

ATGTGCGGCGGCGCCATCCTCTCGGGTTTCATCCCGCCGTCCGGGGTGGCG
GCGGCGGCGGCGGCGGCCAAGAAGCAGCAGCAGCAGCAGCAGCGGTGCC
GTGTGACTGCGGACCTGCTATGGCCGGGGCCCGGCAGTAAGGGAGCTCCC
CAGGACAAGGAGGAGGACTTCGAGGCCGACTTCCGCGAGTTCGAGCGCG
GCCTCGGCGAGGATGACGTGGACAGCGCCGGCGAAGGCGGCGACCCCGA
GGTCCAGGAGCTTCCTCCGCCGGAGCCGACGAGGTTTGCCTTCGCCACCG
CGGCCAAGGCGGCAGTTGATGGCGTGATGACTCCTCCTCCAAAGGATGTCC
AAGGAGATAGAGCAGTAAAAAAGCGCGGCCGGAAGAACCAGTACAGGGG
AATCAGGCAGCGGCCTTGGGGCAAATGGGCAGCTGAGATCAGGGACCCTA
ACAAGGGCGTCCGCGTCTGGCTCGGAACCTACAACACCGCCGAGGAGGCA
GCTAGGGCATACGACGCCGAAGCTCGCAAGATCCGCGGCAAGAAAGCGAA
GGTGAATTTTCCCGATGACGCGACAGGCACTCGTCACAGGCCAACGACCG
ATGAGATCTTTAACAACCTGAAGAACGACGACAACAACAACAACGATGAT
CTGTTTGCGATGTTTGCGTTCGGTGACAATAAGAAGAAGGTTCCTGCAGCG
AAGCCAGCCGCCGCCGAGGGTGGCAGTGGCAGTGGCAGCTTCCTCGTCCC
TGCGCCTGCGGTGGCGGTGGTGCCCGGTAATAAGAGGAGGTCGTCCGCGA
CCAACACTATGCTCTCCGTCTCCGACGACCAGCGCAGCAACTCCTACGGCT
CCGGCTCCTCCGACTTGGTGGGGTCGTGGTCGTGGGACGACGACGCGGCG
GCCGCCGCCATGACCTCGGACTACACCTCCTCGGTCTTCGCTCCCGATAAC
GCCGTGCTGCCGGCGGCGTCTTACACGCAAGGCGGAGCGCCGAAGAGAAT
GAGGAGCAGCTACGGCGGCGCGCCGCCCAGCCTCGCACATGACGCGGCCA
TGCCTGGCTTTGGCCTTGATAAAGTGAGCTACCACCACTACCAGGCGTTGC
CTCCTTATTACGTCGGGAGCAGCAACGCGTCGGTGGGCAACCTCGGTCTTC
TGCAGCAGGCTGATGATGCTCCAGCTCCACAGGATGGGGCGAGCGCCGGG
GATATCTGGAGCCTCGACGAGCTGCTCATGCTGGCAGCAGCTGGTGCTTAT
TGA

ZmEREB160

ATGTGCGGCGGCGCCATCCTGTCGGACATCATCCCGCCGCCGCCACCGCGG
CGGGTCACGGCTGGCCACCTCTGGCCCGAGAGCAAGAAGCCGAGGAGGG
CTGCATCCGGCAGGAGGGGAGCCCCCGTGGAGCAGCATGAGCAGGAGGA
GGATTTCGAGGCCGACTTCGAGGAGTTCGAGGTGGAGTCCGGCGAGTCGG
AGCTCGAGTCCGAGGACGAGCCCAAGCCCTTCGCCGCCCCCAGGAGCGCG
CTCGCCAGAGGTGGACTAAACACTGGTGCAGCTGGTGTCGATGGCCCTGC
TGCAAATTCAGTTAAAAGGAAGAGGAAGAACCAGTTCAGGGGTATCCGCC
GGCGCCCGTGGGGCAAATGGGCTGCTGAGATCAGAGATCCTCGCAAGGGC
GTGCGCGTCTGGCTCGGTACTTTCAACTCCCCCGAAGAAGCTGCCAGAGCT
TACGACGCCGAGGCACGCAGGATCCGCGGCAAGAAGGCTAAAGTCAACTT
CCCGGATGAGGTTCCTACGGCGGTTTCTCAGAAGCGCCGTGCTGCTGGGCC
TGCCTCTCTGAAAGCGCCTAAGATGGACGTTGAGGAGGAGAAGCCGATCA

TCAAGCTCGCAGTGAACAATATGACCAACTCAAACGCATATCACTACCCTG
CCGTCGTCGGCCACAACATCATACCCGAGCCATTCATGCAGACTCAGAACA
TGCCATTCGCTCCTCTGGTGAATTATGCTGCCCTAGTGAACCTGTCTTCAGA
CCAAGGCAGCAACTCGTTCGGTTGCTCGGACTTCAGCCTCGAGAACGACT
CCAGGACCCCTGACATAACTTCGGTGCCTGCGCCCGTTGCCACCTTGGCCG
CCGTTGGCGAGTCTGTGTTCGTCCAGAACACCGCCGGCCATGCTGTGGCGT
CTCCTGCGACGGGGAACACTGGTGTTGATCTCGCCGAGTTGGAGCCGTATA
TGAATTTCCTGATGGACGGTGGTTCAGACGACTCGATCAGCACTCTCTTGA
GCTGTGATGGATCCCAGGACGTGGTCAGCAACATGGACCTTTGGAGCTTCG
AGGACATGCCCATGTCTGCTGGTTTCTACTGA

TaERFVII.5-1A

ATGTGCGGCGGAGCTGTTATCGCCGACTTCGTCCCGGCCGGGGCCCGCCGC
CCGGATGGCTCCTCCGCCGACGTCCCCGGCTCCATCCTCGCCGGTGGGGAG
GTGAAGGAGAAACCGCTGGCGCCGGGGCGGAAGACGGCGTACCGTGGGA
TCAGGCGCCGGCCATGGGGCCGCTGGGCTGCGGAGATCCGGGACCCCACG
AAGGGCGCGCGCGTCTGGCTGGGCACCTACGCCACCGCGGAGGAGGCCGC
GCGCGCCTACGACGTCGCGGCGCGCGATATCCGCGGGCCGAAGGCCAAGC
TCAACTTCCCGCCCGCGGTGGGCGCGCCGCAGGCGGCCGCGGCCGTGGAG
GGGCCGGGGGCGCCCAAGAAGCGTCGGTGGGTCGCTGCCGAGGAGAGTT
CGGCATCTTCGTCTCCCCTTCCGGTCACAGCTGCCGGCGGCACGGAGAGCC
TGCGGGAGCGCATGTCCGGGCTGGAGGCGTTCCTGGGGCTGGAGGACGAC
GACGTGGAGGCCTGGGGGGCCGTCGATCTCATCTTGGATTAG

TaERFVII.5-1B

ATGTGCGGCGGAGCTGTTACAGCCGACTTTGTCCCGGCCGGAGCCCGCCG
CCCGGATGGCTCCTCCGCCGACGTCCCCGGCTCCAGCCTCACCGTCACCGG
TGAGGAGGTGACGGAGAAATCGCCGGCGCCGGGGCGGAAGACGGCGTAC
CGTGGGATCAGGCGCCGGCCATGGGGCCGCTGGGCTGCGGAGATCCGGGA
CCCCAGGAAGGGCGCGCGCGTCTGGCTGGGCACCTACGCCAGCGCGGAGG
AGGCCGCGCGCGCCTACGACGTCGCGGCGCGCGATATCCGCGGGCCGAAG
GCCAAGCTCAACTTCCCACCCGCGGTGGGCGCGCCGCAGGAGGCCGCGGC
CGTTGCAGGGGCGGGGGCGCCCAAGAAGCGTCGCATCGTCGCGGCAGAG
GAGAGCTCCGCGTCTTGGTCTCCACTTCCGGCCCCGGCTAGCGGCGGCGG
CGGCGGCACAGACAGCCTGCGGGAGCGCATGTCCGGCCTGGAGGCGTTCC
TGGGGCTGGAGGACGGCAACGTGGAGCCCTGGGAGGCCGTCAATCTCATC
ATGGAGTAG

TaERFVII.5-1D

ATGTGCGGCGGGGCTGTTATCGCCGACTTCGTCCCGGCCGGGGCCCGCCGC
CCGGATGGCTCCTCCACCGACGTCCCCGGCTCCAGCCTCACCGTCACCGGT
GAGGAGGTGACGGAGAAACCGCCGGCGCCGGGGCGGAAGACGGCGTACC
GTGGGATCAGGCGCCGGCCATGGGGCCGCTGGGCTGCGGAGATCCGGGAC
CCCAGGAAGGGCGCGCGCGTCTGGCTGGGCACCTACGCTACCGCGGAGGA
GGCCGCCCGCGCCTACGACGTCGCGGCGCGCGATATCCGCGGGCCGAAGG
CCAAGCTCAACTTCCCGCCCGCCGTGGGCGCGCCGCAGGCGGCCGCAGCC
GTGGAGGGGGCGGGGGCGCACAAGAAGCGTCGGATGGTCGCGGCAGAGG

AGAGCTCCGCGTCTTGGTCTCCACTTCCGGCCACGGCCACCGGAGGCGGC
GGCACAGAGAGCCTGCGGGAGCGCATGTCCGGGCTGGAGGCGTTCCTGGG
GCTGGAGGACGGCGACGTGGAGCCCTGGGAGGCCGTCGATCTCATCTTGG
AGTAG

TaERFVII.2-2A

ATGTGTGGCGGCGCGATCATCTCCGACTTCATCCCGGAGCGCGACCACCGC
GGCGGGAGCAAGCGGAGCCTCTGCACCGCCGACTTCTGGCCAAATGCGGC
CGGCGCCGGCGCCGCGTTCGACGACCCTACCGGCCACCACGACTTCTACC
CAGCCGACCTGACCGGCGCCGGCGCCTTCACGCCTGAGCACCAAGCGGAG
GAGGAGCCGAGCAGGAAGCGGGAGCGCAAGACGATGTACCGCGGCATCC
GGCGGCGGCCGTGGGGCAAGTGGGCGGCGGAGATCCGCGACCCGGCCAA
GGGGGCGCGCGTCTGGCTCGGCACCTTCGCCACCCCCGAGGGCGCCGCGC
GCGCCTACGACCGCGCCGCCCGCCGCATCCGCGGGACCAAGGCCAAGGTC
AACTTCCCCAACGAGGACCCGCCCCTCGACCTCGACGACTACGACGTCGC
CAACGTGGCCGGCTTCATCCACCAGCCGTCGTACATGGCGCCAGAGGTGG
CGTACGCGCACCAGCTGCCGCAGCAGGACGAGCCCGGGATGGAGCTCTGG
AACTTTGGCAACATCAACGCTCAAGTGCCTATGTGA

TaERFVII.2-2B

ATGTGTGGCGGCGCGATCATCTCCGACTTCACCCCGGAGCGCGACCACCGC
GGCGGCAGCAAGCGGGGCCTCTGCACCGCTGACTTCTGGCCGCATGCCGC
CGCGTTCGACGACCCTACCGACCACCACGACTTCTACCCCGACGAGCTGA
CCGGCGCCCGCTCGTTCCCTCCCCAGCACCGAGCGGCAGAGGAGGAGCCG
AGCAGGAAGCGGGAGCGCAAGACGATGTACCGCGGCATCCGGCGGCGAC
CGTGGGGCAAGTGGGCGGCGGAGATCCGCGACCCGGCCAAGGGCGCGCG
CGTCTGGCTCGGCACCTTCGCCACCCCCGAGGGCGCCGCGCGCGCCTACG
ACCGCGCCGCCCGCCGCATCCGCGGGACCAAGGCCAAGGTCAACTTCCCC
AACGAGGACCCGCCCCTCGACCTCGACGACTACGACGTTGCCAACGGCGG
CTACAACGTCGCCGGCTTCTTCCACCAGCCGTCCTACATGGCCGAAGCCAC
CGCGCCGGTGCCGGAGGCGGCGTACGCTCACCAGCTGCCGCATCATGACG
AGCCCGGGATGGAGCTCTGGAACTTTGACAACATCAACACCCAAGTGCCT
ATGTGA

TaERFVII.2-2D

ATGTGTGGCGGCGCGATCATCTCCGACTTCACCCCGGAGCGCGACCACCGC
GGCGGCAGCAAGCGCAGCCTCTGCACCGCCGACTTCTGGCCGCATGCCGC
CGCCGCGTTCGACGACCCTACCGGCCACCACGACTTCTACCCAGCCGACCT
GACCGGCGCCTGCTCGTTCCCTCCCCAGCACCAAGCTGCGGCAGGGGAGG
AGCCGAGCAGGAAGCGGGAGCGCAAGACGATGTACCGCGGCATCCGGCG
GCGGCCCTGGGGCAAGTGGGCGGCGGAGATCCGCGACCCGGCCAAGGGC
GCGCGCGTCTGGCTCGGCACCTTCGCCACCGCCGAGGGCGCCGCCCGCGC
CTACGACCGCGCCGCCCGCCGCATCCGCGGGACCAAGGCCAAGGTCAACT
TCCCCAACGAGGACCCGCCGCTCGACCTGGACGACTACGACGTCGCCAAC
GTCGCCGGCTTCTTCCACCAGCCGTCGTACATGGCCGATGCCGCCGCGCCG
GCGACGGAGGTGGCGTACGCGCACCAGCTGCCCCAGCAGGACGAGCCCG
GGATGGAGCTCTGGAACTTTGACAACATCAACACGCAAGTGATATAG

TaERFVII.4-4A
ATGTGCGGCGGCGCCATCCTAGCGGAGCTGATCCCGCCGTCGGCGGGCCGT
GCCTCGAAGCAGGTGGCCGCGGGCCGGGCCTCGCCAAAGAAGGCCGGCA
AGAGCAAGGGGCATAGGTACGGCAGCGTCGCCGATGTCGACGACTTCGAG
GCCGCCTTCGAGAACTTCGACGACGACCTCGACCTGCAGGCGGAGGAGGA
CGGCGACGAACATGTCGTTTTTGCATCCAAGCCTGCGTTCTCTCCGGGCCT
GGCCTACGATGATGGCCGCGCGGCGCAGGCGGCGAGCAAGAAGAAGAGC
GTCCGCCCCCTCCACGGCATCCGGCAGCGGCCGTGGGGTAAGTGGGCGGC
GGAGATCCGCGACCCGCACAAGGGCACCCGCGTCTGGCTCGGCACCTTCG
ACACGGCCGACGATGCCGCCCGGGCCTACGACGTCGCCGCCCGACGCCTC
CGCGGCAGCAAGGCCAAGGTCAACTTCCCCGACGCGGCCAGGGCCGGGG
CGCGCCCGCGCCGCGCCAGCCGCAGAACTGCGCAGAAACCGCAGCGCCC
GCCTGCGCGGACGACGGCGTACTCTGCCACCGCAGCACCACGCGCACGGC
CGGAGCAGGACGCTATGATGGTCAAGCCCGAGCGGATGGAGTTTTCCGAC
GTGGACGCGTTCGTCGACCTCACCGCCGCCGTCGCCGTGCTACCGCCTGTC
ACGGCGAGCTCCTTCGCCGACAAGATGCCGAGGGTCGACGAGGACTCGTC
GGAGGGGAGCGGCGGCGGCTCCATGTTGGGGTTCGCCGACGACCTTGGGT
TCGATCCCTTCATGATGCTCCAGTACGAATCCATGGACAGCCTCTTCGCCGG
AGACGCCGTCATCCAGGATGCCCGCGGTGTGGACGGCGGCATGGACGGCG
TCAGCCTCTGGAGCTTCGAGGAGTTCCCCATGGACAGCGCCATTTTCTGA
TaERFVII.4-4B
ATGTGCGGCGGCGCCATCCTAGCGGAGCTGATCCCGCCGTCGGCGGCGGG
CCGTGCGCCGAAGCAGGTGGCCGCGGGCCAGGCCTCGTCCAAGAAAGGC
GGCATGAGCAAGAGGCACCACAGCAGCATCCCCGACGTCGACGACTTCGA
GGCCGCCTTCGAGGACTTCGATGACGACTTCGACCTGCAGGCGGAGGAGG
ACGGCGGCGACCATGTCGTTTTCGCATCCAAGCCTGCGTTCTATCCGGCATA
CGGTGGTGGCCGCCGCGCGGTGCAGGCGGCAAGCATGAAGAAGCGCGTC
CTCCACGGCATCCGGCAGCGGCCGTGGGGCAAGTGGGCGGCGGAGATCCG
CGACCCGCACAAGGGCACCCGCGTCTGGCTCGGCACGTTCGACACGGCCG
ATGACGCCGCCCGGGCCTACGACGTCGCCGCCCGACGCCTCCGCGGCAGC
AAGGCCAAGGTCAACTTCCCCGACGCGGCCAGGGCCGGGGCGCGCCCGC
GCCGCGCCAGCCGTAGAACCGCGCAGAAACCGCCATGCCCCCCTGCGGGG
ACGACGGCGTACTCTGCCACCGCAGCATCACGCGCACAGCCGGAGCAGGA
CACTATGATGGCCAAGCCCGAGCGGATGGAGTTTTCGGACGTGGACACGTT
CGTTGACCTGACCGCCGCCGTCGCCGCGCTACCGCCTGTCACGGCGAGCT
CCTTCGCCGACAAGATGCCGAGGGTCGACGAGGACTCGTCGGAGGGGAGC
GGCGGCGGCGCCATGCTGGGGTTCGCCGACGAGCTTGGGTTCGAACCGTT
CATGATGTTCCAGTACGAATCCATGGACAGCCTCTTCGCCGGACACGCCGT
CATCCAGGATGCCCGCGGTGTGGACGGCGGCATGGACGGCGTTAGCCTCTG
GAGCTTCGACGAGTTCCCCATGGACAGCGCCATTTTCTGA
TaERFVII.4-4D
ATGTGCGGCGGCGCCATCCTAGCGGAGCTGATCCCGCCGTCGGCGGGCCGT
GCCTCGAAGCAGGTGGCCGCGGGCCGGGCCTCGCCAAAGAAGGCCGGCA
AGAGCAAGGGGCAGAAGTACGGCAGCGTCGCCGATGTCGACGACTTCGAG

GCCGCCTTCGAGAACTTCGATGACGACCTAGACCTGCAGGCGGAGGAGGA
CGGCGACGACCATGTCGTTTTCGCATCCAGGCCTGCGTTCTCCCCGGCCTA
CGATGGTGGCCGCCGCGCGGTGCCGGCGGCGAGCAAGAAGAAGAGCGTC
CGCCCCCTCCACGGCATCCGGCAGCGGCCGTGGGGCAAGTGGGCGGCGGA
GATCCGCGACCCGCACAAGGGCACCCGCGCCTGGCTCGGCACCTTCGACA
CGGCCGATGATGCCGCCCGGGCCTACGACGTCGCCGCCCGTCGCCTCCGTG
GCAGCAAGGCCAAGGTCAACTTCCCCGACGCGGCCAGGGCCGGGGCGCG
CCCGCGCCGCGCCAGCCGTAGAACCGCGCAGAAACCGCCATGCCCCCCTG
CGAGGATGACGGCGTACTCTGCCACCGCAGCAGCACGCGCACAGCCGGAG
CAGGACTCTATGATGGTCAAGCCCGAGCTGATGGAGTCTTTAGACATGGAC
GCCCTTGTCGACCTGACCACTGCTGTCACCGCACTACCGACTGTCATGGCA
AGCTCCTTCGCCGACAAGATGCCGAGGGTCGACGAGGACTCGTCGGAGGG
GAGCGGTGGCGGCGCCATGCTGGGGTTCGCCGACGAGCTTGGGTTCGATC
CGTTCATGATGTTCCCGTACGAATCCATGGACAGCCTCTTCGCCGGAGACG
CTGTCATCCAGGATGCCCGCGGTGTGGACGGCGGCATGGACGGCGTTAGCC
TCTGGAGCTTCGACGAGTTCCCCATGGACAGCGCCATTTTCTGA
TaERFVII.3-4A
ATGTGCGGCGGCGCCATCCTAGCGCAGCTGATCCCGCCGTCGGCGGGCCGT
GCCCCGAAGCAGGTGGCCGCCGGCGGGGTCGCGCCCAAGAATGGCGGCAT
GAGCAAGAGGCACCACAGCAGCACCCCCGATGTCGACGACTTCGAGGCCG
CCTTCGAGGACTTCGAGGACGACGTCGACCTGCAGGCGGAGGACGACGG
CGACGACCATGCCGTTTTTGCATCCAAGCCCGCCTTCTCCACGGCCTACTAT
GATGGCCGCGCGGCGCAGGCGGCCAGCAGGAAGAAGAGCGTCCGCCGCC
TCCACGGCATCCGGCAGCGGCCGTGGGGCAAGTGGGCGGCGGAGATCCG
CGACCCGCACAAGGGCACCCGCGTCTGGCTCGGAACGTTCGACACGGCCG
ATGACGCCGCCCGGGCCTACGACGTCGCCGCCCGCCGCCTCCGTGGCATCA
AGGCCAAGGTCAACTTCCCCGACGCGGCGAGGGCCGGGGCGCGCTCGCG
CCGCGCCAGCCGGAGAACTGCGCAGAAACCGCAGTGCCCGCCTGTGCGG
ACGACGGCGTACTCTGCCACCGCAGCTGCACACGCACACGTAGTACAGGC
GGAGCAGGACGCTATGATGATCAAACCTGAGCTGATGGAGTTTTTGATGC
GGACGCGTTCGTCGACCTGACCGCCGCCGTCGCCGCGCTGCCGCCTGTCA
CTGGCGCGAAGAAGCCGATGGTCGATGAGGATTCGTCGGATCGGAGCGGC
GGCTGCGCCATGCTGGTGTTCGCCGACGAGCTTGGGTTCGATCCGTTCACG
CTGTTCCAGCTCCCCTGCTCGGACACCTACGAATCCATCGACAGCCTCTTC
GCCGGGGACGCCGTCATCCAGGATGCCCTCGGCGTGGACACTGGCATGGA
GGGCGTCAGCCTCTGGAGCTTCGAGGAGTTCCCCATGGACAGCGCCATTTT
TTGA
TaERFVII.3-4B
ATGTGCGGCGGCGCCATCCTAGCGAAGCTGATCCCGCCGACGCCGCCGTCG
GCGGGCCGTGCCCCGAAGCAGGTGGCCGCGGGCGGGGTCTCGCCCAAGA
AGGGCGGCATGAACAAGACGCACCACAGCAGCACCCCCGATGTCGACGAC
TTCGAGGCCGCCTTCGAGGACTTCGATGACGACTTCCACCTGCAGGCGGA
GGAGGACGGCGACGACCATGTCGTTTTTGCATCCAAACCTGCCTTCTCCCC
GGCCTACGATGATGGCCGCGCGGCGCAGGCGGCGAGCAGGAAGAAGAGC

GTCCGCCGCCTCCACGGCATCCGTCAGCGGCCGTGGGGCAAGTGGGCGGC
GGAGATCCGCGACCCGCACAAGGGCACCCGCGTCTGGCTCGGCACGTTCG
ACACGGCCGATGATGCCGCCCGGGCCTACGACGTCGCCGCCCACCGCCTCC
GTGGCAGCAAGGCCAAAGTCAACTTCCCCAACGGGACCAGGGCTGGGGC
GCGCCTGCAACGTGCCAGCCGGAGAACCGCTTCGAAACGGCAGTGCCCCC
CTGCGCGGACGACGGCGTACTCTGCTGCACACGCACAGAAGGAGCGGGAC
GCTATGGTGGCCAAGCCTGAGCTGATGGAGTCTTTCGACATGGACGCCTTC
GTCGACCTGACCACTGCTTTCACCACGCTACCGCCTGTCATGGCAAGCT
CCTTCGCCGACACTGGCGCGAAGAAGCCGATGGTCGATGAGGATTCGTCG
GATGGGAGCGGCGGCGATGCCATGCTGGGGTTCGATCCGTTCATGCTGTTC
CAGCTCCCCTGCTCGGACACGTACGAATCCATCGACAGCCTCTTCGCCGGC
GACGCCGTCATCCAGGATGCCCTCGGCGTGGACAGTGGCATGGAGGGCGT
CAGCCTCTGGAGCTTCGAGGAGTTCCCCATGGACAGCGCCATTTTTTGA

TaERFVII.6-4A

ATGTGTGGCGGAGCGATCCTCGCCGAGCTCATCCCGGGGGGCGCGCCGGC
GCGGCGCGCCGCGTCCGGCCACGTCTGGCCGGGCAAGGCCGCCGACGACT
TCGAGGCCGCGTTCCGGGACTTCAACGAGGAGGAGGACGTGGTGGTGGTG
GTGGAGGAGGAGGTGGCCGAGAGCAAGCCCTTCGTGTTCGCCGCCTCGCC
CAAGAAGCCGCAGCAGCAGCGGGAGGAGGAGGAGCAGGCGGCGCCCGCC
CGCCGGAGGAAGCCGGCGCAGTACCGCGGCGTGCGGCGCCGGCCGTGGG
GCAAGTGGGCCGCCGAGATCCGCGACCCCGTCAAGGGCGTCCGCGTCTGG
CTCGGCACCTTCCCCTCCGCCGAGGCCGCCGCGCTCGCCTACGACGGCGCC
GCGCGCGCCATCCGCGGGCCCAGGGCCAAGCTCAACTTCCCTCCCTCCTCT
GCCGTCGCCGCCGCGGCCCCGGGATCGCGCAAGCGCGTCCGCGCAGAAGC
CCTCGCGGCCCCCGCCGCCAAGCCGGCGCCGGTCGTCACCCTCGTCGACG
ACGAGGAGGAGCACGCGTCGTCCTTGTTCGTCAAGCACGAGGCCGAGCCG
AGCGAGGGCTCCGAGTCCAGCGGCGCCCTCCCCGACTTCTCGTGGCAGGG
CATGTCGGCGCTCGACGAGGCCCCGGCGTACCCCGCCCCGGAGCCCGAGA
CCGAGCAGCTGACAAAGCGGGCGAGGACGACGGAGGCGGAGGACATCGA
CGAGGGCATGTCGGCCCACCCGGCGTCCGACTCCGACTCCGACGCGCTCT
TCGACGCCCTCCTCTTCGCCGACCAGTTCGCCTACTTCAACGGCGGCGCCT
ACGAGTCCCTGGACAGCCTCTTCAGCGCCGACGCCGTGCAGAGCGGCGCC
ACCGCCACCGCCGCCGCCGCGGACGAGGCGGGCCTGGGGCTCTGGAGCTT
CGACGACGACTGCCTCGTCGACGAGTGCAGCCTGTCCTTCTAG

TaERFVII.6-4B

ATGTGTGGCGGAGCGATCCTCGCCGAGCTCATCCCGGGCGCGCCGGCGCG
GCGCGCCACGTCCGGCCATGGCCACGTCTGGCCGGGCAAGGGCGCCAAGC
AGACCAAGGCCGCCGCGGCCGACGACTTCGAGGCCGCGTTCCGGGAGTTC
AACGAGGACTCTGATGTGGAGGACGACGTCGTGATGGTGATGGAGCGGCG
GGAGGAGGTGGCCGAGAGCAAGCCCTTCGTGTTCGCCGCTTCGCCCAAGA
AGCAGCAGCAGGAGGAGGAGGAGGCGGCGCCCGTCCGCCGCAGGAAGCC
GGCGCAGTACCGGGGCGTGCGGCGCCGGCCGTGGGGCAAGTGGGCCGCC
GAGATCCGCGACCCCGTCAAGGGCGTCCGCGTCTGGCTCGGCACCTTCCC
CTCCGCCGAGGCCGCCGCGCTCGCCTACGACGAAGCCGCGCGCGCCATCC

GCGGGCCCAGGGCCAAGCTCAACTTCTCCTCCTCCTCTGCCGTCGCCGCCG
CGGCCCCGGGAGCGCGCAAGCGCGGCCGCGCCGCGCCCCCCGCTGCCAA
GCCAGCGCCGGTCATCACCCTCGTCGACGATGAGGAGGAGCACGCGTCGT
CCTTCGTCAAGCACGAGGCCGAGGCGAGCGAGGGCTCCGAGTCCAGCGG
CGCCCTCCCCGACTTCTCGTGGCAGGGCATATCGGCGTTCGACGAGGCCCC
GGCGTACCCCGCCCCGGAGCCGGAGACCGAGCAGCTGACAAAGCGGGCG
AGGACGACGGAGGCGGAAGACACCGACGAGGGCATGTCGGCCCACCCGG
CATCAGACTCCGACTCCGACGCGCTCTTCGACGCCCTCCTCTTCGCCGACC
AGTTCGCCTTCTTCAACGGCGGCGCCTACGAGTCCCTCGACAGCCTCTTCA
GCGCCGACGCCGTGCAGAGCAGCGCCACCGCCACCGCCGTGAACGAGGC
GGCCCTGGGGCTCTGGACCTTCGACGACGACTGCCTCGTCGACGAGTGCA
GCCTGTCGTTCTAG

TaERFVII.1-5A

ATGTGCGGCGGCGCCATCATCTACGACTACATCCCGGCGCACCGCCGCCGG
GTGTCCACCGCCGACTTCTGGCCCGACGCCGACCATTCCGACGCCCACAG
CGCCGCCCCCGACAAAGCGCCGCGCGCGAAGCGGGGGCGGACGAACCAG
TACCGCGGCATCCGGCAGCGGCCGTGGGGCAAGTGGGCGGCGGAGATCCG
CGACCCCGTGAAGGGCGTCCGCGTCTGGCTCGGCACCTACCCCACCGCCG
AGGCCGCCGCGCGCGCCTACGACCGCGCCGCGCGCCGCATCAGGGGCGCC
AAGGCCAAGGTCAACTTCCCCAACGAGATCCTGGTCGGCGCGCCCGCGCA
CGAGGCCCCGTGCACGATGGCGGCCGTGCTCCCTTCCCCCAAGAAAGAGG
AGGAGCCCGCGGCGTGCTCCTGCGAGGAGGTGAAGGCGCTCTCCGAGGA
GCTGATGGCGTACGAGAGCTACATGAGCTTCCTCGGGGTCCCCTACATGGA
GGGCGGGTCCGCGGCCGCGACCGCCCCTGCCGCCGTCGGTGTCGCCGCCG
AGGATGCACCGGCCGAGCTATGGAGCTTCGAGGACAGCTACTACCCGGGG
CCTCTGGGGCTCTGA

TaERFVII.1-5B

ATGTGCGGCGGCGCCATCATCTACGACTACATCCCGGCGCACCGCCGCCGG
GTGTCCACCGCCGACTTCTGGCCCGACGCCGACCATTCCGACGCCTACAGC
GCCGCCCCCGACAAAGCGCCGCGCGCGAAGCGAGGGCGGACGAACCAGT
ACCGCGGCATCCGGCAGCGGCCGTGGGGGAAGTGGGCGGCGGAGATCCG
CGACCCCGTCAAGGGCGTCCGCGTCTGGCTCGGCACCTACCCCACCGCCG
AGGCCGCCGCGCGCGCCTACGACCGCGCCGCGCGCCGCATCAGGGGCGCC
AAGGCCAAGGTCAACTTCCCCAACGAAATCCTCGTCAGCGCGCCCGCGCA
CGAGGCCTCGTGCACGATGGCGGCTGCTCCGCCGGCGGCCGTGCTCCCATC
CCCCAAGAAGGAGGAGGAGGGGGAGGGGTTGGAGCCCGCGGCGTGCTCC
TGCGAGGAGGTGAAGGCGCTCTCCGAGGAGCTGATGGCGTACGAGAACTA
CATGAGCTTCCTCGGGGTCCCCTACATGGAGGGCGGGGCCGCGGCCGCCC
CTGCCGCCGAGGAGGCGCCGGCCGAGCTATGGAGCTTCGAGGACAGCTAC
TACCCGGGGCCTCTTGGGCTCTGA

TaERFVII.1-5D

ATGTGCGGCGGCGCCATCATCTACGACTACATCCCGGCGCACCGCCGCCGG
GTGTCCACCGCCGACTTCTGGCCCGACGCCAACGACCACTCCGACGCCCA
CAGCACCGCCCCCGACAAAGCGCCGCGCGCGAAGCGGGGGCGGACGAAC

CAGTACCGCGGCATCCGGCAGCGGCCGTGGGGAAGTGGGCGGCGGAGAT
CCGCGACCCCGTCAAGGGCGTCCGCGTCTGGCTCGGCACCTACCCCACCG
CCGAGGCCGCCGCGCGCGCCTACGACCGCGCCGCGCGCCGCATCAGGGGC
GCCAAGGCCAAGGTCAACTTCCCCAACGAGATCCTCGTCGGCGCGCCCGC
GCACGAGGCCCCGTGCACGATGGCGGCCGTGCTCCCTTCCCCCAAGAAAG
AGGAGGAGCCCGCGGCGTGCTCCTGCGAGGAGGTGAAGGCGCTCTCCGA
GGAGCTGATGGCGTACGAGAGCTACATGAGCTTCCTCGGGGTCCCCTACAT
GGAGGGCGGGGCCGCGGCCGCCCCTGCCGCCGAGGAGGCGCCGGCCGAG
CTATGGAGCTTCGAGGACAGCTACTACCCAGGGCCTCTGGGGCTCTGA
TaERFVII.7-5A
ATGTGCGGCGGAGCCATCCTCGCGGGCTTCATCCCGCCGTCGGCGGCCGCC
AAGGCGGCGGCGGCCAAGAAGAAGCAGCAGCAGCGCAGCGTGACGGCCG
ACTCGCTCTGGCCGGGCCTGCAGAAGAAGGCGGCGGACGAGGAGGACTT
CGAGGCCGACTTCCGCGAATTCGAGCGGGACTCCAGCGACGACGACGCCG
CGGTCGAGGAGGTCCCCCCGCCGCCGGCCGCGGCGGGGTTCGCCTTCGCC
GCCGCCGCCGAGGTCGCGCCGCCGGCCCCTGCCCGCCTAGATGCTGTTCAA
CGTGATGGACCTGCTGCCAAACAAGTAAAGCGCGTTCGGAAGAATCAGTA
CAGAGGGATCCGCCAGCGTCCCTGGGGAAAATGGGCAGCTGAAATCCGTG
ACCCTAGCAAGGGTGTCCGGGTTTGGCTCGGGACATACGACACTGCTGAG
GAGGCAGCCAGGGCATATGATGCTGAAGCCCGCAAGATCCGTGGCAAGAA
GGCCAAGGTCAATTTTCCTGAGGATGCTCCAACTGTTCAGAAGTCCACCCT
GAAGCCAACTGCTGCTAAATCAGCAAAGCTGGCTCCACCTCCGAAGGCCT
GTGAGGATGAGCCTTTCAATCATGTGAGCAGAGGAGACAATGATCTGTTTG
CGATGTTCGCCTTCAATGACAAGAAGGTTCCTGCAAAGCCAGCTGAAAGT
GTGGATTCCCTTCTTCCGGTGAAACTGAAACCTCCTACTGAGACATTCGGG
ATGAACATGCTCTCTGATCAGAGCAGCAACTCGTTTGGCTCTACTGACTTT
GGGTGGGACGACGAGGCCATGACCCCAGACTACACATCCGTCTTCGTTCCG
AATGCTGCCATGCCGGCGTATGTCGAGCCCGCTTACCTGCAAGGTGGAGCT
CCAAAGAGACTGAGGAACAACTTTGGCGTGGCCGTGCTGCCTCAGGGAAA
TGGTGCACAAGACATCCCTGCTTTTGACCATGAGATGAAGTACTCGTTGCC
TTATGTCGAGAGCAGCTCGGACGGATCAATGGACAGCCTTCTGCTGAATGG
TGCGATGCAGAACGGGGCAAGCAGCGGGGATCTCTGGAGCCTCGACGAGC
TCTTCATGGCGGCCGGTGGTTACTGA
TaERFVII.7-5B
ATGTGCGGCGGAGCCATCCTCGCGGGCTTCATCCCGCCGTCGGCGGCCGCC
GCGGCGGCCAAGGCGGCGGCAGCCAAGAAGCAGCAGCAGCAGCAGCAGC
AGCAGCAGCGCAGCGTGACGGCCGACTCGCTCTGGCCGGGCCTGCGGAA
GAAGCCGGCCGAAGAGGAGGACTTCGAGGCCGACTTCCGCGACTTCGAG
CGGGACTCCAACGACGACGACGACGCGGTCGAGGAGGTCCCCCCACCGC
CGGCCACGGCGGGCTTCGCCTTCGCCGCCGCGGCCGAGGTCGCGCTCCCG
GCTCCGACCCGCCTAGATGCTATTCAACATGATGGACCTGCTGCCAAATCA
GTGAAGCGCGTTCGGAAGAATCAGTACAGAGGGATCCGCCAGCGTCCCTG
GGGGAAATGGGCAGCTGAAATCCGTGACCCTAGCAAGGGTGTCCGGGTTT
GGCTCGGGACATACGACACTGCTGAGGAGGCAGCCAGGGCATATGACGCT

GAAGCCCGCAAGATCCGTGGCAAGAAGGCCAAGGTCAATTTTCCTGAGGA
GGCTCCAACTGTTCAGAAGTCCACCCTGAAGCCAACTGCTGTGAAATCAG
CAAAGCTGGCTCCACCTCCGAAGACCTGCGAGGATGAGCCCTTCAATCAC
CTGAGCAGAGGAGACAATGATTTGTTCGCGATGTTTGCCTTCAATGACAAG
AAGGTTTCTGCAAAGCCAGCTGAAAGTGTGGATTCCCTTCTTCCAGTGAAA
CCTCTTGTGCCCACTGAGACATTCGGGATGAACATGCTCTCTGACCAGAGC
AGCAATTCATTTGGCTCCACTGACTTTGGGTGGGACGACGAGGCCATGACC
CCAGACTACACATCCGTCTTCGTTCCGAATGCTGCTGCCATGCCGGCATACG
GCGAGCCCGCTTACCTGCAAGGTGGAGCTCCAAAGAGAATGAGGAACAAC
TTTGGTGTAGCCGTGCTGCCTCAGGGAAATGGTGCACAAGACATCCCTGCT
TTTGACCATGAGATGAAGTACTCGTTGCCTTATGTCGAGAGCAGCTCGGAC
GGATCGATGGACAGCCTTCTGCTGAATGGTGCGATGCAGGACGGGGCAAG
CAGTGGGGATCTCTGGAGCCTTGATGAGCTCTTCATGGCGGCTGGTGGTTA
TTGA

TaERFVII.7-5D

ATGTGCGGCGGAGCCATCCTCGCGGGCTTCATCCCGCCGTCGGCGGCCGCC
GCGGCGGCCAAGGCGGCGGCAGCCAAGAAGAAGCAGCAGCAGCGCAGCG
TGACGGCCGACTCGCTGTGGCCGGGCCTGCGGAAAAAGGCGGCCGAGGA
GGAGGACTTCGAGGCCGACTTCCGCGACTTCGAGCGGGACTCCAGCGACG
ACGACGCCGTGGTCGAGGAGGTTCCACCGCCGCCGGCCTCGGCGGGTTTC
GCCTTCGCCGCCGCGGCCGAGGTCGCGCCCCCGGCCCCTGCCCGCCTAGAT
GCTGTTCAACATGATGGACCTGCTGCCAAACAAGTAAAGCGCGTTCGGAA
GAATCAGTACAGAGGGATCCGCCAGCGTCCCTGGGGGAAATGGGCAGCTG
AAATCCGTGACCCTAGCAAGGGTGTCCGGGTTTGGCTCGGGACATACGAC
ACTGCTGAGGAGGCAGCAAGGGCATATGATGCTGAAGCCCGCAAGATTCG
TGGCAAGAAGGCCAAGGTCAATTTTCCTGAGGATGCTCCAACTGTTCAGA
AGTCTACTCTGAAGCCAACTGCCGCTAAATCAGCAAAGCTGGCTCCACCTC
CGAAGGCCTGCGAGGATGAGCCTTTCAATCATCTGAGCAGAGGAGACAAT
GATTTGTTCGCGATGTTCGCCTTCAATGACAAGAAAGTTCCTGCGAAGCCA
GCTGAAAGTGTGGATTCCCTTCTTCCGGTGAAACCTCTTGTGCCCACTGAG
ACATTCGGGATGAACATGCTCTCTGACCAGAGTAGCAACTCATTTGGCTCT
ACTGACTTTGGGTGGGACGATGAGGTCATGACCCCGGACTACACGTCCGTC
TTCGTCCCGAATGCTGCTGCCATGCCGGCATACGGCGAGCCCGCTTACCTG
CAAGGTGGAGCTCCAAAGAGAATGAGGAACAACTTTGGCGTGGCCGTGCT
GCCTCAGGGAAATGTTGCACAAGACATCCCTGCTTTTGACCATGAGATGAA
GTACTCGTTGCCTTATGTTGAGAGCAGCTCGGACGGATCAATGGACAGCCT
TCTGCTGAATGGTGCGATGCAGGACGGGGCAAGCAGTGGGGATCTCTGGA
GCCTCGATGAGCTCTTCATGGCGGCTGGTGGTTACTGA

TaERFVII.8-6A

ATGTGCGGCGGCGCGATCCTCAAGGACCTCAAGGTCCCCGCGCCGGCGCG
GAAGGTGACGGCGGCGGTGCTGTGGCCCGAGAAGAACAAGCCCAAGCGG
GCCGACGGCGGGGGCTGGCGCCTCGCGGGGCTCGGCCGGCGCGGGGGGC
GCGGGCTGGACGACGGCGAGGAGGACTTCCAGGCCGACTTCGAGGAGTT
CGAGGCCGACTCCGGGGACTCCGACGTGGAGCTCGGGCGCGCCGGGGTTG

CTGGGAAAGACGGCGACGACGAGGTTGTCGAGATCAAGCCCTTCGCCGCC
GTCAAAAGGTCCCTCTCCCAAGATGACTTAAGCACCATGACTACTGCTGGT
TTTGATGGTCCTGCACAAAGGTCAGCAAAAAGGAAGAGAAAGAACGAGT
TCAGGGGTATCCGCCAGCGCCCCTGGGGTAAGTGGGCTGCTGAAATCAGA
GATCCTAGCAAGGGTGTCCGTGTCTGGCTTGGTACTTTCAACAGTGCTGAA
GAAGCTGCAAGAGCTTATGATGTTGAAGCACGAAGGATCCGTGGCAAGAA
GGCCAAGGTTAACTTTCCAGAGGAACCAACAGTTCCTCAGAAGCGCCGTG
CTTGCCCTGCTGCTCCTAAAGTTCCCAAGTCAAGCGTAGCACAGGAACCTA
CCGTCATACCAGCAGTCAACAACCTTGCCAACCCAAATGCTTTCGTCTACC
CATCTGCTGACTTTGCATCAAAGCAGCCGCTTGTTCAGCCTGACAACATGC
CATTTGTTCCTGCAATTAACTCTGCTGCCTCTGTTGAAGCTCCTGTTATGAA
TATGTACTCTGACCAGGGAAGCAACTCCTTTGGCTGCTCTGACTTGGGCTG
GGAGTATGACACCAAGACTCCAGATATATCATCCATTGCTCCCATTTCCACC
ATTGCTGAAGGAACAGAGTCTGCAGTTCTCCAGAGTAAAACCTACAACCC
AGCGGTGATTGCTGAAGGAGCTGAATCTGCGCCTGTCCAGAGCAACACCT
ACAACTCAGTGGTGCCTCCTGTTATGGAGAACAATGCTGTTGATTTTGAAC
CTTGGATGAGGTTTCTTTTGGATGATGGCGTGGATGAGCCGATTGATAGCCT
TCTGAATTTTGATGTGCCTCAGGATGTCGTTGGCAACATGGACCTTTGGAG
CTTCGATGACATGCCCATCTGTGGCAAAATTTTCTGA

TaERFVII.8-6B

ATGTGCGGCGGCGCGATCCTCAAGGGCCTCAAGGTCCCCGCGCCGGCGCG
GAAGGTGACGGCGGCTGTGCTGTGGCCCGAGAAGAACAAGCCCAAGCGG
GCCGACGGCGGCGCCCGGCACCTCGCGGGGCTCGGCCGGCGTGGGGGGC
TCGGGCTGGACGACGGCGAGGCGGACTTCGAGGCCGACTTCGAGGAGTTC
GAGGCCGACTCCGGGGACTCCGACCAGGAGCTCGGGCGCGGCGGGGTGG
CTGAGAAGGACGGCGACGACGAGGTCGTCGAGACCAAGCCCTTCGCCGC
CGTCAAGAGGTCCCTCTCCCAAGATGACTTAAGCACCATGACCACTGCTGG
TTTTGATGGTCCTGCACAAAGGTCAGCAAAAAGGAAGAGAAAGAACGAAT
TCAGGGGTATCCGCCAGCGCCCCTGGGGTAAGTGGGCTGCTGAAATCAGA
GATCCTAGCAAGGGTGTCCGTGTTTGGCTTGGTACCTTCAACAGTGCTGAA
GAAGCTGCAAGAGCTTATGATGTTGAAGCACGAAGGATCCGTGGCAAGAA
GGCCAAGGTTAACTTTCCAGAGGAACCAACAGTTCCTCAGAAGCGCCGTG
CTTGCCCTGCTGCTCCTAAAGTTCCCAAGTCAAGCGCAGCACAGGAACCTA
CCGTCATACCAGCAGTCAACAACCTTGCCAACCCAAATGCTTTCGTCTACC
CGTCTGCTGACTTTGCATCAAAGCAGCCACTTGTTCAGCCTGATAACGTGC
CATTTGTTCCTGCAATTAACTCTGCTGCACCTGTTGAAGCTCCTGTTATGAA
TATGTACTCTGATCAGGGAAGCAACTCCTTTGGCTGCTCTGACTTGGGCTG
GGAGTATGACACCAAGACTCCAGATATATCATCCATTGCTCCCATTTCTACC
ATTGCTGAAGGAGCAGAAACTGCACTTCTCCAGAGTAACACCTACAACCC
AGCGGTGATTGCTGAAGGAGCTGAATCTGCGCCTGTCCAGACCAACACCT
ACAACTCAGTGGTGCCTCCTGTCATGGAGAACAATGCTGTTGATTTTGAAC
CTTGGATGAGGTTTCTTTTGGATGATGGCGTGGATGAGCCGATTGATAGCCT
TCTGAATTTTGATGTGCCTCAGGATGTCGTTGGCAACATGGACCTTTGGAG
CTTCGATGACATGCCCATCTGTGGCGAAATTTTCTGA

TaERFVII.8-6D

ATGTGCGGCGGCGCGATCCTCAAGGACCTCAAGGTCCCCGCGCCGACGCG
GAAGGTGACGGCGGCGGTGCTGTGGCCCGAGAAGAACAAGCCCAAGCGG
GCCGACGGCGTGGCCCGGCGCCTCGCGGGGCTCGGCCGGCGCGGGGGGC
TCGGGCTGGACGACGGCGACGTGGACTTCGAGGCCGACTTCGAGGAGTTC
GAGGCCGACTCCGGGGACTCCGACGTGGAGCTCGGGCGCGCCGGGGTTGC
TGGGAAGGACGGCCACGACGTGGTCGTCGAGATCAAGCCCTTATTCGCCG
TCAAGAGGTCCCTCTCCCAAGATGACTTAAGCACCATGACTACTGCTGGTT
TTGATGGTCCTGCACAAAGGTCAGCAAAAAGGAAGAGAAAGAACGAGTT
CAGGGGTATCCGCCAGCGCCCCTGGGGTAAGTGGGCTGCTGAAATCAGAG
ATCCTAGCAAGGGTGTCCGTGTCTGGCTTGGTACTTTCAACAGTGCTGAAG
AAGCTGCAAGAGCTTATGATGTTGAAGCACGAAGGATCCGTGGCAAGAAG
GCCAAGGTTAACTTTCCAGAGGAACTAACAGTTCATCAGAAGCGCCGTGC
TTGCCCTGCTGCTCCTAAAGTTCCCAAGTCAAGCGCAGCACAGGAACCTAC
CGTCATACCAGCAGTCAACAACCTTGCCAACCCAAATGCTTTCGTCTACCC
GCCTGCTGACTTTGCATCAAAGCAGCCACTTGTTCAGCCTGACAACGTGCC
ATATGTTCCCGCAATTAACTCTGCTGCCCCTGTTGAAGCTCCTGTTATGAAT
ATGTACTCTGACCAGGGAAGCAACTCCTTTGGCTGCTCTGACTTGGGCTGG
GAGTATGACACCAAGACTCCAGATATATCATCCATTGCTCCCATTTCCACCA
TTGCTGAAGGAACAGAGTCTGCACTTCTCCAGAGTAACACCTACAACCCA
GCAGTGATTGCCGAAGGAGCTGAATCTGCGCTTGTCCAGAGCAACACCTA
CAACTCAGTGGTGCCTCCTGTTATGGAGAACAATGCTGTCGATTTTGAGCC
TTGGATGAGGTTTCTTGTTGATGATGGCGTGGATGAGCCGATAGATAGCCTT
CTGAATTTTGATGTGCCTCAGGATGTCGTTGGCAACATGGACCTTTGGAGC
TTCGATGACATGCCCATCTGTGGCGAAATTTTCTGA

TaERFVII.9-7A

ATGTGCGGCGGCGCCATCCTCTCCGACATCATCCCGCCGCCGCGCCGGGCC
ACCGGCGGCAACGTCTGGCGGGCGGACAAGAAGAAGCGGGCCAGGGCCG
ATGCCGCCGCGGGGAGGCCCCGCCGCGCGCCCGAGGAGTTCGAGGAGGA
GGGCGACGCGGAGTTCGAGGCCGACTTCGAGGGGTTCGTGGAGGCGGAG
GAGGAGTCCGACGGCGAGGGCAAGCCCTTCCCCGTCCGCAGGAGCGGCTT
CTCCGGAGATGGATTGAAGGCAACTGCTGCTGGTGATGATGACTGTGCTTC
AGGGTCTGCTAAAAGGAAGAGAAAGAACCAGTTCAGGGGCATCCGCCGCC
GCCCTTGGGGTAAATGGGCTGCTGAAATAAGAGATCCTCGCAAGGGTGTCC
GTGTCTGGCTTGGCACTTACAACTCTGCCGAGGAAGCTGCCAGAGCCTATG
ATGTTGAAGCTCGCAGAATTCGTGGCAAGAAGGCAAAGGTCAATTTCCCA
GAAGAAGCTCCTATGGCTCCTCAGCAACGCTGCGCTACCTCTGTGAAGGTG
CCTGAGTTCAACACCGAACAGAAGCCAGTACTCAATACCATGGGCAACGC
AGATGTGTATTCCTGCCCTGCTGTTGACTACACCATAAATCAGCAATTTGTG
CAGCCTCAGAACATGTCGTTTGTGCCTACAGTGAATGCAGTTGAGGCCCCT
TTCATGAATTTTTCTTCTGACCAGGGGAGCAACTCCTTTAGTTGCTCAGACT
TCAGCTGGGAGAATGATATCAAGACCCCCGACATAACATCTGTGCCTGCAT
CCATTCCCACCTCAACAGAGGTCAATGAATCTGCATTTCTCCAGAACAACG
GCATTAATTCAACGGTACCTCCTGTGATGGGTGATGCTAATGTTGATCTTGC

CGACTTGGAGCCATACATGAAGTTCCTGATGGACGATGGTTCAGATGAGTC
AATTGACAGCATTCTAAGCTGTGATGTACCCCAGGACGTGGTCGGCAACAT
GGGCCTTTGGACCTTCGATGACATGCCCTTGTCTGCTGGTTTCTACTGA

TaERFVII.9-7B

ATGTGCGGCGGCGCCATCCTCTCCGACATCATCCCGCCGCCGCGCCGGGCC
ACCGGCGGCAACGTCTGGCGGGCGGACAAGAAGAGGAGGGCCAGGCCCG
ACGCCGCCGCGGGGAGGCCCCGCCGCGTGCCCGAGGAGGAGTTCCAGGA
GGAGGAGGGCGACGCGGAGTTCGAGGCCGACTTCGAGGGGTTCGTGGAG
GCGGAGGAGGAGTCCGACGGCGAGGCCAAGCCCTTCCCCGTCCGCAGGA
CCGGCTTCTCCGGAGATGGACTGAAGGCAACTGCTGCTGGTGATGATGACT
GTGCCTCAGGGTCTGCTAAAAGGAAGAGAAAGAGCCAGTTCAGGGGCATC
CGCCGCCGCCCTTGGGGTAAATGGGCTGCTGAAATAAGAGATCCTCGCAAG
GGTGTCCGTGTCTGGCTTGGCACTTACAACTCTGCTGAGGAAGCTGCCAGA
GCCTATGATGTTGAAGCCCGCAGAATTCGTGGCAAGAAGGCAAAGGTCAA
TTTCCCAGAAGAAGCTCCCATGGATCCTCAGCAACGCTGCGCTACCTCTGT
GAAGGTGCCCGAGTTCAACACCGAACAGAAGCCAGTACTCAACACCATGG
GCAACACAGATGTGTATTCCTGCCCTGCTGTTGACTACACCTTAAATCAGCA
ATTTGTGCAGCCTCAGAACATGTCGTTTGTGCCTACAGTGAATGCAGTTGA
GGCTCCTTTCATGAATTTTTCCTCTGACCAGGGGAGCAACTCCTTTAGTTGC
TCAGACTTCAGCTGGGAGAATGATATCAAGACCCCTGACATAACTTCTGTG
CTTGCATCCATTCCCACCTCAACTGAGGTCAATGAATCTGCATTTCTCCAGA
ACAATGGCATTAATTCAACGGTACCTCCTGTGATGGGTGATGCTAATGTTGA
TCTTGCCGACTTGGAGCCATACATGAAGTTCCTGATGGACGATGGTTCAGA
TGAGTCAATTGACAGCATTCTAAGCTGTGATGTACCGCAGGACGTTGTCGG
CAACATGGGCCTTTGGACCTTTGATGACATGCCCTTGTCTGCTGGTTTCTAC
TGA

TaERFVII.9-7D

ATGTGCGGCGGCGCCATCCTCTCCGACATCATCCCGCCGCCGCGCCGGGCC
ACCGGCGGCAACGTCTGGCGGGCGGACAAGAAGAGGCGGGCCAGGCCCG
ACGCCGCCGCGGGGAGGCCCCGCCGTGCGCCCGAGGAGGAGTTCCAGGA
GGAGGAGGGCGACGCGGAGTTCGAGGCCGACTTCGAGGGGTTCGTGGAG
GCGGAGGAGGAGTCCGACGGCGAGGCCAAGCCCTTCCCCGTCCGCAGGA
GCGGCTTCTCCGGAGATGGATTGAAGGCAACTGCTGCTGGTGATGATGACT
GTGCCTCAGGGTCTGCTAAAAGGAAGAGAAAGAACCAGTTCAGGGGCATC
CGCCGCCGCCCTTGGGGTAAATGGGCTGCTGAAATAAGAGATCCTCGCAAG
GGTGTCCGTGTCTGGCTTGGTACTTACAACTCCGCTGAGGAAGCTGCCAGA
GCCTATGATGTTGAAGCCCGCAGAATTCGTGGCAAGAAGGCAAAGGTCAA
TTTCCCAGAAGAAGCTCCTATGGCTCCTCAGCAACGCTGCGCTACTGCTGT
GAAGGTGCCCGAGTTCAACACCGAACAGAAGCCGGTACTCAACACCATGG
GCAACGCAGATGTGTATTCCTGCTCTGCTGTTGACTACACCTTAAATCAGCA
ATTTGTGCAGCCTCAGAACATGTCGTTTGTGCCTACAGTGAATGCAGTTGA
GGCCCCTTTCATGAATTTTTCCTCTGACCAGGGTAGCAACTCCTTTAGTTGC
TCAGACTTCAGCTGGGAGAATGATATCAAGACCCCTGACATAACTTCTGTG
CTTGCATCCATTCCCACCTCAACAGAGGTCAATGAATCTGCATTTCTCCAGA

ACAATGGCATCAATTCAACGGTACCTCCTGTGATGGGTGATGCTAATGTTGA
TCTTGCCGACTTGGAGCCATACATGAAGTTCCTGATGGACGATGGTTCAGA
TGAGTCAATTGACAGCATTCTAAGCTGTGATGTACCCCAGGATGTGGTCGG
CAACATGGGCCTTTGGACCTTTGATGACATGCCCTTGTCTGCTGGTTTCTAC
TGA

TaSAB18.1-5A

ATGGAAGGCAATAACCTGCCACCTGGAAACTTTATGCAAGGAGCAACTTAT
GGCAGTTCAGACTTGCACCGGAATCCCATGCAAATGCACGGTCCAAGCTCC
GGTAATCAGGGCTTCAACCACTCTCAGATACCTGGCAAATTCCCCATGCCTA
TGAACCAGGTTACAGATTCTGACCACTTGTCGGAATTTCAATTCAGAGGAC
AAAGGAAGGCTGATCACCACCAGGTCCACCACCACCACTATCACAAGAAG
GACTCCATGAGCGATGATGAGGAGCATGGTCTGAACGAGGATACCACTGAT
AGCCACAGCAGCAAGGGAAAGAAGGGCTCAGCATGGCAGCGGATGAAGT
GGACGGATTCAATGGTTAAGCTTTTAATTACTGCAGCGTCCTACACTGGTGA
GGATCCAGGAGCTGATTTAGGATGTGGAAGGAGGAACTGTGCAATGGTGC
ATAAAAAAGGCAAGTGGAAGGCAATATCAAAGGTGATAGGCGAGCGAGGT
TGCAATGTGTCACCGCAGCAGTGCGAGGATAAGTTCAATGACCTCAATAAG
AGATACAAAAGGCTTACAGATATCCTTGGTCGGGGTACAACTTGCAAGGTT
GTGGCGAATCCAGCACTTTTGGATCGCATGGATAATCTCTCTGACAAGTTG
AAAGATGACGCAAGGAAGATACTGAGCTCAAGGCACTTATTCTATGAGGAG
ATGTGCTCCTACCATAATAATAACCGGATTAGTTTGCCTGAAGATCTTCCACT
TCAGCGTTCACTGCAGTTTGCTCTTAGGTGCAAAGAGGAAAATGATATGAT
GAGAGGAGCAAGTGGAGATGCCGATGAAGATGACCAGAGTTCAGACTCTG
ATTATGAGGAAGATAATGATGAGGACCATCATGTGGCGCATAGCAATAAAG
GGGGCTTACCCATGCAAAAGAAGATGCGGTATACAGCGGATCACGAGGATG
CAGGTTTTGGGAACTCTTTGAGTGCACACGAATGTAGCCAGAGGTCTAATC
CCCATGGCATCGCACTAGATATCAACAAAGTTGTTCCAGATGGAACCAGCT
TGGCTTTGACACAGAAGGGCTTAGTATTGCAATCTGCAGAACTTGAAAAAC
AGTGCTTGAAAATCGAAAATGAGGCACTGGAGCTTGCAGAACAACGCCTC
AAGTGGGAACTATCCAGTAAAATTAAGGACAAGGAACTGGAAAGGATGAG
GTCAGATAACGAACATATGAAGATTGAGATTAAACGCTTAGAACTGGAGGT
AAGGCGCAAAGAGTTAGAGCTTGAACTCAAGCTGAAAGGAAATGTCAATC
ATTCATGA

TaSAB18.1-5B

ATGAGCGATGATGAGGAGCATGGTCTGAACGAGGATACCACTGATAGCCAC
AGCGGCAAGGGAAAGAAGGGCTCAACATGGCAGCGGATGAAGTGGACCG
ATTCAATGGTTAAGCTTTTAGTTACTTTAGCGTCCTACACTGGTGAGGATCC
AGGAGCTGATTTAGGATGTGGAAGGAGGAACTGTGCAATGGTGCATAAGA
AAGGCAAGTGGAAGGCAATATCAAAGGTGATGGGCGAGCGAGGTTGCAAT
GTGTCACCGCAGCAGTGCGAGGATAAGTTCAATGACCTCAATAAGAGATAC
AAAAGGCTTACAGATATCCTTGGTCGGGGTAGAACTTGCAAGGTTGTGGAG
AATCCAGCACTTCTGGATCGCATGGATAATCTCTCTGACAAGTTGAAAGAT
GACGCAAGGAAGACACTGAGCTCAAGGCACTTATTCTATGAGGAGATGTG
CTCCTACCATAATAATAACCGGATTAGTTTGCCTGAAGATCCTGCACTTCAG

CATTCACTGCAGTTTGCTCTTAGGTGCAAAGAGGAAAATGATATGATGCGA
GGAGCAAGTGGAGATGCCGATGAAGATGACCAGAGTTCAGACTCTGATTAT
GAGGAAGATAATGATGAGGACCATCATGTGGCACATAGCAATAAAGGGGGC
TTACCCATGCAAAAGAAGATGCGGTATACAGCAGATCACGAGGATGCTAGT
TTTGGGAACTCTTTGAGTGCACACGAATGTAGCCGGAGGACTAATCCCCAT
GGAATCGCACTAGATATCAACGAAGTTGTTCCAGATGGAACGAGCTTGGCT
TTGACATAG

TaSAB18.1-5D

ATGGAAGGCAATAACCTGCCACCTGGAAACTTTATGCAAGGAGCAACTTAT
GGCAGTTCAGACTTGCACCGGAATCCCATGCAAATGCACGGTCCAAGCTCC
GATAATCCGGGCTTCAACCACTCTCAGATACCTGGCAAATTCCCCATGCCTA
TGAACCAGGTTACAGCTTCTGACCACTTGTCGGAATTTCAATTCAGAGAAC
AAAGGAAGGCTGATCACCACCAGGTCCACCACCACCACTATCCCAAGAAG
GACTCCATGAGCGATGATGAGGAGCATGGTCTGAACGAGGATACCACTGAT
AGCCACAGCGGCAAGGGAAAGAAGGGCTCAGCATGGCAGCGGATGAAGT
GGACTGATTCAATGGTTAAGCTTTTAATTACTGCAGCGTCCTACACTGGTGA
GGATCCAGGAGCTGATTTAGGATGTGGAAGGAGGAACTGTGCAATGGTGC
ATAAGAAAGGCAAGTGGAAGGCAATATCAAAGGTGATGGGCGAGCGAGGT
TGCAATGTGTCACCGCAGCAGTGCGAGGATAAGTTCAATGACCTCAATAAG
AGATACAAAAGGCTTACAGATATCCTTGGTCGGGGTACAACTTGCAAGGTT
GTGGAGAATCCAGCACTTCTGGATCGCATGGATAATCTCTCTGACAAGTTG
AAAGATGACGCAAGGAAGATACTGAGCTCAAGGCACTTATTCTATGAGGAG
ATGTGCTCCTACCATAATAATAACCGGATTAGTTTGCCTGAAGATCCTGCAC
TTCAGCGTTCACTGCAGTTTGCTCTTAGGTGCAAAGAGGAAAATGATATGA
TGAGAGGAGCAAGTGGAGATGCCGATGAAGATGACCAGAGTTCAGACTCT
GATTATGAGGAAGATAATGATGAGGACCATCAGGTGGCGCATAGCAATAAA
GGGGGCTTACCCATGCAAAAGAAGATGTGGTATACAGCGGATCACGAGGAT
GCTGGTTTTGGGAACTCTTTGAGTGCACACGAATGTAGCCGGAGGTCTAAT
CCCCATGGCATCACACTAGATATCAACAAAGTTGTTCCAGATGGAACCAGC
TTGGCTTTGACACAGAAGGACTTAGTATTGCAATCTGCAGAGCTTGAAAAA
CAGTGCTTGAAAATCGAAAATGAGGCACTGGAGCTTGCAGAACAACGCCT
CAAGTGGGAACTATCCAGTAAAATTAAGGACAAGGAACTGGAAAGGATGA
GGTCAGATAACGAACATATGAAGATTGAGATTAAACGCTTAAAACTGGAGG
TAAGGCGCAAAGAGTTAGAGCTTGAACTCAAGCTGAAAGGAAACGGCAG
TCATTCATGA

TaSAB18.2-1A

ATGGACCGCAGTAACCTACCTCCTGGAAACACGACGCAAGGAACTCCTTAT
GCTAGTTTGAATCTACATGGTAACTCCTTGCAAATGCATGCTCCAAGCTCAG
GAAAACATCTATTTAACCAATCTCAGATGCCAGGGAGTTTCACAATGCCTAT
CAACCGGGCTACAGAACATGATAACCCCGGATCCGGATTTCAGTTTGTAGA
ACATGGAAAAAAGGATCACCACCAGCAGCAGCAGCAGCACAACCATCTCA
TAAAGAACTCCATCAGTGACGACGAGGAGCATGATATGACTGAGGATGCTA
CTGATGCCCAGAGTGGCAAGGGCAAGAAAGGCTCAGCATGGCATCGGATG
AAGTGGACTGGTTCGATGGTTAAGCTTTTGATTACCGCAGCATCTTACACG

GGGGAGGATCCTGGGGCGGATTTGGGCGGCGGGAGGAGGAACATTACAGT
AATGCAGAAGAAGGGCAAGTGGAAAGCAATATCAAAGGTCATGGGCGAAA
GGGGCTGCAATGTGTCACCGCAGCAGTGCGAGGATAAGTTCAATGACCTC
AACAAGAGATACAAAAGGCTTACGGATATCCTTGGTCGTGGTACTGCTTGC
AATGTTGTGGATAATCCAGCACTCCTTGATTGCATGAATAATCTTTCCGATAA
GATGAAAGAAGATGCAAGGAAGATATTGAACTCTAAGCATTTATTCTATGA
GATGATGTGTTCCTATCATAACAACAACCGTGTGAATTTACCCGAAGATCTT
GCACTTCAGCACTCACTACAGGTTGCTCTTAGATGTAAAGAGGAGCATGAT
CCAAGGAGGGATGCAAGTGGAGATGCTGAAGAAGATGACCATAGTGCAGA
TTCTGATTACGAGGATCATGACGAAGAGCATCAAGCAGTTCATCACAGTAT
GAGGGATCCTTCCATGAATAAAAGGATGTGTCATGCATTGGATCATGGTGAT
GCAGGTTTTCTCACCTCAAGCTCGAATGATGGTAGTGGGAGTTTGGATCCC
CATGGCATCGCATTGGATATCAACAATGGTTTTACGGATGGAACCAACCTGT
CTGTTGTGCGGAAGGAACTGGCTTCCCAAGCAATAGAGCTTCGGAAACGT
CGCTTGCAGATTGAAGCACAGGAACTGGAAGTAACAGAGCAACGTCTAAA
GTGGGAGAGATTCAAGAGGAAGAAGGACAGGGAAATTGAAAGGATGGAA
TCGGAGAATGAAGAAATGATGCTCGAGAACAAGCGTTTGGAACTTCAGCT
AAAGCACAAGGAGCTAGAAGTTGAGCTTAAGCTAAAAGGCAATCCAGACC
ATGAATGA

TaSAB18.2-1B

ATGGACCGCAGTAACCTACCTCCTGGAAACACGACGCAAGGAGCTCCTTAT
GGTAGTTTGAATGTACATGGTAACTCCATGCAAATGCATCCTCCAAGCTCAG
GAAAACATCTATTCAACCAATCTCAGATGCCAGGGAGTTTCACAATGCCTAT
CAACCGGGCTACAGAGCATGATAACCCCGGATCCGGATTTCAGTTTGTAGA
ACATGGAAAAAAGGATCACCACCAGCAGCAGCAGCAGCACAACCATCTTA
TAAAGAACTCCATCAGTGACGACGAGGAGCATGATATGACCGAGGATACTA
CTGATGCCCAGAGTGGCAAGGGCAAAAAAGGCTCGGCATGGCATCGGATG
AAGTGGACTGGTTCAATGGTTAAGCTTTTGATTACCGCAGCGTCTTACACG
GGGGAGGATCCTGGGGCGGATTTGGGCGGCGGGAGGAGGAACATTACAGT
AATGCAGAAGAAGGGCAAATGGAAAGCAATATCAAAGGTTATGGGTGAAA
GAGGCTGCAACGTGTCACCGCAGCAGTGCGAGGATAAGTTCAATGACCTC
AACAAGAGATACAAAAGGCTTACGGATATCCTTGGTCGTGGTACTGCTTGC
AATGTTGTGGATAATCCAGCACTCCTTGATTGCATGAATAATCTGTCTGATA
AGATGAAAGAAGATGCAAGGAAGATATTGAACTCTAAGCATTTATTCTATG
AGATGATGTGTTCCTATCATAACAACAACCGTGTGAATTTACCCGAAGATCC
TGCACTTCAGCACTCACTACAGGTTGCTCTTAGATGTAAAGAGGAGCATGA
TCCGAGGAGGGATGCAAGTGGAGATGCTGAAGAAGATGACCACAGTGCAG
ATTCTGATTATGAGGATCATGACGAAGAGCATCAAGCAGTTCATCACAGTAT
GAGGGATCCTCCCATGAATAAAAGGATGTGTCATGCATTGGATCATGGTGAT
GTAGGTTTTCTCACCTCAAGCTCGAATGATGGCAGTGGGAGTTTGGATCCC
CATGGCATCGCATTGGATATCAACAAAGTTTTTCCGGATGGAACCAACCTGT
CTGTTGTGCGCAAGGAACTGGCTTCCCAAGCAATAGAGCTTCGGAAACGT
CAGTTGCAGATTGAAGCACAGGAACTGGAAGTAACAGAGCAACGTCTAAA
GTGGGAGAGATTCAAGAGGAAGAAGGACAGGGAAATTGAAAGGATGGAA

TCGGAGAATGAAGAAATGATGCTCGAGAACAAGCGTTTGGAACTTCAGCT
AAAGCACAAGGAGCTAGAAGTTGAGCTTAAGCTAAAAGGCAATCCAGACC
ATGAATGA

TaSAB18.2-1D

ATGGACCGCAGTAACCTACCTCCTGGAAACACGACGCAAGGAACTCCTTAT
GGTAGTTTGAATGTACATGGTAACTCCATGCAAATGCATGCTCCAAGCTCAG
GAAAACATCTATTCAGCCAATCTCAGATGCCAGGGAGTTTCACAATGCCTAT
CAACCGGGCTACAGAACATGATAACCCCGGATCCGGATTTCAGTTTGTAGA
ACATGGAAAGAAGGATCACCACCACCACCAGCAGCAGCAGCAGCAGCAG
CAGCAGCAGCAGCACAACCATCTCATAAAGAACTCCATCAGTGATGACGA
GGAGCATGATATGACCGAGGATGCTACTGATGCCCAGAGTGGCAAGGGCA
AGAAAGGCTCAGCATGGCATCGGATGAAGTGGACTGGTTCGATGGTTAAG
CTTTTGATTACCGCAGCATCTTACACGGGGGAGGATCCTGGGGCGGATTTG
GGCGGTGGGAGGAGGAACATTACAGTAATGCAGAAGAAGGGCAAATGGA
AAGCAATATCAAAGGTCATGGGTGAAAGAGGCTGCAACGTGTCACCGCAG
CAGTGCGAGGATAAGTTCAATGACCTCAACAAGAGATACAAAAGGCTTAC
GGATATCCTTGGTCGTGGTACTGCTTGCAATGTTGTGGATAATCCAGCACTC
CTTGATTGCATGAATAATCTTTCCGATAAGATGAAAGAAGATGCAAGAAAG
ATATTGAACTCTAAGCATTTGTTCTATGAGATGATGTGTTCCTATCATAACAA
CAACCGTGTGAATTTACCCGAAGATCTTGCACTTCAGCACTCACTACAGGT
TGCTCTTAGATGTAAAGAGGAGCATGATCAAAGGAGGGATGCAAGTGGAG
ATGCTGAAGAAGATGACCACAGTGCAGATTCTGATTACGAGGATCATGACG
AAGAGCATCAAGCAGTTCATCACAGTATGAGGGATCCTTCCATGAATAAAA
GGATGTGTCATGCATTGGATCATGGTGATGCGGGTTTTCTCACCTCAAGCTC
GAATGATGGTAGTGGGAGTTTGGATCCCCATGGCATCGCATTGGATATCAAC
AAGGGTTTTACGGATGGAACCAACCTGTCTGTTGTGCGGAAGGAACTGGC
TTCCCAAGCAATAGAGCTTCGGAAACGTCGGTTGCAGATTGAAGCACAGG
AACTGGAAGTCACAGAGCAACGTCTAAAGTGGGAGAGATTCAAGAGGAA
GAAGGACAGGGAAATTGAAAGGATGGAATCGGAGAATGAAGAAATGATGC
TCGAGAACAAGCGTTTGGAACTTCAGCTAAAGCACAAGGAGCTAGAAGTT
GAGCTTAAGCTAAAAGGCAATCCAGACCATGAATGA

Os09g0287000

ATGTGTGGAGGAGCACTGATCCCGAACGACTATGGCGACAAGCCGCCGCC
GCCGCCGTCGGAGTCGTCGGAGTGGGACGCCACAACGAAGATGAAGAAG
AAGAAGAAGCGTGGTGGCGGCGGCGACGACGACTGGGAGGCCGCCTTCC
GGGAGTTCATCGCTGGCGACGACGACGACGACGACGGCGGCGTTTCCATG
TTCCCTTCTGGTGCAGGGACGATGGAGACGACCACAGAGGTGGCGCCGGC
GGCGGCGGTGGTGGAGAGGCCGCGGCGGCGGCGAAGGGTGAGGCGGAGC
TACCCGTACCGCGGCGTCCGGCAGCGGCCGTGGGGGCGGTGGGCGTCGGA
GATCCGCGACCCCGTCAAGGGCGCCCGCGTCTGGCTCGGCACCTTCGACA
CCGCCGTCGAGGCCGCGCGCGCCTACGACGCCGAGGCGCGCCGCATCCAC
GGCCACAAGGCAAGGACCAACTTCCCGCCCGACGAGCCTCCGCTGCCGGC
GCCATCGCAGGCGCCGTTCTGCTTCCTGCTCGACGACGACGACGACGACG
ACGGCGTGGCCCGTGGAAACAGCCCGGCGTCGTCGTCGGCGCCGGACAG

AGCCTCCGCTTGCACGACGTCGTCGACGGTGGCGTCCGGCGAGCGAGGCG
ATGAGCTCATACTGCTGGAGTGCTGCTCCGACGACGTGATGGACAGCCTCC
TCGCCGGCTTCGACGTGTCCAGCGAACCACGCAGTGTTTTGGGAATGGTTA
ATTAG
Os03g0182800
ATGTGCGGCGGTGCAATCCTCGCCGATTTCACCCCGGCGAGGGTGCCCCGG
CGGCTGACCGCCGCCGAGCTCCTGCCGGTGACCCCGACTCCCCCCGCCGC
CGAGAGGAGAACCACCCGGAAGCGCAAGTCCGACGTCGACTTCGAGGCG
GAGTTCGAGCTTTTCGAGGACGACGACGACGACGATGAGTTCGAGCTTTC
CGACGATGGCGACGAGAGTTTGGCCGTGTCATGTGTGTCGTCCCCCAAGTC
GAAGGCAGTACCTTCGTTTTCTTTTTCGTCGGATGTCTCCTCGAGCTCCAGG
CCGCGGCGGCGCGTGGCGGCGGCGGCGGCCGGTCGTCGGAAGGCGAGCA
AGAAGAGCAAGTACAGGGGCGTCCGGCGCCGGCCGTCGGGGAGGTTCGC
GGCGGAGATCAGGGACCCCAAGAAGGGGCGGCGCGTGTGGCTCGGCACG
TACGGCAGCGCCGAGGAGGCCGCCATGGCCTACGACCGCGAGGCCCGCCG
CATCCGCGGCAAGGGCGCGAGGCTCAACTTCCCCCGCGACGGCGATGGCT
CCCCTCGCCGGAGTAACGACCGGCCCTGCTGGACCATCGACCTCAACCTCC
CCGCGGCGGCCGTCTCCGGTGACGACGACGACGCCATGGCCGTCGACGCC
GCAGACGCAGACGCAGGCAGTGCTGGCCGTGCAGCAGCCTATGCAGATCA
AGAAGCACTGAGCGCGGCAAAGTGCAAGATCAAGCAGTGTCCTCGCGAC
GAACAGATGGCGAGCGCCACACCTGAGCTCATGGAGGAGGACGCGAGCA
GCAGCAGAAACATGGTGCCCCTGTCCATGGCGCTGCAGCTGCAGTATGCGG
CGATGATCGCCGAATGCGACCGCGAGATGGAGGAGATCGCCGCCGTGGAG
AGGGACCTCGAGAGGCGCAGGAGGCAGGTGTTCGAGCGCAGAGGCCACC
TGGTCAGGCAGGCCTCTCTTCTGCTCGACTGA
Os03g0341000
ATGTGCGGCGGCGCCATCCCGCTGATCAGCAGCCGCGGCCCCGGCGGCAA
GAGGAGCCTCTCCGCCGCCGATGAGCTCTGGCCGCCGCCGCCGCAGCACG
CCAGCGACGACCCGGCCGAGCAAGCGGCGGCGGATGAGGAGGAGCAGGA
GCAGCAGCCGGCGGCGAGGAGGCAGCGGCGAGGGGAGCGGAGGACGCT
GTACCGGGGCATCCGGCGCAGGCCGTGGGGGAAATGGGCGGCGGAGATCC
GCGACCCGGCCAAGGGCGCCCGCGTCTGGCTCGGCACCTTCGCCACCGCC
GAGGCTGCCGCGCGGGCCTACGACCGCGCCGCCCGCCGCATCCGCGGCAC
CAAGGCCAAGGTCAACTTCCCCAACGAGGACAACGCCTTCGCCGCCGCGC
CGCCGCCGTACCACCTCGCCGCCTACTACGGCGACGCCTCCTCCACCTCCT
ACCTCTACCCGATGGCCATGACGCCCGCCGCCGCCGGACTGAGGGAGCAG
CAGCTGATGACGACGACGGCGGTGGAGTACAGCGTTAATGACGCCGTCGA
CGTGGCCAGCGTTTACTTCCAGCCGCCGCCGCCGGCGGTTGCTTACGAGTT
CAGCGCCGTCGGCGGTGGCGCCGTCGTCGTGCCGGTGTCGGCGGTGGCGC
CGGCGATGACGTACGGACAGAGCCAAGAGGTGGCGGCTCCGCTCATGTGG
AATTTCGATGACATCACGGCCATGCCAATGTGA
Os07g0674800
ATGTGTGGCGGCGCGATCATTTCCGACTTCATCCCGCAGCGGGAAGCCCAC
CGCGCGGCCACCGGCAGCAAGCGTGCCCTCTGCGCCTCCGACTTCTGGCC

GTCGGCGTCGCAGGAAGCCGCCGACTTCGACCACCTCACCGCCCCCTGCA
CCTTCACCCCCGACCAAGCGGCAGAGGAGCCGACCAAGAAGCGGGAGCG
GAAGACGCTGTACCGTGGCATCAGGCGGCGGCCGTGGGGGAAGTGGGCG
GCGGAGATCCGCGACCCGGCGAAGGGCGCGCGTCTGGCTCGGCACCTT
CGCCACCGCCGAGGCGGCGGCCCGCGCCTACGACCGCGCCGCCCGCCGCA
TCCGCGGGGCCAAGGCCAAGGTCAACTTCCCCAACGAGGACCCGCCACTC
GACGACCCGGCCGCCGACGGCCACAGCCACGGCGGCGCCGCCATCCCGTG
CAGGGAGTTCATGGACTACGACGCCGTCATGGCGGGCTTCTTCCACCAGCC
CTACGTCGTCGCCGACGGCGTGCCGGCCGTGCCGGCGGAGGAGGCGCCCA
CGGTGGCGTACGTGCACCACCACCTGCCGCCGCAGCCGCAGCAGGACGCG
GGGCTGGAGCTCTGGAGCTTTGATAACATCCACACGGCCGTGCCGATGTGA

Os01g0313300

ATGTGCGGCGGTGCAATCATCTACGACTACATCCCGGCGCGCCGCCGGTTG
TGCGCCTCCGACTTCTGGCCCGACGCCGACGACTCCGACCCCCACACCCC
CGCTCCCGAGAAACCGCCGCGCGCGAAGAGGGAGCGGAAGAACCAGTAC
CGCGGGATCAGGCAGCGGCCGTGGGGGAAGTGGGCGGCGGAGATCCGCG
ACCCGGTGAAGGGGGTGCGCGTCTGGCTCGGCACCTACCCGACCGCCGAG
GCCGCCGCGCGGGCCTACGACCGCGCCGCGCGCCGCATCAGGGGCGCCAA
GGCGAAGGTCAACTTCCCCAACGACTTCGGCGCCGCCCCCGCGCCGGCCG
CGGCGGCGGCGAAGGCCGTCCCTCGCGTCGCGCCCACGCCGGCCGTGCTC
CCGCCGCCCAAGATGGAGGCGGTGTCCGAGGGCGCCGGCGCCTGCTCCTC
CGACGAGGTCAAGGAGCTGTCCGAGGAGCTGCTCGCGTACGAGAACTACA
TGAGCTTCCTCGGCATCCCCTACATGGAGGGCGGCGCCGCCTCCGCCGCCG
GCGCCGAGGAAGCCGCGGCGCCCGCCGGGCTCTGGACCTTCGAAGACTAC
GAGCTGCCGTCGCTAGCGCTCTAG

Os05g0361700

ATGTGTGGGGGAGCGATCATCGCCGACTTCGTCCCGCCCGCCGGCGCCCGC
CGCGCCGCTGCCTCCGACATCTCCGACAACGCCGTCCTCTCCGCTGCCGGT
GCCGGTGACGAGTCGTTCGCGGCGGCCAAGGCGCCGGCGCCGGGGAGGA
AGACGGCGTACCGCGGCATCCGGCGCCGGCCGTGGGGACGCTGGGCGGCG
GAGATCCGCGACCCGAGGAAGGGCGCCCGCGTCTGGCTCGGCACCTACGC
CACCGCCGAGGAGGCCGCCCGCGCCTACGACGTCGCGGCGCGCGACATCC
GCGGCGCCAAGGCCAAGCTCAACTTCCCCCCGACCATCGGCGCCGCCGCC
GCGCCACCGCCGCCCAAGAAGCGACGCAAAGCCGCCGCCGCGGCGAACC
ACCACCACCACCACCACCAGCAGGAGAGCTCAGGCTCCTCGTCGGCGTCG
TCGCTGCCTCCCACCCCGCCGCCGGCCGCCGAGCACCAGCTCCGCGAGTG
CATGTCCGGGCTGGAGGCGTTCTTGGGCCTCGAGGAGGAGGAGGACGACG
GCGGCGCCGGTGAGCCATGGGACGCCGTCGACATGATGCTCGAGTAG

Os03g0183000

ATGTGTGGAGGCGCCATCCTCGCCGAGTTCATCCCGGCGCCGTCGCGCGCC
GCGGCGGCGACCAAGCGGGTGACCGCCAGCCACCTGTGGCCGGCCGGCTC
CAAGAACGCCGCCCGCGGCAAGAGCAAGAGCAAGAGGCAGCAGAGGAG
CTTCGCCGACGTCGACGACTTCGAGGCCGCCTTCGAGCAGTTCGACGATG
ACTCCGACTTCGACGACGCGGAGGAAGAAGACGAAGGACACTTCGTGTTC

GCGTCCAAATCTCGTGTCGTCGCCGGGCACGACGGGCGCGGCGGCGAG
GGCGGCGAGCAAGAAGAAGCGGGGGCGGCACTTCCGAGGCATCCGGCAG
CGGCCATGGGGGAAGTGGGCGGCGGAGATCCGCGACCCGCACAAGGGCA
CGCGCGTCTGGCTCGGCACGTTCAACACCCCGGAGGAGGCCGCACGCGCC
TACGACGTCGAGGCGCGCCGCCTCCGCGGCAGCAAGGCCAAGGTCAACTT
CCCCGCCACGCCCGCCGCCGCGCGCCCACGCCGCGGCAACACGAGAGCCA
CCGCCGTGCCACCGCCGGCGACAGCACCCGCCGCCGCCCCGCCGCGCGGA
CTGAAGCGAGAATTCTCGCCGCCTGCTGAGACCGCGCTACCTTTCTTCACC
AACGGCTTCGTCGACCTGACGACCGCCGCGGCGCCGCCACCGGCCATGAT
GATGACGAGCTCCTTCACCGACAGCGTCGCCACGTCGGAGTCCGGCGGGA
GCCCCGCCAAGAAGGCGAGGTCCGACGACGTCGACTCGTCCGAGGGCAG
CGTCGGCGGCGGCAGCGACACGCTGGGTTTCACCGACGAGCTGGAGTTCG
ACCCGTTCATGCTGTTCCAGCTCCCCTACTCCGACGGCTACGAGTCCATCG
ACAGCCTCTTCGCCGCCGGCGACGCCAACAGCGCGAACACCGACATGAAC
GCCGGCGTCAACCTGTGGAGCTTCGACGACTTCCCAATCGACGGCGCCCTT
TTCTGA

Os07g0617000

ATGTGTGGAGGATCCATTCTCGGCGACCTTCACTTGCCGGTGCGGCGGACA
GTGAACGCCGGTGACCTGTGGGGAGACGCCGGCAAGGGTAGAGATGGTG
GCGACGGCTTGAAGAAGAGGAAGGGGAGTTCTTGGGATTTCGATGTTGAT
TGCGATGATGATGATGATGATGATGACTTTGAGGCTGATTTTGAGGAGTTTGAGG
ATGACTATGGCGATGATGATGATGATGTGGGTTTCGGGGACGACGACCAAGAAT
CCGACATGAACGGTCTCAAGCTCGCCGGATTCAGCACCACGAAGCTCGGC
CTCGGCGGCAGCAGGAAGAGGAAGACGCGATACCGAGGGATCCGGCAGC
GGCCATGGGGGAAATGGGCGGCGGAGATCAGGGACCCCCGCAAGGGCGT
CCGCGTCTGGCTCGGCACGTTCGGCACCGCCGAGGAGGCCGCCATGGCGT
ACGACGTCGAGGCACGCCGCATCCGCGGCAAGAAAGCCAAGGTCAACTTC
CCCGACGCCGCCGCCGCCGCCCCGAAGCGGCCACGGCGTTCTTCGGCGAA
GCATTCGCCGCAGCAGCAGAAGGCCAGGTCGTCGTCGTCGTCGCCGGCGA
GCCTGAACGCCAGCGACGCCGTGTCCAAGTCCAACAACAACCGCGTCAGC
TCGGCTGGGAGCAGCACCGACGCCACCGCCGCCGCCATCGCCATCGACGA
CGGCGTCAAGCTCGAGCTGCTCTCGGAGACGGATCCTTCTCCGCCCATGGC
CGCCGCCGCCGCCGCGTGGCTCGACGCGTTCGAGCTGAACGATCTTGACG
GATCAAGATGCAAGGACAACGCATTCGATCACCAGATTCACAAGGTAGAA
GCGGCTGTCGCTGATGAATTCGCGTTCTACGACGATCCGAGCTACATGCAG
CTGGGTTACCAGCTCGATCAGGGCAACTCGTACGAGAACATCGACGCGCTC
TTCGGCGGCGAGGCCGTCAACATTGGTGGACTCTGGAGCTTCGACGACAT
GCCAATGGAGTTCAGAGCTTATTGA

Os09g0434500

ATGTGCGGCGGAGCAATCATCTCCGGGTTCATCCCGCCGTCGGCCGCTGCG
GCGGCGGCGGCTGCGGTGGCCAAGAAGCAGCAGGGCAGGAGGGTCACGG
CCGACGTGCTGTGGCCGGGGATGCTGCGGAAGGGGAAGGCGGCGGCGGC
GGAGGAGGACTTTGAGGCCGACTTCCGCGAGTTCGAGCGTGGCATGAGCG
ACGACGAGGCGGAGGGGGGCGGCGGCGAGGAGGAGGAGGACGACGACG

ACGTGGTCGTGGTGGTCCCCCCGCCGGCGGCGGCGAGGTTCGTCGTCCGT
GCCGCGGCCAAGGCGGCGCCCCCAACTGCAGATGGGATGTTGACTACAAA
GCTTGTCCAACATGATGGACCTACTGCTAGATCAGCAAAGCACAAGAGGA
AGAATCAGTACAGGGGGATCCGCCAGCGTCCCTGGGGCAAATGGGCAGCT
GAAATCCGAGACCCCAGCAAGGGTGTCCGTGTTTGGCTTGGAACATATAAC
ACTGCTGAGGAGGCAGCTAGGGCATATGACGCTGAAGCCCGCAAGATCCG
TGGCAAGAAAGCCAAGGTCAACTTTCCTGATGAACCAGCTGTTGCTCAGA
AGCTCTCCCTGAAGCAAAACGCTGCCAAGCAAGAGAAACTAGCTCCACCT
CTGAAGACCTGTGGCGATGATGCTTTCTTTCAGCTAAACAGTTCAGACAAT
GATTTGTTTGCAATGCTTGCAAAGGTGCCTGCAAAGCCGGCAGAGCCTGTT
GATCTCATGCCTCCAGTCAAACCTCTTGCTTCCACTGAGACATTCGAGATG
AACATGCTCTCTGATACGAGCAGCAACTCATTTGGCTCTTCAGACTTTGGTT
GGGAGGATGACACCCTGACCCCAGACTACACTTCAGTCTTCGTTCCTAATG
CTGCCATGCCAGCATATGGTGAACCTGCTTACCTGACAGGTGGAGCGCCAA
AGAGAATGAGGAACAACTATGGTATCGCCGTGCCCCAGGGAAATGGCATG
CCTAATCTCGCACAAAACATGCCCACCTTCGATCCCGAGATGAAGTATTTGC
CATTACCTTATGTTGAGAGCAGCTCAGATGAATCAATGGACAACCTTCTGC
AAAATGATGCTACACAAGACGGGGCAAGCAACGAGGGCATCTGGAGCCTT
GATGAGCTGCTCATGGCAGCTGGTGCCTACTGA
Os06g0194000
ATGTGCGGCGGCGCCATCCTCTCCGACCTCATCCCGCCGCCGCGGCGGGTC
ACCGCCGGCGACCTCTGGCTGGAGAAGACCAAGAAGCAGCAGCAGCAGA
AGAAGAAGAACAAGGGCGCGAGGAGGCTGCCACTGCGCCAAGAGGAGG
AGGATGATTTCGAGGCCGACTTCGAGGAGTTCGAGGTGGATTCCGGCGAG
TGGGAGGTGGAGTCCGACGCCGACGAGGCCAAGCCGCTCGCCGCGCCCC
GGAGCGGCTTCGCTAAAGGTGGATTGAAAAACACTACTGTTGCTGGTGCT
GATGGGCCTGCAGCAAGGTCTGCTAAAAGGAAGAGAAAGAACCAATTCAG
GGGTATCCGCCAGCGGCCATGGGGCAAATGGGCTGCGGAAATCAGAGATC
CTCGCAAAGGTGTCCGCGTCTGGCTTGGCACCTTCAACTCTCCTGAGGAA
GCTGCCAGAGCTTATGATGCTGAAGCACGAAGGATTCGAGGCAAGAAGGC
CAAGGTCAATTTCCCAGATGGGGCTCCAGTGGCTTCTCAGAGGAGTCATGC
TGAGCCCTCCTCCATGAACATGCCTGCTTTCAGCATCGAAGAGAAGCCGGC
CGTCATGTCAGCAGGCAACAAAACCATGTACAACACAAATGCTTATGCCTA
CCCTGCTGTTGAGTACACCTTACAGGAGCCATTTGTGCAGATTCAGAATGT
CTCATTTGTTCCTGCAATGAACGCGATTGAGGATACTTTCGTGAACCTGTCC
TCTGATCAAGGGAGCAACTCCTTTGGTTGCTCGGACTTTAGCCAGGAGAAT
GATATCAAGACCCCTGACATAACTTCCATGCTTGCACCGACCATGACAGGT
GTTGATGACTCCGCATTCCTCCAGAACAATGCCAGTGATGCAATGGTACCT
CCTGTGATGGGGAATGCTAGCATTGATCTTGCTGACCTGGAGCCGTACATG
AAATTTCTGATCGATGGTGGTTCGGATGAGTCGATTGACACCCTTCTGAGCT
CTGATGGATCTCAGGATGTGGCCAGTAGCATGGACCTTTGGAGCTTCGATG
ACATGCCCGTGTCGGCCGAGTTCTACTGA
Os02g0782700
ATGTGCGGCGGCGCCATCATCCACCACCTGAAGGGGCACCCGGAGGGGTC

GCGCCGGGCGACGGAGGGGCTCCTGTGGCCCGAGAAGAAGAAGCCCAGG
TGGGGCGGCGGCGGGAGGCGCCACTTCGGGGGGTTCGTGGAGGAGGACG
ACGAGGACTTCGAGGCCGACTTCGAGGAGTTCGAGGTGGACTCCGGGGAC
TCGGATTTGGAGCTCGGGGAGGAGGACGACGATGACGTCGTCGAGATCAA
GCCGGCCGCCTTCAAGAGGGCCCTCTCCAGAGATAACTTGAGCACCATTAC
CACTGCCGGATTTGATGGTCCTGCTGCAAAGTCTGCCAAAAGAAAGAGAA
AGAACCAATTCAGGGGCATCCGCCAGCGCCCTTGGGGTAAGTGGGCTGCT
GAAATCAGAGATCCTCGCAAGGGTGTTCGTGTCTGGCTTGGCACTTTCAAC
AGTGCTGAAGAAGCTGCAAGAGCTTATGATGCTGAAGCACGCAGGATTCG
TGGCAAGAAGGCCAAGGTGAATTTTCCAGAGGCTCCAACAACTGCTCAGA
AGCGTCGTGCTGGCTCCACCACTGCTAAAGCACCCAAGTCAAGTGTGGAA
CAGAAGCCTACTGTCAAACCAGCATTCAACAATCTTGCCAATGCAAATGCG
TTTGTCTACCCATCTGCTAACTTCACTTCAAACAAGCCGTTTGTTCAGCCTG
ATAACATGCCATTTGTTCCTGCAATGAACTCTGCTGCTCCTATTGAGGACCC
TATCATCAACTCTGACCAGGGAAGCAACTCATTTGGCTGCTCTGACTTTGG
CTGGGAGAATGATACCAAGACACCAGATATTACATCAATTGCTCCCATTTCA
ACCATAGCTGAAGTCGATGAATCTGCATTCATTAAGAGCAGTACCAACCCA
ATGGTCCCTCCTGTTATGGAGAACAGTGCTGTTGATCTGCCTGATTTAGAAC
CCTACATGAGGTTCCTTCTGGATGATGGTGCTGGTGACTCAATTGATAGCCT
TCTCAACCTGGATGGATCACAGGATGTTGTCAGCAACATGGACCTCTGGAG
CTTTGATGACATGCCCGTTAGCGATTTCTATTGA
Os03g0183300
ATGTGCGGCGGAGCGATCCTTGCGGAGCTCATACCGAGCGCGCCGGCGGC
GAGGCGCGTCACGGCGGGCCACGTCTGGCCGGGCGACGCCAACAAGGCC
AAGAAGAAGGGCGCGCGCGCCGACGACTTCGAGGCCGCGTTCCGCGACTT
CGACAACGACTCCGATGACGAGGAGATGATGGTGGAGGAGGCGGAGGAG
GAGGAGGCGACCTCCGAGCACAAGCCGTTCGTCTTCCGCGCCAAGAAGGC
GGCGGCGGCGGCGTCGAGCAGGCGCAGGAAGCCGGCGCAGTACAGGGGC
GTGCGGCGCCGGCCGTGGGGGAAGTGGGCGGCGGAGATCCGCGACCCCG
TCAAGGGCATCCGCGTCTGGCTCGGCACCTTCACCAACGCCGAGGCCGCC
GCGCTCGCCTACGACGACGCCGCGCGCGCCATCCGCGGGGACAGGGCCAA
GCTCAACTTCCCTTCCGCTACCACCCCTGACACCCGCAAGCGCGGCCGCGC
CACCGCCGCCGCCGCCCCGGCCGTCAAGGCGACCCCGGTCATCAACCTCG
TCGAGGAGGAGGACGAGGAGGAGGTCGCCGCCGCCATGGCGTCCATCAA
GTACGAGCCCGAGACCAGCGAGAGCTCCGAGTCGAACGCCCTCCCGGACT
TCTCCTGGCAGGGCATGTCGGCCTCCGACGAGTTCGCCGTCGCCGCGGCG
GCGCTGTCGCTCGACAGCGACGACGACCTCGCCAAGAAGCGTCCGAGGAC
CGAGCCGGAGGACACCACCGACTCCGGCTCCGGCGACGACACCGACGCG
CTGTTCGACGCGCTGCTGTTCGCCGACCAGTACAACCACTTCAACGGCGGC
GCCTACGAGTCCCTGGACAGCCTGTTCAGCGCCGACGCCGTGCAGACCAC
CGCCGCCGCCGCCGCCGCCGACCAGGGCATGGGGCTCTGGAGCTTCGACG
ACGGCTGCTGCCTCGTCGACGTCGAGGCCAGCTTGTCCTTCTAG
Os10g0390800
ATGTCTCAAACCCAATCAAACTCCAACCAAACTCACCTACCTACCCCCAAC

CCATCCAGAGCTAGAGCTATGTGCGGCGGCGCGATCCTCGCCGACCTCATA
CCGTCGCCGCGCTCCGGCGGCCACACCAAAAAGAACAAGCGGCGGCGGA
TCAGCGACGACGAGGACTTCGAGGCCGCCTTCGAGGAGTTCGACGCCGGC
GACGACGACTCCGACTCCGACTCCGAGTCCGAGGAGGTAGACGAGTACGA
CGTCGTCGTCGACGACGACGACAGCGAGGACGGCGTGGTGGTTCTTCCGC
CGCCGCCGCCGCCGCCGCCGGTGATTCCACATGAGCGCCATGGCGCGAGG
CGGTTCCGCGGCGTGAGGAAGCGGCCGTGGGGGAAGTGGGCGGCGGAGA
TCCGCGACCCCGTGCGCGGCGTGCGCGTCTGGCTCGGTACCTTCCCCACCG
CCGAGTCCGCCGCGCGCGCCTACGACGCCGCCGCCCGCCGCCTCCGCGGC
GCCAAGGCCAAGCCCAACTTCCCCTCCGCGCCGCCGCCCTCGGCTGCTGC
TCACCGCCGCAAGAAGCGCCGCGCCCACGCCGCCACGCGCTCGCCGTCGT
CTCCGCCCGCCACCAGCGAGGTCACGGCGGCGTCCGCGTCCGCGTCCAGC
GATGTCCCCGCGCCGGCGTTCGCTTCCTTCGTCGGCGAGCCCGGGCACGGC
GGCGCCAAGTCGATGCCGACGACGAGCCACACCTCGCAGCCAGCCCCGCC
GGCGACGGTGGCGTCCGAGAACGTCGACGACCCGGAGGTGTTCGACCCGT
ACGACGTCCACGGCGGCCTCGCCTCCTACTTCGCCGGCGGCGCGTACGAG
TCCCTGGAGAGCCTGTTCGCGCACGGCGGCGACAGCGCCGCCGTCGACCA
AGCGGCGAGCGACCACTGGCCGGCGGCGCTATGGAGCTTCGCAGACGACG
GCTCGTTCTGCTTCTGA

Os03g0183200

ATGTGTGGCGGCGCGATTCTGGCTAACATCATACCGGCCACGCCGCCGCGG
CCCGCCACGGCGGCGCATGTGTGGCCCGGCGGCGACGGGGAGAAGCGGC
GGAAGGTTGGTGGAGGCGGGTGTGATGACGACTTCGAGGCGGCGTTTGAG
AGATTCGGACGTGAGGACTCTGAGATGGAGGAGGAGGAGGTGGAGGAGG
TGGTGGTTGGGAAGAAGGCGGCGGTGAGGCGGCGGAGGGCGACGCCCGC
CGCCGGGCGCCGCGCGAGGCCGAGCAAGTACTGGGGCGTGCGGCGCCGG
CCGTGGGGGAAGTGGGCGGCGGAGATCCGCGACCCCGTCGAGGGCGTCC
GCGTCTGGCTCGGCACGTTCGCCACCGCCGAGGCCGCCGCCCACGCCTAC
GACGCCGCCGCCCGCGACCTCCGCGGCGCGACCGCCAAGCTCAACTTCCC
CTCCTCCTCCTCCTCCACCGCCGCCACCCCACGCCCCCGCAAGTGCCGCCC
CACCACCGCCACCGCCACCCCCAAGGCGACGACACCGAACGTCGTCGTCG
TCGTCAACCTCGTCGACAAAGAGGCCGAGGTCAGCGAGAGCTCCGGTGCC
AGCAGCAGCGCGCTGCCGGACTTCTCGTGGCAGGGCATGTCGGCGTCGTC
CGACGACGACGCCGCGGCGCAGCAGGCACTCCTCGACGCCGCCGGCGGC
GCCAAGAAGCGTCCCCGGAGCGAGCCCCACGTCACCTCCGACGACGAAGT
GCTCCCGGCGTCATTCGACAGTGACAACAACACCGCCGCCGCCGGCCTGC
TCCCGCTCGACGATCCTTTCTTGTTCGGCGACCAGTTCGGCGACCTCAACG
GCGGCGCGTTCGCCTCGCTCATGGACGGGCTGTTCGCCGCCGGTGAAGCG
AACGTCGCCGGCGAGAGCGTGGGGCTCTGGAGCTTCGGCGACGACTTTCT
CAACGCGTCGTACTATTAG

OsSNORKEL1(C9258)

ATGTGCGGAGGTTGTCTCATCCCGGACGAGCTGGTCGGTAAGCCGGCGAG
GAGGACGCGGGCTGCCGCCGCCGGCGGCGACTCCGGTGATGGATGGAAAC
ATGGACGGCGCCTCTGCCCCGCCGCGGCGCCGTGCAACTGCAAGCCTCGC

CGTCGCGCCGGAGCCGCGGACGACGACGACGTGGGCCGCCGCAGGAGAA
CGACAAGGACGAGGGCGGCCAGCGAGGTGCGGTTCCACGGCATCCACATG
CGCAGCTACGGGCGGTGGTCGGCCGAGATCAGGGACAGCAGCTACAGGGG
CCACCGCGTGTGGATCGGCACCTACGCCACGGCGGAGGCGGCGGCGCGGG
CCTACGATGCCGAGGCCCGCAGGATCCACGGCGCCAAGGCCAACACCAAC
TTCCCCCCGCCGCCGAACGACGTCGACAGCGGTGCGCCGCCGCCACCGCC
GTGGGACCTCGAGGCGCACATGAGGTTCCTCGGCGAGGTGGAGCTGGACG
ACGGCGGAGCTGAGCCGCCGCCGCCTCCGAGTTACGGTATCCCTGAACTAC
TGCCCATGGAGCCGGAGCTCGTGAGCGCGACCCAGTCGGTGCACGGCGAC
GACGAGCCGTGGGGGCTCGACAAGTACATGCGGTTCCTCAGCGAGGTGGA
GCTGGACGACGGCGGAGCACCGCTGCCGCCGCCGCCGAGCCAGCACGGC
GGAGTCGCTGCGGCCGGTAGCCCGCAGTACGGCTGTCGCTACGACTACCTC
CTGCTGATGATGTGCAATTAA

OsSNORKEL2(C9258)

ATGTGCGGAGAGAACGATAACAATGGCGCCGCCGCCGGCAGCAGCCGCCG
GCTGCCGGCGGTGGGGGCAATGCGAGGCCCCTGCATCGAGGAGAAGCTTA
AGACGGTGGTGGTGGTGTTGTCGGACGACGACGACGACTACGAGGAGGA
GTTCCGGAGGTACTGCGAAAACACCACACTCCCGGCCAAAGGTGACAAGG
GCGGGCGACGGCGGCCGGCGGCGAGCAAGAAGCAGCACCGGCACCGCTT
CCACGGCATCCACCGGCGCAAGAGCGGGCGGTGGTCGGCCGAAATTAGGG
ACAACATGATCAAGGGCTCCCGCTCGTGGGTCGGCACCTTCGACACCGCC
GAGGAGGCGGCGTGGGCGTACGACGCCGTCGCGCGCCGCCTCTACGGCCC
CAACGCCCGGACCAACTTCCCGCTTCCGCCGCCGCCGCCGCCGCCGGTGG
CTCCACTTCTTCCAGCTCCCGCCGTGGCCAACAAGAAGATGAACAGTAAG
AGCAAGAAGCCGGCGCCGAAGATGGTAGTAGCCCCCGCCGGCGGCGAGA
CGGCGGCAGCAGCAGGGGAGATGGCGCCGGTGCTGCTGGGCAACGCACT
GGAGGCAACGAATGGGTGGGAGTTCGAACCCTACAGCTGCATGGGGCTCG
TCGTATGCAGCGCCGTGTACAACTACGCCGACGAGCCGGAGCCGGCGGAT
GATGAGCTCCAGCTGCTGCACCTGATGCACGGCGGCGCCATGGCCGACTTC
GCCGCCGACGGCTGCCTCTGGTCCTTTTAG
